# Improving the comparability of momentary affect assessments and accounting for item-specific effects in within-person affect dynamics

**DOI:** 10.1038/s44271-026-00473-0

**Published:** 2026-08-19

**Authors:** Tobias Koch, Miriam F. Jähne, Kenneth Koslowski, Peter Koval, Tanja Lischetzke, Daniel McNeish, Jana Holtmann

**Affiliations:** 1 Institute of Psychology, Friedrich-Schiller-Universität, Jena, Germany; 2 Wilhelm Wundt Institute for Psychology, Leipzig University, Leipzig, Germany; 3 Melbourne School of Psychological Sciences, The University of Melbourne, Melbourne, VIC Australia; 4 Department of Psychology, RPTU University Kaiserslautern-Landau, Landau, Germany; 5 Department of Psychology, Arizona State University, Tempe, AZ USA

**Keywords:** Development studies, Human behaviour

## Abstract

A primary objective of intensive longitudinal studies is to investigate within-person dynamics. In this context, item heterogeneity plays a critical role, as within-person processes may vary across items within a scale. A common example is the assessment of momentary affect using adjective lists (e.g., sad, angry, anxious, stressed), where each item captures different facets of positive or negative affect, providing unique and non-interchangeable information. However, standard practices often overlook item heterogeneity by aggregating item scores or assuming a single within-person factor in dynamic structural equation models. This simplification does not permit a fine-grained analysis of within-person dynamics and compromises cross-study comparability when item pools differ across studies. In this article, we reanalyze five large-scale intensive longitudinal datasets assessing momentary affect to illustrate how item heterogeneity can be explicitly modeled. We introduce a flexible modeling approach that accommodates item-specific and person-specific dynamics while improving psychometric comparability across studies, based on residual dynamic structural equation modeling with reference items. We compare this method to conventional modeling strategies and provide practical guidance for addressing item heterogeneity in the analysis of intensive longitudinal data.

## Abstract (web summary)

The paper introduces a model for item- and person-specific affect dynamics that improves cross-study comparability when item sets only partly overlap, demonstrated in a reanalysis of five intensive longitudinal datasets.

## Introduction

Intensive longitudinal designs (ILDs), such as experience sampling, daily diaries, or ecological momentary assessment, are becoming increasingly prevalent in psychological research^1^. ILDs allow for in-depth analyses of within-person dynamics as they unfold in participants’ daily lives^2^. The high temporal-resolution data are particularly well-suited for modeling psychological phenomena, such as momentary affect and mood, with strong ecological validity^3^.

Over the last decade, a growing body of ILD studies has investigated how stressors and events shape momentary affect in the short term, how people regulate those changes, and how such patterns differ between individuals or groups (see e.g., ref. ^3^). This has deepened our understanding of stress and well-being^4,5^, enriched emotion regulation research^6^, advanced psychopathology models by uncovering dynamic markers like affective inertia^7–9^ and affective instability^10,11^, and refined personality theories^12,13^.

In psychology, a standard approach for measuring momentary affect is to present participants with a list of emotion-related adjectives. For example, one widely used measure is the Positive and Negative Affect Schedule (^14^, for recent ILD studies using this scale see, e.g., refs. ^15,16^), although several alternative measures are also available (e.g., refs. ^17,18^).

Researchers often tailor the assessment of momentary affect to the specific goals of their study. For example, they may use only a subset of items from established scales to minimize participant burden, resulting in item lists that partially overlap across datasets. This raises concerns about the comparability of results across studies that have assessed momentary affect using different items^19,20^. In a systematic review on scale usage and measurement practices in emotion research, Weidman et al.^19^ observed that emotions were not measured with convergent sets of items across studies, and that items that were used to measure one emotion were not exclusively used for the respective emotion. This observation may be related to a lack of employing systematically developed self-report scales or to a lack of consensus on the scales used to measure within-person emotional experiences^19–21^. However, even if researchers achieve consensus, there may be heterogeneity in the items if the selected adjectives assess different emotional states.

The present study discusses the potential effects that different types of item heterogeneity in the longitudinal measurement of affect may have on substantive conclusions under different modeling choices. We define item heterogeneity as the degree to which different items within a scale capture *distinct* or *non-interchangeable* aspects of a broader construct. Although all items may aim to assess the same general domain (e.g., negative affect), they can reflect different feelings that are neither redundant nor easily interchangeable. For example, feeling angry may be qualitatively different from experiencing anxiety or shame. These feelings may differ in their phenomenal qualities, as well as in their cognitive, behavioral, and physiological correlates (see e.g.,^18,22^). At the most basic level, feelings are considered to vary not only in terms of valence (positive vs. negative) but also arousal or activation, ranging from passive (e.g., sadness) to active (e.g., anger)^23^.

Although qualitatively distinct feelings (e.g., angry, sad, and anxious) can, at some level, be considered facets of a higher-order affect dimension (e.g., negative affect; see ref. ^16^), they are nevertheless qualitatively distinct. As a result, when researchers use heterogeneous item sets to measure a given affect dimension across different studies, this can lead to jingle-jangle fallacies^19^. The Jingle Fallacy refers to the erroneous assumption that two entities or constructs are identical because they share the same name^19^. For instance, two studies may claim to assess a latent dimension called “negative affect”, but in each study, this latent dimension is measured with different items (e.g., sad and lonely in one study, angry and nervous in the other). The Jangle Fallacy refers to the opposite scenario, namely the erroneous assumption that two entities are different because they have different names, even though they are measured with similar item sets (e.g., what one researcher labels “sadness” and another labels “loneliness” may, in fact, be identical constructs). Such jingle-jangle fallacies may hamper meaningful integration of findings in the literature.

The phenomenon of item heterogeneity is conceptually similar to modeling method-specific effects, which is a well-established concept in both longitudinal structural equation modeling^24–28^ and multimethod research^29–32^. In multimethod research, it is widely recognized that non-interchangeable methods (e.g., self-reports, partner reports, and best friend reports) cannot be adequately represented by a single common factor. Instead, additional method-specific factors are required in the model to account for rater-specific variance.

The objectives of this study are threefold. First, we will discuss different types of item heterogeneity and relate them to various modeling choices in the analysis of ILD, illustrating how item heterogeneity manifests or is masked by these modeling choices using empirical data. Second, we introduce a modeling approach that explicitly accounts for item heterogeneity at both the within-person and between-person levels within a Dynamic Structural Equation Modeling (DSEM) framework. Because the item-specific parameters are modeled as random (i.e., person-specific) effects, our approach allows researchers to model heterogeneity both across items and across individuals. Our primary focus is on the structure of interrelations among affect items at the within-person level, which has been largely overlooked in prior research. This approach enables the computation of person-specific indices of item specificity, which can be interpreted as reflecting individual differences in affect differentiation. Second, by incorporating reference items, the model enhances psychometric comparability of within-person contemporaneous item associations across studies, even when item pools only partially overlap. Third, we compare the proposed approach to the use of composite scores and models with a common within-person affect factor in illustrative analyses of five ILD datasets from the EMOTE database^33^.

Our aim is not to critique conventional modeling approaches per se, but rather to demonstrate how item heterogeneity influences estimates of within-person affect dynamics in ILD data. By examining correlations between various outcome variables and within-person affect dynamics across different studies and analytic strategies, we show that a fine-grained analysis of item heterogeneity offers meaningful advantages. Finally, we discuss the strengths and limitations of our proposed approach in relation to alternative modeling frameworks.

### Item heterogeneity

In the context of intensive longitudinal data analysis, at least three distinct forms of item heterogeneity can be studied. These refer to between-person differences in (1) stable person means, (2) contemporaneous within-person relationships among items, and (3) within-person dynamics over time. Our proposed modeling approach enables researchers to investigate these different types of item heterogeneity by means of between-person differences.

#### Item heterogeneity in stable person means

With respect to stable person means, item heterogeneity refers to systematic differences in how individuals, on average, respond to specific affect items. For example, Person A might consistently report higher levels of anger and stress, whereas Person B may tend to report higher levels of sadness and anxiety. Because individuals differ in their typical affective trait profiles, the relationships among affect items also vary at the between-person level. For instance, stress and anger might tend to show a stronger between-person correlation than anger and anxiety, reflecting the way specific feelings co-occur more frequently across individuals. This type of item heterogeneity can be modeled by allowing correlations among item-specific random intercepts. High correlations (approaching an absolute value of 1) indicate low item heterogeneity and would suggest that a common affect factor can be assumed at the between-person level. Oftentimes, the correlations among item-specific random intercepts are considerably lower, indicating item heterogeneity. For example, a correlation of .70 between two affect items at the between-person level implies that only 49% of the variance is shared, while the remaining 51% reflect item-specific, unique variance. Note that recent research suggests that the factor structure of affect items at the between-person level may differ from the factor structure of the same items at the within-person level^16,34^.

#### Item heterogeneity in contemporaneous within-person relationships among items

Contemporaneous item heterogeneity refers to inter-individual differences in the concurrent interrelations among different affect items. For example, one person may easily feel stressed and angry in response to daily hassles, such that these two feelings regularly co-occur in respective situations for this person. Another person does not react to daily hassles but experiences a difficult period at work, with situations which lead this person to concurrently experience high levels of stress and anxiety, but not anger. In contrast, yet another person might regularly feel both sad and angry after arguing with a romantic partner or close friend, but may not report anxiety or stress in these situations. This highlights that individuals can differ substantially in how feelings, as captured by different affect items, manifest and co-occur in a given moment or context. That is, the same situation may elicit different combinations of feelings in different individuals and individuals may also differ in the frequency that they encounter specific situations.

These examples illustrate a characteristic inherent to ILD that distinguishes the question of item heterogeneity at the within-person level from item heterogeneity at the between-person level or in cross-sectional questionnaire data and underlines why the factor structure of affect items may differ between the between- and momentary within-person levels^16,34^. This characteristic is the momentary focus of item formulations in ILD as opposed to global or retrospective ratings. In ILD, the factor structure of affect items at the within-person level depends on the simultaneous occurrence of different feelings in a single moment. While persons who show high levels of anger on average (e.g., within a period of one month) may also show high average levels of sadness (between-person level), these two feelings may not occur simultaneously in any situation. In the same vein, precursory situations, factors that govern the return to baseline levels, or sequelae of one feeling may differ from those of another feeling.

Individuals may also vary in the degree to which they can differentiate between distinct negative or positive feelings. This ability, which concerns the extent to which individuals distinguish between same-valenced affective states, has been termed emotion differentiation or emotional granularity^35,36^. Inter-individual differences in emotion differentiation should manifest in inter-individual differences in contemporaneous associations between items that capture same-valenced but distinct feelings. Thereby, the notion of within-person contemporaneous item heterogeneity aligns closely with the construct of emotion differentiation. A widely used index for emotion differentiation is the intraclass correlation coefficient (ICC) of repeated emotion assessments^36^, which captures the degree of shared variance among repeated emotion ratings within individuals. Higher ICC values indicate lower differentiation, reflecting a tendency to report same-valenced emotions in a highly correlated manner. However, the ICC treats affective states as interchangeable and does not provide information about which specific affective states individuals are more or less able to discriminate (e.g., one person may struggle to distinguish between sadness and fear, whereas another may have difficulty differentiating between feeling anxious and feeling angry). This aspect of emotion differentiation has recently gained increased attention^37^. The investigation of inter-individual differences in contemporaneous item heterogeneity, as proposed in the present study, is conceptually related to treating emotion differentiation as a trait-like variable (as done with the ICC), while also incorporating emerging perspectives on qualitative patterns of emotion differentiation^37^.

#### Item heterogeneity in within-person dynamics across time

Furthermore, individuals differ in how their affective experiences fluctuate over time (i.e., within-person affect dynamics), and these dynamical properties may depend on the items used to measure affect.

First, affective states such as anger, depression, and anxiety may differ in their moment-to-moment persistence or inertia. This refers to the extent to which a feeling at one time point carries over to the next time point and is typically quantified through autoregressive (AR) effects. Furthermore, feelings captured by different items may also differ in their variability across time, oftentimes quantified by the within-person variance or by the dynamic residual variance in a (vector-)autoregressive model (also called innovation variance). That is, while stress may be highly variable from one time point or one situation to the next, sadness may, for instance, be more persistent.

Second, individuals may differ in the degree of their affective inertia and variability, with these differences being item-specific. For instance, Person A may show high moment-to-moment stability in sadness and depression but not in anger, whereas for Person B, the opposite pattern may be observed.

Yet another form of item heterogeneity refers to the between-person associations of individual within-person dynamic parameters (e.g., AR effects) with an outcome variable. That is, the within-person dynamics of different feelings, as captured by different affect items, may be differentially associated with outcome variables. Recent research results stress the importance of considering item-specific effects in outcome prediction^38,39^, for instance in clinical research. This phenomenon may equivalently apply when using the dynamics of different items as predictors for an outcome.

### Common modeling approaches

In practice, item heterogeneity (particularly at the within-person level) is frequently overlooked when researchers adopt one of two common approaches. The first involves averaging across different affect items to create composite scores of momentary negative or positive affect. The second entails modeling a single latent (negative or positive) affect factor at the within-person level, thereby assuming that all items equally reflect a common underlying affective state. Both approaches implicitly assume that the dynamics in the feelings captured by the individual items are perfectly correlated within a person and that items do not function differently at the within-person level.

#### Composite scores for momentary affect measurement

Despite ongoing debates about the validity of composite scores as proxies for latent factor scores^40–44^, their use remains widespread in psychological research. In a systematic literature review, 71.8% of ILD studies on affective experiences were found to use a composite score of negative or positive affect over several affect items (see supplementary material to ref. ^45^; https://osf.io/7qtye). When researchers construct composite scores by averaging across different affect items, using the same scoring process across individuals, they implicitly assume that the items measure the same common affect factor within each individual. This assumption is violated in the presence of within-person item heterogeneity.

The consequences of item heterogeneity in within-person contemporaneous relationships among items for the construction of composite scores (or common factors) are closely related to the issue of measurement invariance across persons^46,47^. In the presence of within-person item heterogeneity, measurement invariance across persons is not a given. In other words, the affect items function differently across individuals, implying that the latent affect factor (and thereby the composite score) does not carry the same psychometric meaning for different people.

Furthermore, as described above, there may be individual differences in contemporaneous associations between affect items and/or in the temporal dynamics and sensitivity to situational effects of different affect items. Computing a composite score may thereby lead to confounded estimates of the AR effect or innovation variance. Consider, for instance, a person that is highly stressed but not sad at one time point and very sad but not stressed at the next. This person may have low AR and high variance in both stress and sadness. Yet, modeling the dynamics of a negative-affect composite score would suggest that this person shows a stable level of negative affect (i.e., high AR and low variability). In such cases, averaging across items can obscure meaningful item-specific dynamics, and may also bias associations among affect dynamics and outcomes of interest.

Note that the use of composite scores also assumes invariance of the internal structure across time (i.e., measurement invariance across time), which might, for instance, be violated due to changes in the factor structure with changing contextual factors or evolving tendencies of careless responding^48–50^. Here, we focus on item heterogeneity and invariance across persons.

#### Common within-person factor

Another common modeling approach is to specify a within-person factor that loads on all affect items in a multilevel latent time-series or DSEM model^51^. This approach allows researchers to account for measurement error in the assessment of momentary affect. Item heterogeneity at the between-person level can be easily incorporated into these models by specifying indicator-specific random intercepts. However, a common within-person factor fails to address item heterogeneity at the within-person level, as it presumes unidimensionality of the items at the within-person level.

When violations of the unidimensionality assumption occur, item-specific effects may be subsumed within other model parameters in a DSEM. If distinct items differentially capture momentary affect within individuals, this variability must be reflected in model parameters that are mathematically linked to the covariances and variances of the observed items. Contemporaneous within-person correlations between items are reflected in the configuration of standardized random loadings, while the within-person variance of an item is determined by the square of the loading and the corresponding error variance. Thereby, these effects can manifest in the factor loadings and measurement error variances, when these parameters are permitted to vary across individuals.

Apart from potentially disregarding inter-individual differences in the internal structure, the factor approach has one advantage over using composite scores (i.e., summing or averaging over the items). Using composite scores assumes that each affect item contributes an equal amount of information to the negative affect construct^41^. This assumption might even be violated when individuals show the exact same loading pattern across items. The common within-person factor approach does not make this assumption.

Whether the assumption that several affect items are well represented by one common underlying factor is reasonable depends on the specific items that are analyzed and is an empirical question. Dynamic factor models and latent factor time-series models are of great value for modeling within-person dynamics while also correcting for measurement error, given that the specified factor model fits the data and items in a given study. In the empirical application, we illustrate how contemporaneous item heterogeneity may manifest in the common factor model.

In addition to aspects related to heterogeneity in within-person contemporaneous relationships among items, the disadvantages with respect to disregarding inter-individual differences in the items’ dynamics across time (AR effects, innovation variances), as discussed in the previous section, still hold in the factor model in the same way as when using composite scores.

## Modeling item-heterogeneity using a DSEM reference-item approach

Numerous modeling approaches have been developed to account for item heterogeneity (or specificity) in structural equation modeling^24,26,28^. The approach that we propose in the following builds on the work of Eid and colleagues^29,52,53^, which relies on the use of so-called reference items. This strategy is well established in multimethod research^29–32^, latent state-trait modeling^24–27^, bifactor modeling^54^, and missing data analysis^55^, and it offers several advantages. For instance, it enables researchers to (1) explicitly model item heterogeneity by means of latent residual factors at both the within- and between-person levels; (2) include time-invariant or time-varying predictor variables to explain item heterogeneity across levels; (3) compute variance coefficients (or indices) that quantify the extent of item heterogeneity; and (4) compare results across different studies even when non-reference items are added to, or removed from, the overall model.

The model proposed in the following is a multilevel time series model, falling under the umbrella of (residual) DSEM^51,56^. Each observation *y*_*k*,*it*_ for item *k* at time *t* is clustered in the individual *i*. Thereby, each observation *y*_*k*,*it*_ can be decomposed into a stable person-specific latent mean (random intercept/latent trait variable), *μ*_*k*,*i*_, and a latent mean-centered within-person variable ${y}_{k,it}^{w}$, capturing the time-specific deviation of momentary affect from the latent person-mean:

1 $$\left[\begin{array}{c}{y}_{1,it}\\ {y}_{2,it}\\ {y}_{3,it}\\ \vdots \\ {y}_{k,it}\end{array}\right]=\left[\begin{array}{c}{\mu }_{1,i}\\ {\mu }_{2,i}\\ {\mu }_{3,i}\\ \vdots \\ {\mu }_{k,i}\end{array}\right]+\left[\begin{array}{c}{y}_{1,it}^{w}\\ {y}_{2,it}^{w}\\ {y}_{3,it}^{w}\\ \vdots \\ {y}_{k,it}^{w}\end{array}\right]$$

The temporal affect dynamics are modeled for the ${y}_{k,it}^{w}$ variables at the within-person level. In the following, we will conceptually describe the model. A formal definition of the model is provided in Section 1 of the Supplementary Materials..

**Fig. 1:**
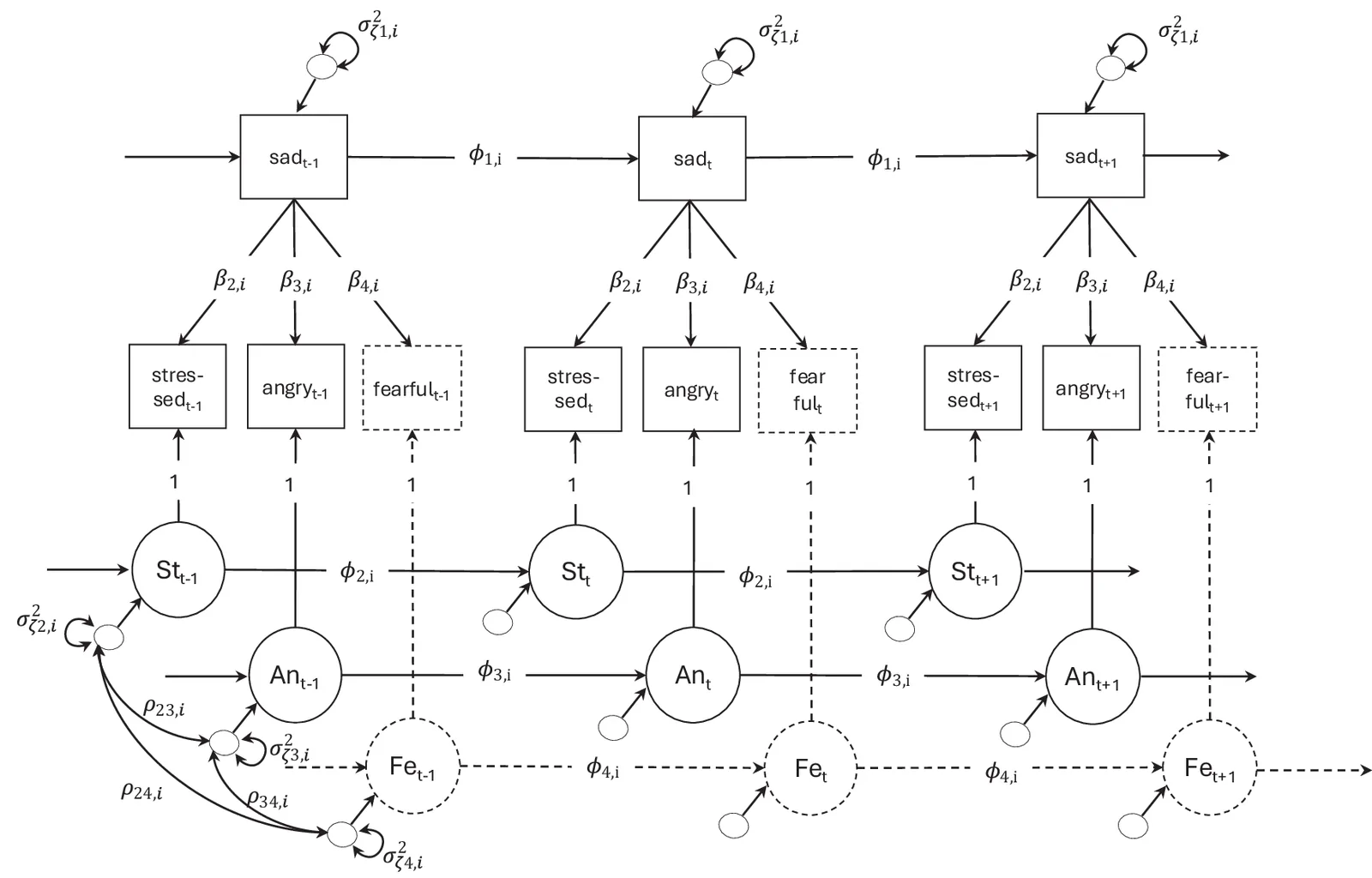
Path diagram of the within-person level of the proposed model for assessing item heterogeneity. In this example, the item sad is selected as the reference item, while the items stressed (St), angry (An), and fearful (Fe) are selected as non-reference items. The regression coefficients *β*_*k*,*i*_ for non-reference item *k* and individual *i* capture the individual-specific contemporaneous associations between the items. The regression residuals *St*_*t*_, *An*_*t*_, and *Fe*_*t*_ are allowed to correlate within time points *t*. Autoregressive effects *ϕ*_*k*,*i*_ for the non-reference items are placed on the items' residuals. *t*: time point; *i*: individual, ${\rho }_{k{k}^{{\prime} },i}$: residual innovation correlation between non-reference items *k* and ${k}^{{\prime} }$; ${\sigma }_{\zeta k,i}^{2}$: residual innovation variance for non-reference item *k*.

Figure 1 presents a path diagram of the within-person part of the proposed model, using four items that are exemplary labeled “sad”, “stressed”, “angry”, and “fearful”. All coefficients indexed by *i* are assumed to vary across individuals, representing person-specific parameters. As shown in Fig. 1, one affect item is designated as the reference item. In our analyses, we selected the item ‘sad` because it was included in most of the datasets on EMOTE. In the discussion, we provide detailed guidelines for selecting appropriate reference items. The remaining affect items (in this case: stressed, angry, and fearful) are modeled in relation to the reference item using linear regressions. The item ‘fearful` is enclosed in a dotted rectangle to indicate that it may be missing from some studies and may be present in others.

At the within-person level, the non-reference items are regressed on the reference item of the same time point (i.e., simultaneous regression), and the residuals from these regressions define item-specific residual factors. These residual factors capture the unique within-person variance of each non-reference item not shared with the reference item (e.g., stressed, after controlling for sad). The standardized regression coefficients *β*_2,*i*_ to *β*_4,*i*_ reflect the bivariate contemporaneous associations between the reference item (e.g., sad) and each non-reference item. Because these coefficients are modeled as random across individuals, they represent person-specific indices of the temporal co-occurrence of specific affective states within an individual and can be interpreted as reflecting lack of differentiation among negative feelings. Larger values indicate lower differentiation, meaning that the non-reference items (here: stressed, angry, and fearful) show high contemporaneous correlations with the reference item (sad) within a person.

The residuals of the within-person regression analysis define item-specific method factors (see latent variables *St*_*t*_, *An*_*t*_, and *Fe*_*t*_). These item-specific residual factors represent the portion of each affect item that is not shared with the reference item (e.g., angry after controlling for sad). Both the reference item and the residualized non-reference items are linked across time through the AR effects *ϕ*_*k*,*i*_. All AR effects are specified as random coefficients, allowing them to vary across individuals. Notably, the AR parameters for the non-reference items can be interpreted as partial temporal stability (inertia) coefficients. They reflect the stability of the unique variance in the non-reference items (stressed, angry, and fearful) after accounting for variance shared with the reference item (sad). If a researcher assumes the presence of cross-lagged effects over time between specific items (e.g., stressed and anxious) after controlling for the reference item, these effects can be added to the model (currently not depicted in Fig. 1).

In addition, the dynamic residuals or innovations *ζ*_*k*,*it*_ capture the influence of momentary situational factors on each non-reference affect item. As with other parameters, all innovation variances (${\sigma }_{\zeta k,i}^{2}$) and covariances are modeled as random effects, yielding person-specific estimates for each affect item. The innovations can be correlated across non-reference affect items within a time point (${\rho }_{k{k}^{{\prime} },i}$). High correlations among innovation components suggest low differentiation between specific non-reference affective states, after controlling for variance shared with the reference state.

The between-person part of the model includes the random intercepts of all affect items as well as the random effects for all within-person parameters (i.e., regression slopes *β*_*k*,*i*_, AR effects *ϕ*_*k*,*i*_, and the logarithms of the residual (co-)variances). These are assumed to be multivariate normally distributed, thereby estimating between-person associations between the parameters. This allows, for instance, to study the between-person relation between item differentiations for different non-reference affect items (e.g., between-person correlation between *β*_2,*i*_ and *β*_3,*i*_ for the items stressed and angry). Note that the between-person level of the model is not shown in Fig. 1, as it corresponds to an unconstrained variance-covariance matrix of all individual-specific parameters (and potential external between-level covariates).

Based on the model parameters, item specificity coefficients can be derived for each individual. These coefficients reflect the extent to which a person differentiates between distinct affective states. Item specificity coefficients can be calculated (a) for the contemporaneous associations among affect items, providing estimates of the degree to which individuals differentiate between the feeling captured by the reference item and feelings captured by the non-reference items; (b) for the time-lagged associations, indicating item specificity with respect to the variance that persists across successive occasions; and (c) for the innovation components, indicating item specificity in the within-person residual variance after accounting for time-lagged effects. In Section 1 of the Supplementary Materials, we provide formulas for computing these person-specific coefficients of item specificity. Higher values in the contemporaneous item specificity indicate, for instance, that the current feeling as captured by a non-reference item is not well explained by the current feeling captured by the reference item, suggesting greater differentiation among these affective states. The counterpart to the item specificity coefficients are the item consistency coefficients, which reflect the amount of shared variance between the non-reference and reference affect items. The square root of the item consistency coefficient can be interpreted as the correlation between affect items (contemporaneous or across time), providing an index of affect non-differentiation.

In practice, researchers can link time-invariant variables to item specificity coefficients to explain individual differences in differentiation among feelings. Alternatively, data-driven clustering approaches may be used to identify individuals with high or low levels of differentiation. In addition, item-specific parameters that reflect different aspects of within-person dynamics, such as AR effects, innovation variances, and covariances, can be directly related to outcome variables to investigate the distinct effects of specific affective states on external criteria. This is particularly important for person-centered health interventions. For example, high temporal stability of a specific feeling, such as sadness, may be associated with adverse clinical outcomes, whereas the dynamics of other affective states, such as stress, may not show similar associations.

Importantly, the psychometric meaning of some model parameters (see Fig. 1) remains invariant even when non-reference items are added or removed from the model. This is the case for the within-person regressions of the items on the reference item, as well as the AR effect and innovation variance of the reference item. As long as the same reference item is used, momentary affect is measured in a comparable manner across studies. Note that the AR effects (and potentially cross-lagged effects) of the non-reference items may change with the selection of items included in the model; comparable to the way they change in a fully-crossed vector-autoregressive (VAR) model when controlling for cross-lagged effects of different variables across time.

It is crucial to emphasize that, using this approach, momentary affect is *not* reduced to a single reference item (in this case, sad). Rather, in the present approach, momentary affect is conceptualized as a *multifaceted* construct in which each affect item contributes unique and meaningful information. The role of the reference item is to anchor the measurement and ensure psychometric comparability across studies.

## Methods

In the following, we illustrate the presence of item-heterogeneity at the contemporaneous as well as the dynamic within-person level and how it manifests when using different analysis strategies. Subsequently, we illustrate the use of item specificity coefficients based on the modeling approach proposed above.

### Data

To investigate item heterogeneity in affect research, we searched the EMOTE database (^33^ Everyday Measure of Temporal Emotions; https://emotedatabase.com), an open-access cumulative repository of ILD studies on daily affective processes, which currently hosts 36 ILD datasets. We included datasets with at least 100 participants and more than an average of 50 measurement occasions per person. These minimal person and occasion sample sizes were chosen based on simulation studies investigating the requirements for accurate parameter estimation in complex DSEM models (see, e.g., refs. ^57–60^). Six datasets met our criteria. Of these six datasets, we excluded one dataset (SEEL: Social Emotions in Daily Life; ref. ^61^) as it did not fulfill the criterion of 50 observed time-points per person after accounting for missed beeps. For the same rationale, in every dataset, we excluded individuals with less than 30 available observations. Also see the respective recommendation by ref. ^62^. Information on the five remaining datasets (sample sizes, available items) is provided in Table 1. Information on the samples (e.g., gender, age, and ethnicity of the participants) can be found in the reference publications provided in Table 1. In total, our sample comprised 802 persons with an average of 111 measurement occasions per person.

**Table 1:** Overview of ESM datasets included in the analyses

Study Name	Negative Affect Items	Positive Affect Items	Outcome Variables (Baseline Measures)	*N* (incl.)	Time points	Reference Publication
Assessing, Understanding, and Regulating Affect (AURA)	Sad, anxious, dull, irritated	Relaxed, peaceful, excited, enthusiastic	Depression, generalized anxiety, rumination, life satisfaction, extraversion, neuroticism, agreeableness	209 (173)	min = 31 mean = 49 med = 50 max = 63	Moeck et al. ^83^
Emotional Events in Daily Life	Sad, angry, stressed	Happy, relaxed	Depression (2x), life satisfaction, well-being, loneliness, self-esteem, rumination, reappraisal, emotion suppression, extraversion, agreeableness	104 (102)	min = 45 mean = 86 med = 90 max = 98	Dejonckheere et al. ^84^
Emotion Regulation Effort (2020)	Sad, angry, anxious, stressed	Happy, hopeful, calm	Depression, generalized anxiety, life satisfaction, extraversion, neuroticism, agreeableness	173 (146)	min = 30 mean = 45 med = 46 max = 56	Hu et al. ^85^
FEEL Study 1	Sad, angry, stressed	Happy, confident, relaxed	Depression, life satisfaction, (social) loneliness (2x), self-esteem, emotional intelligence, rumination, reappraisal, emotion suppression, extraversion, neuroticism, agreeableness	179 (179)	min = 68 mean = 167 med = 173 max = 204	Grommisch et al. ^86^
Leuven 3-Wave Longitudinal Study	Sad, angry, stressed, fearful	Happy, cheerful, confident, relaxed	Depression (2x), borderline symptomatology, reappraisal, emotional suppression, worry, life satisfaction, loneliness, self-esteem, rumination, extraversion, neuroticism, agreeableness	202 (202)	min = 33 mean = 174 med = 185 max = 215	Erbas et al.^87^

Analyses were run separately for positive and negative affect items. We used “sad” as the reference item for negative affect and ‘happy’ for positive affect, as these were the most common affect items across the studies. We did not investigate positive affect for the first dataset (AURA), as it does not contain the item “happy”. All affect items were measured on a slider scale ranging from 0 to 100. In addition to the affect measures, we included (baseline) outcome variables to examine their relationships with affect when using different analysis strategies. Detailed information on the employed baseline scales can be found in a list provided at https://osf.io/z2u43/files/sr35g.

### Analysis strategy

We analyzed the data using DSEM with Mplus version 8.11^63^ (Model D was implemented using Mplus version 9), applying four approaches: random AR effects and random innovation variances estimated (A) separately for each item, (B) for composite scores based on the average of available affect items, (C) for a common within-person affect factor, and (D) for item-specific factors–i.e., our novel analytic approach, illustrated in Fig. 1.

All of the employed models are DSEM models^51^ with the same basic within-between-level decomposition as given in Equation (1). In Model A, we model the within-person dynamics for the time-specific variables ${y}_{k,it}^{w}$ across time separately for each item, using autoregressive models of order 1 [AR(1)]:

2 $${{y}}_{k,it}^{w}={\phi }_{k,i}{y}_{k,i(t-1)}^{w}+{\zeta }_{k,it}$$

Each individual *i* receives an individual-specific AR effect *ϕ*_*k*,*i*_ for each item *k*. Furthermore, the variances of the innovations are allowed to vary across persons, with individual-specific variances ${\sigma }_{\zeta ,i}^{2}$ of the innovations, i.e., ${\zeta }_{k,it} \sim N(0,{\sigma }_{\zeta ,i}^{2})$. Individual innovation variances are assumed to follow a log-normal distribution at the between-person level. That is, at the between-person level, random intercepts *μ*_*k*,*i*_, AR effects *ϕ*_*k*,*i*_, and log innovation variances $ln({\sigma }_{\zeta ,i}^{2})$ are assumed to be multivariate normally distributed, with respective correlations between the individual-specific parameters across persons and items. Note that we use this model solely to obtain between-person correlations for the dynamic parameters of different items. As the model disregards cross-regressive relationships between items across time, we do not recommend to use this model in practice.

In Model B, we use a composite score across the available negative or positive affect items. That is, we have one composite variable *y*_*it*_, which is decomposed as in Eq. (1), with the within-level variable ${y}_{it}^{w}$ being modeled at the within-person level as given in Eq. (2).

In Model C, we assume a common latent within-level factor for the variables ${y}_{k,it}^{w}$:

3 $$\left[\begin{array}{c}{y}_{1,it}^{w}\\ {y}_{2,it}^{w}\\ {y}_{3,it}^{w}\\ \vdots \\ {y}_{k,it}^{w}\\ \end{array}\right]=\left[\begin{array}{c}{\lambda }_{1,i}\\ {\lambda }_{2,i}\\ {\lambda }_{3,i}\\ \vdots \\ {\lambda }_{k,i}\\ \end{array}\right]\left[\begin{array}{c}{\eta }_{it}^{w}\\ \end{array}\right]+\left[\begin{array}{c}{\varepsilon }_{1,it}\\ {\varepsilon }_{2,it}\\ {\varepsilon }_{3,it}\\ \vdots \\ {\varepsilon }_{k,it}\\ \end{array}\right],$$

with individual-specific loading parameters *λ*_*k*,*i*_ and residual variances ${\sigma }_{\epsilon k,i}^{2}$. The within-person dynamics across time are modeled for the common latent factor ${\eta }_{it}^{w}$ according to Eq. (2).

Model D corresponds to the reference-item model as depicted in Fig. 1 and described in Section 1 of the Supplementary Materials.

Models A, C, and D were used to quantify item heterogeneity across all datasets. The between-level correlations of item-specific dynamic parameters in Model A were used to quantify item heterogeneity in within-person dynamics across time. Model C was used to investigate heterogeneity in contemporaneous associations between items by looking at the between-person variability in the factor structure, as quantified by variability in factor loadings and variances. Model D allowed us to compute person-specific coefficients of item specificity (or differentiation).

To illustrate item-specific associations with outcome variables, we linked key parameters (i.e., the AR effects and innovation variances that characterize individual differences in within-person affect dynamics) from Models A, B, and C to relevant outcome variables.

In addition, we examined the robustness of key parameter estimates in Model D by testing whether they remained invariant when non-reference items were added or removed. For this purpose, we exemplarily refitted Model D to the Leuven multi-wave dataset (the largest of the three datasets, including four NA items), excluding one non-reference item at a time.

All models were estimated using Markov-Chain Monte-Carlo sampling, using two chains with a minimum of 10,000 iterations and a thinning factor of 10 (equaling a minimum of 100,000 iterations per chain), assuming convergence when the Potential Scale Reduction factor fell below a cut-off of 1.01 after the minimum number of iterations was reached. Unequal time intervals between observations were handled by using the TINTERVAL option in Mplus, using an interval of 90 min as the desired, approximated time interval between consecutive measurements in all datasets. This value was chosen based on the distribution of the observed time intervals and was closest to the median of the observed time intervals across datasets (with the median time interval ranging from 1.32 to 1.94 h across datasets). Note that by using the same interval for the TINTERVAL option across all datasets, the estimated parameters are to be interpreted with respect to a time interval of 90 min in all datasets and are thereby comparable. Due to skewness in the observed item distributions, we log-transformed the negative affect items in all analyses.

## Results

For brevity, with respect to Models A–D, we focus on the results related to negative affect.

### Items collected in Emote datasets

**Fig. 2:**
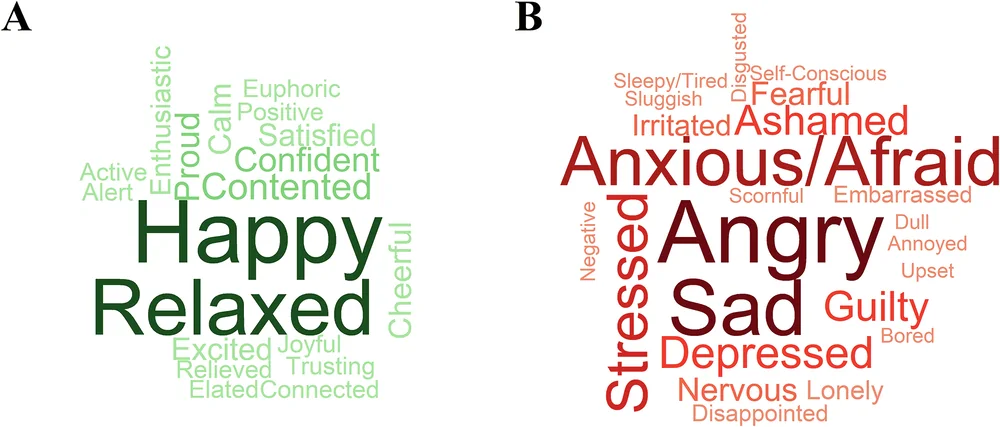
Word cloud depicting the frequency with which negative and positive affect items are used in the intensive longitudinal datasets of the EMOTE database. **A** shows the word cloud for positive affect items and **B** for negative affect items. Frequency is captured by word size, with larger words being used more frequently across datasets.

Previous research on item selection and scale usage in emotion research either included different research designs (not focusing on ILD)^19^ or focused on negative affect^20^. To gauge the extent of heterogeneity in item selection across ILD studies for both negative and positive affect, we reviewed measures of momentary affect across all 36 ILD datasets on EMOTE. Figure 2 displays the frequency of negative and positive affect items used across all ILD datasets (for a detailed summary see Supplementary Table S1. For positive affect, the five most frequently used adjectives are happy, relaxed, contented, proud, and confident. For negative affect, the most common items are angry, sad, anxious/afraid, stressed, and depressed. Across all studies listed on the EMOTE website, we identified only 13 datasets that included the same three negative affect items (sad, angry, anxious) and only 4 datasets that included the same three positive affect items (three out of cheerful, happy, confident, relaxed).

As shown in Fig. 2, some of the affect items used in ILD studies on EMOTE tap into qualitatively similar feelings (e.g., sad and depressed; anxious, afraid, and fearful; sleepy and tired; or enthusiastic, euphoric, and excited). However, many affect items capture qualitatively *distinct* and *non-interchangeable* feelings (e.g., sad vs. angry vs. anxious vs. ashamed), contributing to substantial item heterogeneity. On closer inspection, this heterogeneity is especially pronounced within measures of negative affect, aligning with the general finding that negative concepts tend to be more differentiated than positive ones^64^. This poses a major challenge to anyone seeking to integrate findings across studies that have assessed affect with diverse items. Moreover, this problem is likely to be more prominent in the broader literature, given that all the datasets currently in EMOTE were collected by a relatively small group of related researchers.

### Item heterogeneity in within-person dynamics

With respect to item heterogeneity in the within-person dynamics across time, one approach for empirically evaluating item heterogeneity involves estimating random AR effects and random innovation variances for each affect item separately, followed by examining the correlations among these parameters at the between-person level. If the correlations between item-specific AR parameters and innovation variances are substantially below one (or below the value that we expect based on the parameters’ reliabilities and estimation accuracy), this indicates that the feelings captured by the different items have unique temporal dynamics and situational sensitivities. Therefore, low correlations provide evidence for item heterogeneity in within-person dynamics over time.

**Fig. 3:**
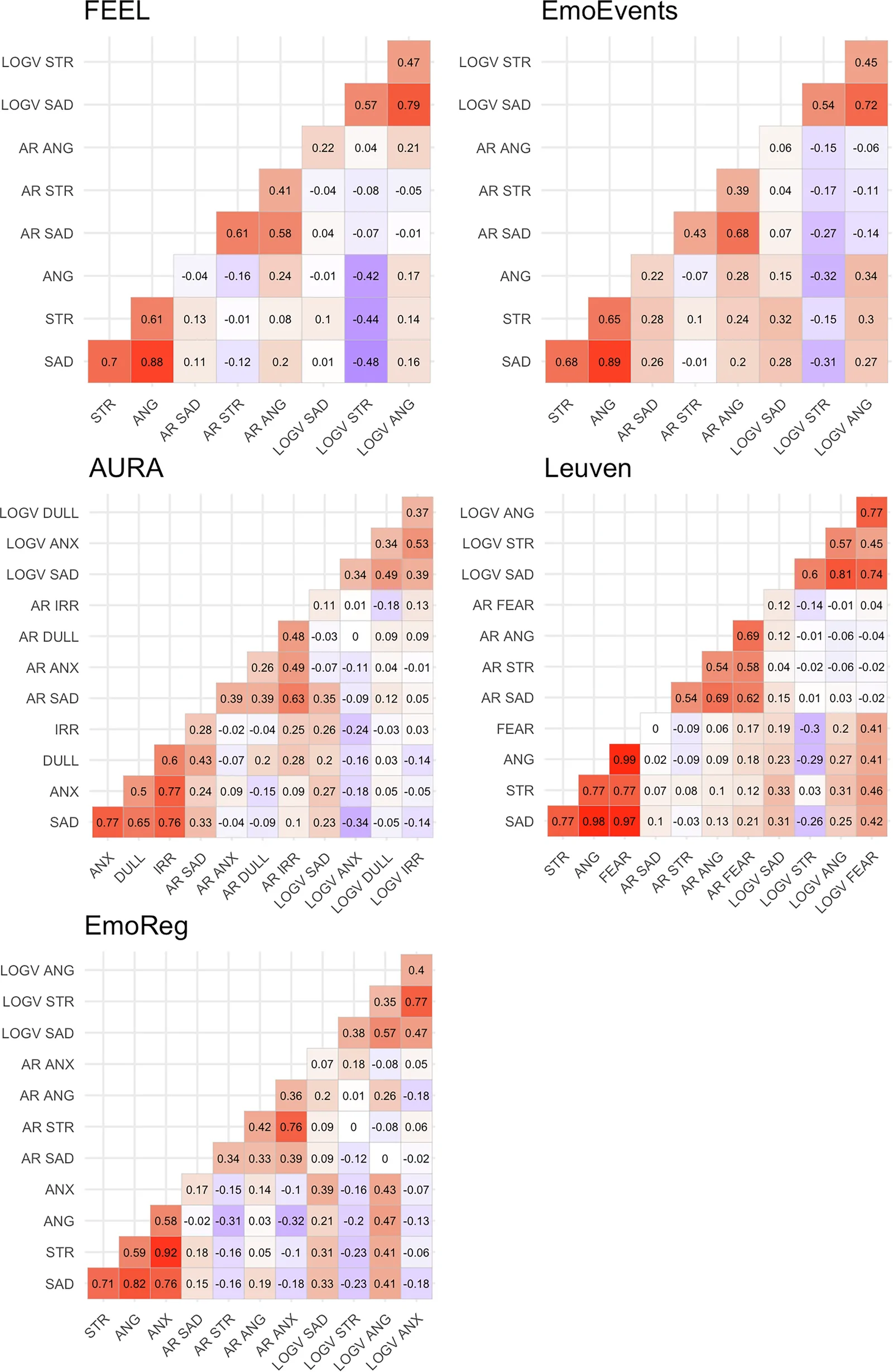
Heatmap of between-person correlations between individual-specific parameter estimates, grouped by dataset. Displayed are correlations between the random intercepts, autoregressive effects, and innovation variances, as estimated from Model A per dataset. AR autoregressive effect, LOGV log innovation variance, SAD item sad, ANX item anxious, DULL item dull, IRR item irritated, FEAR item fearful, STR item stressed, ANG item angry.

Figure 3 shows five heat maps of correlations of random AR effects and random innovation variances of various negative affect items in five EMOTE datasets. The depicted correlations correspond to the between-person correlations as estimated from Model A. Credibility intervals for the presented correlation estimates are provided in Supplementary Tables S2–S6. Supplementary Fig. S1 depicts additional heat maps for positive affect items. As evident from Fig. 3, between-person correlations of the different negative affect items’ random AR effects range between 0.261 and 0.764. Similarly, the correlations between the random innovation variances also exhibit notable variability, ranging from 0.338 to 0.807. These correlations indicate that the rank order of individuals with respect to their dynamics varies substantially across negative affect items. These findings indicate that individual affect items may capture distinct within-person processes. When researchers use composite scores in the presence of such item heterogeneity, they implicitly assume that the AR effects and innovation variances of different items are perfectly correlated, which is clearly not the case in our illustrative analyses. This assumption can lead to a substantial loss of information, as composite scores may obscure meaningful differences in how within-person dynamics are expressed across items.

Note that individual AR effects and innovation variances are sample estimates based on a given dataset. Thus, the maximum possible inter-correlations between dynamic parameter estimates depend on their reliability, which increases with the number of observed time points and is negatively affected by measurement error^58,65^. We will come back to the latter point in the discussion section. For two perfectly homogeneous processes, we can expect a between-person correlation between AR and innovation variances, respectively, that mirrors the respective reliability, and will therefore be smaller than one. However, several factors speak against a pure effect of low reliability in the present case. First, the pattern of correlations that we observe in Fig. 3, that is, highly different correlations between item-specific AR effects, shows that the correlation depends on the item combination. Furthermore, the sample sizes present in the utilized datasets (see Methods section) are comparably high and innovation variances were found to be estimated with high reliability in sample sizes as used in the present analyses^58^.

### Item heterogeneity in contemporaneous associations between items

To evaluate the between-person variability of contemporaneous within-person item associations, we fitted Model C, that is, a DSEM with a common within-person affect factor, to the five EMOTE datasets. We allowed the factor loadings and measurement error variances to vary randomly across persons (see methods section for details). The results revealed substantial variability in both loadings and measurement error variances across individuals. Across items and datasets, the central 95% of individual-specific loading parameters were between 0.095 and 3.501, with between-person correlations of the loading parameters of different items ranging from 0.03 to 0.977 (median = 0.370, mean = 0.458). See Mplus outputs on OSF for details on the specific loading patterns across items. These results suggest that the covariation among feelings captured by different affect items at any occasion may vary substantially across persons, as does the internal structure of the negative affect factor. Consequently, the meaning of the latent negative affect factor is not invariant across persons, as it reflects different items to different degrees for different individuals.

However, even disregarding inter-individual differences in the contemporaneous associations between affect items (i.e., in the standardized factor loadings), the low item reliabilities indicate that the included negative affect items are not well represented by one common underlying factor. That is, only a low proportion of variance in the items is explained by the common factor at the within-person level. This item reliability or common, as opposed to unique, variance in the items at the within-person level ranges, on average across persons, from 0.167 to 0.387 (median = 0.2730 and mean = 0.2762 across items and datasets). This result may not be surprising given that the items that we used as indicators for a common factor can not be considered as interchangeable measures of the same construct, but qualitatively distinct facets of negative affect. In a reflective measurement model, the common factor thereby captures whatever the items have in common, while everything unique to an item is subsumed in the residual^66^. It has been argued that a formative measurement model is more suitable when the items cannot be considered interchangeable^66^.

Note that the above results do not at all question the use of dynamic factor models and latent factor time-series models, in general. They rather underline that the items used as factor indicators should be carefully selected and should fulfill the assumption of unidimensionality and interchangeability. For a comparison of the performance of factor models vs. composite scores in individual time series models, when the unidimensionality assumption holds, see, e.g., refs. ^67,68^. Results from these simulation studies suggest that not correcting for measurement error may result in biased parameter estimates (also see refs. ^58,59,65,69,70^), while fitting single-indicator DSEMs with measurement error for composite scores may be a valid alternative in the absence of item heterogeneity^67,68^.

### Associations with outcomes

**Fig. 4:**
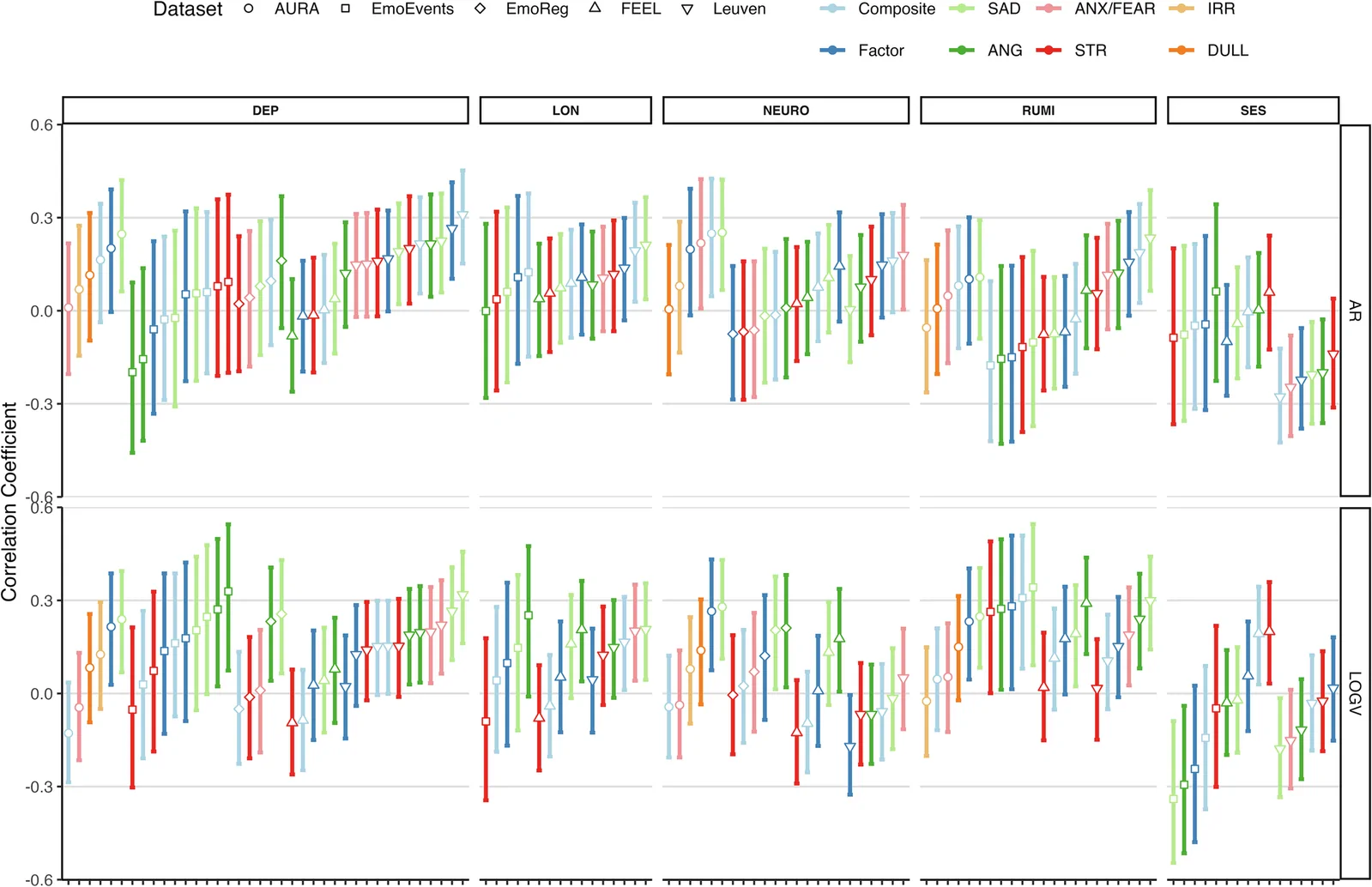
Correlations between dynamic parameters and different outcome variables across different datasets. Depicted are the posterior medians along with 95% credibility intervals of the correlations between multiple outcome variables and random item-specific autoregressive (AR) effects (upper Panels) and the respective log-transformed innovation variances (LOGV, lower Panels), as estimated in Models A, B, and C. The composite score vs. common factor model vs. single items in Model A are color-coded, datasets are represented by symbols, and columns represent different outcome variables. Note that some datasets included two baseline scales for depression. Sample sizes used for the analysis were: AURA *n* = 173, EmoEvents *n* = 102, EmoReg *n* = 146, FEEL *n* = 179, and Leuven *n* = 202. Composite: results based on the composite score calculated by averaging across the negative affect items of a respective dataset in Model B; Factor: results based on a common latent within-level factor model (Model C); ANG angry, STR stressed, ANX anxious, FEAR fearful, IRR irritated, DEP depression, LON loneliness, NEURO neuroticism, RUMI rumination, SES self-esteem.

Next, we examined how affect dynamics correlate with outcome variables (i.e., global measures of depression, loneliness, neuroticism, rumination, and self-esteem). To this end, we used AR effects and innovation variances as estimated in Models A, B, and C. Figure 4 displays these correlations, along with their respective 95% CIs, across all datasets. An analogous figure for positive affect is presented in Supplementary Fig. S2. The results compare estimates based on composite scores or a common factor model with those derived from the model estimating item-specific AR effects and innovation variances.

Overall, the AR-outcome correlations are relatively small, generally falling within the range of −0.3 to 0.3, with only a few 95% CIs not covering zero. A closer inspection reveals that the largest associations between the displayed outcome variables and the item-specific random AR effect estimates are observed for the item sad, sometimes also anxious and angry, while the associations for the AR effects of stressed, dull, and irritated are low across datasets. Across datasets, inertia of the negative affect composite is either weakly correlated with outcomes (in line with most of the AR correlations in the respective dataset) or different from zero along with one or two of the items in the respective dataset. For instance, in the AURA dataset, the AR effect of sad is significantly associated with depression, whereas the AR effect of the composite score is not significantly different from zero, probably due to the fact that it is a blend of the different effects, with the AR effects of the remaining items (dull, irritated, and anxious) not being significantly related to depression. In other instances, a significant relation of the composites’ AR effect with a particular outcome seems to be driven by a single item, e.g., the item sad. A similar picture emerges for the common factor model.

The associations between outcome variables and the log-transformed innovation variances revealed greater differences between analysis methods than those observed for AR effects. Specifically, the innovation variances for angry and sad consistently exhibited stronger positive correlations with depression compared to other individual items or the composite score. In several datasets, these correlations were “statistically significant" (i.e., credible intervals did not contain zero) for angry and sad, whereas the corresponding correlations for other items and the composite or factor score were not. A similar pattern was evident for global rumination, loneliness, and neuroticism, which correlated more strongly with the innovation variances of sad and angry than those of other items. In the Leuven dataset, the innovation variance of the item fearful was associated with higher depression, loneliness, and rumination. Self-esteem showed a negative association with the innovation variance of sad, whereas variability in stress either showed no significant association or a positive correlation with self-esteem, suggesting divergent roles of specific affective states in relation to self-rated outcomes.

### Item-specificity coefficients

**Fig. 5:**
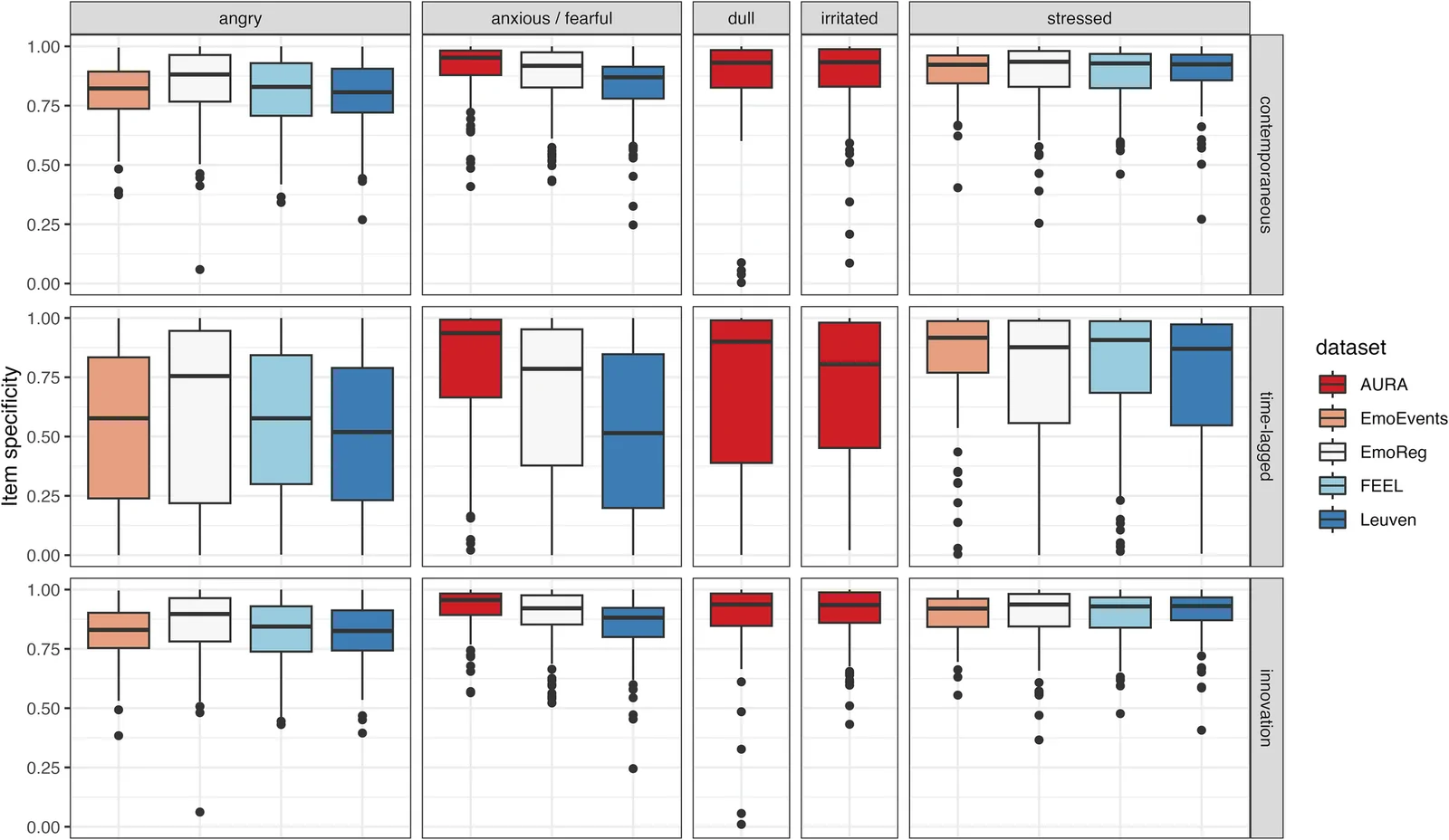
Boxplots of individual-specific item-specificity coefficients. Item-specificity coefficients were estimated using Model D and calculated with respect to the reference item “sad”. Columns present the different non-reference items; datasets are color-coded. Item specificity is calculated for the contemporaneous relations between items, the time-lagged associations, and the innovation components. Boxplots are based on the following sample sizes: AURA *n* = 173, EmoEvents *n* = 102, EmoReg *n* = 146, FEEL *n* = 179, and Leuven *n* = 202.

Figure 5 displays individuals’ item specificity coefficients for the negative affect items across the five datasets. Higher values indicate greater item specificity for a person, or stronger differentiation between negative feelings. All item specificity coefficients were calculated based on Model D, using the item “sad” as the reference item. That is, item specificity refers to the specific variance in the respective item that is not shared with the item “sad”.

According to the boxplots, most individuals show a high degree of differentiation between contemporaneous negative affect states. The first quartile of the individual contemporaneous item specificity coefficients is consistently above 0.70. This indicates that among more than 75% of individuals, the non-reference items share 30% or less of their variance with the reference item (sad). The mean contemporaneous item-specificity coefficients-averaged across individuals and datasets were 0.812/0.870/0.880/0.878/0.890 for angry, anxious, dull, irritated, and stressed, respectively. The extreme values (see black dots) suggest that a small subset of individuals shows little to no differentiation between negative affect states.

For item-specificity based on time-lagged associations, the interquartile ranges are noticeably wider, indicating greater between-person variability in dynamic item specificity. For example, the interquartile range for the item “angry” spans from approximately 0.24 to 0.84 across studies, while the interquartile range for “stressed” extends from 0.64 to 0.98, highlighting substantial individual differences. Overall, the results indicate that the carry-over effect in “stress” is rather specific to “stress” and only weakly associated with persistent sadness for most of the individuals, while a persistent feeling of ‘anger’ since the last time point is to larger degrees associated with persistent “sadness”.

Based on innovation components (i.e., within-person variance adjusted for temporal inertia), the interquartile ranges of the item specificity coefficients fall approximately between 0.75 and 0.99, with median values often exceeding 0.84. This pattern suggests that temporally unexplained deviations in feeling angry or stressed do not necessarily go along with a respective unpredicted increase or decrease in sadness at the same time point. In other words, most individuals appear to differentiate between situational fluctuations in negative affective states such as anger and sadness, with only a few exceptions.

Contemporaneous item specificity coefficients are substantially correlated at the between-person level. For instance, individuals who show higher differentiation of anger and sadness also tend to show higher differentiation of stress and sadness (*r* = 0. 548 − 0. 691 across datasets). The lowest between-person correlation is observed for item differentiation of “anxious - sad” with “dull - sad” (*r* = 0. 377), and the highest for “anxious -sad” with “stressed - sad” (*r* = 0. 908). Between-person correlations of all item specificity coefficients along with credibility intervals are provided in Supplementary Table S7 and innovation correlations between the non-reference items are depicted in Supplementary Fig. S3. See Supplementary Figs. S4 and S5 for the results for positive affect.

### Comparability of results

It is noteworthy that results based on composite scores offer limited comparability when the set of items used for aggregation differs substantially across studies. In such cases, the composite score may not reflect the same underlying construct, even if it is labeled as “negative affect" or “positive affect". In contrast, the reference item modeling approach improves the comparability of findings as long as the same reference item is used. For example, the concurrent regressions between the reference item (e.g., sad) and a non-reference item (e.g., stressed) should remain invariant, even if other items (e.g., anxious, angry) are added to or removed from the model. Note that our approach requires at least one item, namely, the chosen reference item, to be invariant across individuals. This assumption would be violated if individuals interpret that item in fundamentally different ways. This assumption is not unique to our approach but rather reflects a broader issue in latent variable modeling when evaluating measurement invariance, because latent variable models always require researchers to fix the scale of the latent variable.

Table 2 presents the results of Model D applied to the Leuven multiwave study, in which we fitted three models using partially overlapping item pools. From the full set of affect items (sad, stressed, fearful, and angry), we systematically excluded one item in each model: angry in Model 1, fearful in Model 2, and stressed in Model 3. Despite these variations in item composition, the majority of parameter estimates for overlapping items remained within a similar range across models. Specifically, the random regression coefficients of the non-reference items on the reference item are directly comparable across the models. Note that the other parameters (e.g., AR effects) may vary when cross-lagged effects between the non-reference items are included, changing the dynamics based on the item selection.

**Table 2:** Comparison of average within-person standardized parameters using different item pools

Parameter	Model 1	Model 2	Model 3
$$\underline{{{{\bf{Regression}}}}\,{{{\bf{coefficients}}}}}$$			
stressed on sad	0.29 [0.28; 0.30]	0.29 [0.28; 0.30]	–
angry on sad	–	0.42 [0.41; 0.42]	0.42 [0.41; 0.43]
fearful on sad	0.38 [0.37; 0.39]	–	0.38 [0.37; 0.39]
$$\underline{{{{\bf{Autoregressive}}}}\,{{{\bf{Effects}}}}}$$			
sad	0.32 [0.30; 0.33]	0.31 [0.30; 0.32]	0.32 [0.30; 0.33]
stressed	0.22 [0.21; 0.23]	0.22 [0.21; 0.23]	–
angry	–	0.16 [0.15; 0.17]	0.15 [0.14; 0.16]
fearful	0.15 [0.14; 0.16]	–	0.15 [0.14; 0.16]
$$\underline{{{{\bf{Innovation}}}}\,{{{\bf{Variances}}}}}$$			
sad	0.87 [0.86; 0.87]	0.87 [0.86; 0.87]	0.87 [0.86; 0.87]
stressed	0.83 [0.83; 0.84]	0.83 [0.83; 0.84]	–
angry	–	0.77 [0.76; 0.78]	0.77 [0.76; 0.78]
fearful	0.79 [0.78; 0.80]	–	0.79 [0.78; 0.80]

Notes. Parameter estimates stem from fitting Model D to the Leuven 3-wave study dataset, using different sets of negative affect items. Presented parameters are within-person standardized estimates averaged across persons. Point estimates correspond to the median and 95% credibility intervals, presented in brackets, to the 2.5% and 97.5% quantiles of the posterior distributions.

Table 3 compares the standardized within-person parameters from Model D fitted to all five datasets. Again, most parameter estimates fall within a similar range. Note that parameter estimates may vary across the different datasets due to systematic differences in the samples, cultural or linguistic contexts, or ESM protocols or due to unsystematic sampling variability. Nevertheless, the regression parameters which belong to the same non-reference item, the AR effect of the item sad, as well as the innovation variances can be directly compared across studies, as these parameters have the same psychometric meaning.

**Table 3:** Average within-person standardized parameter estimates from model D for 5 datasets

	Leuven 3-wave		FEEL		Emo-Events		AURA		Emo-Reg. Effort	
Parameter	Est.	95% CI	Est.	95% CI	Est.	95% CI	Est.	95% CI	Est.	95% CI
$$\underline{{{{\bf{Regression}}}}\,{{{\bf{coefficients}}}}}$$										
angry on sad	0.42	[0.41; 0.43]	0.40	[0.39; 0.41]	0.42	[0.40; 0.44]			0.33	[0.30; 0.36]
anxious/fearful on sad	0.39	[0.38; 0.40]					0.24	[0.22; 0.26]	0.30	[0.28; 0.32]
stressed on sad	0.29	[0.28; 0.30]	0.29	[0.28; 0.30]	0.30	[0.28; 0.32]			0.27	[0.25; 0.30]
dull on sad							0.28	[0.26; 0.30]		
irritated on sad							0.28	[0.26; 0.30]		
$$\underline{{{{\bf{Autoregressive}}}}\,{{{\bf{effects}}}}}$$										
sad	0.32	[0.31; 0.33]	0.31	[0.30; 0.32]	0.26	[0.23; 0.28]	0.33	[0.31; 0.36]	0.30	[0.27; 0.33]
angry	0.14	[0.13; 0.15]	0.15	[0.13; 0.16]	0.10	[0.07; 0.12]			0.15	[0.12; 0.18]
anxious/fearful	0.14	[0.13; 0.15]					0.25	[0.23; 0.28]	0.18	[0.15; 0.20]
stressed	0.21	[0.20; 0.22]	0.27	[0.26; 0.28]	0.25	[0.22; 0.27]			0.23	[0.20; 0.25]
dull							0.22	[0.19; 0.25]		
irritated							0.17	[0.15; 0.20]		
$$\underline{{{{\bf{Innovation}}}}\,{{{\bf{variances}}}}}$$										
sad	0.87	[0.86; 0.87]	0.87	[0.86; 0.88]	0.90	[0.88; 0.91]	0.83	[0.81; 0.84]	0.84	[0.83; 0.86]
angry	0.77	[0.76; 0.78]	0.78	[0.77; 0.78]	0.77	[0.75; 0.78]			0.77	[0.75; 0.79]
anxious/fearful	0.79	[0.79; 0.80]					0.82	[0.80; 0.83]	0.80	[0.79; 0.82]
stressed	0.84	[0.83; 0.84]	0.79	[0.78; 0.80]	0.80	[0.79; 0.82]			0.80	[0.78; 0.81]
dull							0.80	[0.78; 0.81]		
irritated							0.82	[0.81; 0.84]		

Notes. Parameter estimates stem from fitting Model D to 5 dataset from the Emote database. Presented parameters are within-person standardized estimates averaged across persons. Point estimates correspond to the median and 95% credibility intervals, presented in brackets, to the 2.5% and 97.5% quantiles of the posterior distributions.

*Est.* estimate, *CI* credibility interval.

## Discussion

The role of item heterogeneity has been largely overlooked in research on the within-person dynamics of momentary affect. In this study, we argue that the common practice of modeling affect dynamics using composite scores–formed by combining multiple adjectives, which potentially capture distinct facets of positive or negative affect–may obscure the unique dynamics of qualitatively distinct feelings (e.g., anger vs. anxiety vs. sadness). As a result, key parameters that characterize within-person dynamics, such as AR effects and innovation (co-)variances, become a blend of parameters which can differ substantially across affect items. Our findings suggest that different affect items may provide unique insights into the temporal and situational dynamics of momentary affect, and that such differences should be explicitly modeled and investigated.

Some of the most frequently applied analysis strategies, however, do not allow for such fine-grained examinations of item-specific within-person processes. These approaches typically rely on averaging across affect items or modeling a common latent factor shared by all items. Both strategies implicitly assume that the within-person dynamics are perfectly correlated across items, thus justifying aggregation or the projection of different affect measures onto a single latent dimension with a common temporal dynamic. Furthermore, many studies tailor their item sets to fit specific research objectives, resulting in variability in the affect items used across studies. Under such conditions, composite scores or common latent factors may not reflect the same underlying construct, thereby undermining the comparability of findings across datasets and studies.

We discussed different types of item heterogeneity that may be present in ILD, the assumptions that different modeling strategies impose with respect to item homogeneity, as well as the consequences when these assumptions are not met. Tables 4 and 5 summarize this discussion and provide an overview of the different types of item-heterogeneity, their meaning, implications, and recommendations for modeling practices.

**Table 4:** Overview of the different types of item heterogeneity in ILD, their implications, and potential modeling strategies (Part 1)

Type of item heterogeneity	Stable person means	Contemporaneous within-person associations between items
**Meaning**	Average (trait) levels or the intensities with which affective states, measured by different items, occur on average across time are not homogeneous across items.	Time-specific fluctuations in an affective state captured by one item do not coincide with time-specific fluctuations in the affective state captured by another item.
**Example**	One person may, on average across time, show high levels of stress and anger, while another person shows high average levels of stress but low average levels of anger.	Sometimes sadness and anxiety co-occur at the same time point, sometimes they don’t. The within-person association between items is low.
**How it manifests**	Low to moderate between-person correlations between average (trait) levels of different items.	Within-person correlations between items are low. In a common factor model, standardized factor loadings are small and item reliabilities are low.
**Implications when ignored**	The model will be too restrictive and likely not fit the data. If a common trait and measurement errors are estimated at level 2, only *i* − 1 measurement errors should be modeled. The measurement errors can be correlated, as they reflect indicator-specific effects.	Composite scores, which average different items’ scores per time point, are uninterpretable blends of different affective states that do not necessarily co-occur within a time point. Common factor models exhibit low factor and high measurement error variances.
**Appropriate modeling strategies**	Models that include item-specific average (trait) levels, that is, item-specific random intercepts: 1. Models with item-specific variables (random intercepts) at the between-level 2. Models with a common and *i* − 1 item-specific method variables at the between-level	1. VAR models for single items 2. Model using reference items (advantage: computation of item heterogeneity coefficients)

**Table 5:** Overview of the different types of item heterogeneity in ILD, their implications, and potential modeling strategies (Part 2)

Type of item heterogeneity	Inter-individual differences in contemporaneous within-person associations	Within-person dynamics
**Meaning**	The affective states captured by different items have differential contemporaneous associations across individuals. This taps into inter-individual differences in emotion differentiation.	The affective states captured by different items have different temporal dynamics across time, which may additionally be person-specific and may show different between-person associations with outcome variables.
**Example**	While for Person A sadness and anxiety often co-occur (at the same time), this temporal co-occurrence is not observed for Person B, who is sad in some situations and anxious in other situations, but does generally not experience both at the same time.	Person A has a high persistence and low variability in sadness over time but high variability in anger. The pattern for Person B is entirely different. Only inter-individual differences in the dynamics of the variable sadness are relevant for predicting depressive symptoms.
**How it manifests**	Within-person correlations between items differ across persons. In a common factor model, factor loadings and measurement error variances vary across persons (indicating that the structure of co-occurrence of the different states captured by the items differs across individuals).	Autoregressive effects or dynamic residual variances differ across items. These differences in dynamic parameters between two items are not perfectly correlated across persons. Associations of the items’ dynamic parameters with an external outcome variable differ across items.
**Implications when ignored**	Composite scores are not comparable across persons. Information on the pattern of co-occurrence of specific affective states is lost. Predominant affective states are not visible.	Autoregressive parameters and dynamic residual variances (i.e., estimates of variability) become uninterpretable blends of item-specific dynamics. Very stable or very instable processes in specific affective states of an individual may be missed as the average dynamic is average. Relevant associations with outcome variables may be overlooked.
**Appropriate modeling strategies**	Models that allow for inter-individual differences in the contemporaneous associations between different items: 1. Common factor models with random measurement model parameters (factor loadings, measurement error variances) (disadvantage: latent factor has a different meaning across individuals) 2. Model using reference items (advantage: computation of item heterogeneity coefficients)	Models that estimate different dynamic parameters for different affective states (as captured by different items), e.g., 1. VAR models for single items 2. Model using reference items 3. Common factor models which additionally include dynamic parameters for item-specific residuals

We introduced a novel modeling approach, which includes one reference item and is based on residual DSEM^51,56^. This model allows researchers to anchor the measurement by selecting an appropriate reference item, contrast the remaining non-reference items against the reference item at the within-person level, and estimate item-specific parameters that capture the unique contribution of each item to within-person affect dynamics. The item-specific parameters are modeled as random (i.e., person-specific) effects and can be linked to external outcome or explanatory variables. Furthermore, the model enables researchers to compute person-specific indices of item heterogeneity (or item specificity coefficients) for contemporaneous within-person associations and for within-person temporal dynamics. These indices provide information about the degree to which individuals differentiate between specific affective states (e.g., feeling sad vs. angry) within and across occasions. Below we discuss the advantages and disadvantages of the presented reference-item approach in comparison to alternative modeling strategies.

To examine the extent of item heterogeneity in affective ILD, we reanalyzed five ILD datasets from the EMOTE database using several modeling approaches. Our results revealed substantial item heterogeneity (or specificity) within persons across all datasets. This indicates that most individuals distinguish between different affective states (such as sad, angry, stressed, and anxious) while only a small number showed low levels of differentiation. This pattern is further supported by the relatively low correlations among random AR effects and random innovation variances across items, which highlight significant variation and minimal redundancy in the within-person dynamics of individual affect items. On average, these correlations were approximately 0.50, suggesting minimal overlap between items when modeling within-person affect dynamics.

Relations with external outcomes showed a mixed pattern when using composite scores, whereas a more consistent pattern emerged when modeling item-specific affect dynamics. Among the individual items, dynamic parameters for sad and angry were most consistently associated with the outcome variables. Notably, averaging across different affect items did not necessarily increase statistical power. In several cases, the composite score failed to yield significant associations, whereas specific items (e.g., sad or angry) showed significant positive relationships with the outcomes. This discrepancy may arise when some items’ dynamics are unrelated or even negatively associated with the outcome, while others exhibit positive associations. In a similar vein, McClure et al.^39^ demonstrated the problems associated with using composite scores for outcome prediction in the presence of unique item-outcome relationships, with a focus on clinical prediction. Also see ref. ^38^, who introduce random item slope regression as an alternative approach for outcome prediction that accounts for the presence of item-specific associations.

Note that differences in item-outcome relations across datasets may also arise from the use of different baseline measures for the outcome variables across datasets. Furthermore, also the outcome measures may not be unidimensional and capture different facets of a construct. For instance, Fried has shown that common depression scales exhibit low overlap in the measured depressive syndromes, encompassing low generalizability of findings obtained with different depression scales^71^. However, in contrast to the assumption of item heterogeneity in EMA items, in global or retrospective measures it is not assumed that all symptoms assessed by different items co-occur simultaneously (but they may, for instance, occur at different time points across a time frame of two weeks) and heterogeneity in the dynamics across time are not of interest.

Computing a composite score may lead to confounded estimates of the AR effect or innovation variance, for instance, when a person switches between high levels of different negative feelings such as stress, anger, and sadness. As is often the case with negative affect, which tends to be skewed^72^, none of the individual feelings are likely to be elevated at all (or most) time points and all of them may be relatively variable. In such cases, averaging across items to form a composite score can obscure meaningful associations between item-specific dynamics and outcomes of interest.

Our modeling approach enhances the psychometric comparability of results, even when item sets differ across studies. This is because the interpretation of key model parameters remains consistent as long as the same reference item is used, regardless of whether other items are added to or removed from the model. In contrast, it is often unclear whether a composite score or a common affect factor at the within-level has the same meaning when item sets differ across studies.

### Choice of reference items

Selecting appropriate reference items is crucial for interpreting the parameters in the proposed model. Researchers may base their decision on the following guidelines.

For reasons of comparability and replicability, researchers should select reference items that have been commonly used in prior studies and are widely represented across datasets. For example, in our analyses, we selected ‘sad’ as the reference item for negative affect and “happy” for positive affect, as these items are among the most frequently used across studies. This will ensure that estimated model parameters will have the same interpretation.

To enhance validity and reliability, researchers may choose affect items that are considered the most valid or reliable indicators of the underlying construct. Although it is often challenging to identify a definitive gold standard when working with single items, substantive knowledge can provide useful guidance. For instance, some items may show stronger associations with relevant outcome variables, while others may have demonstrated low reliability (or limited within-person variability) across studies for examining within-person affective dynamics (see e.g. ref. ^73^).

Furthermore, considering the potential (non)interchangeability of item understanding across languages and cultural contexts, researchers may want to select items that carry similar meanings across different contexts. For a discussion of the interplay between language and emotion we refer the reader to the broad literature on this topic (e.g., refs. ^74–76^). Note, however, that cross-cultural and linguistic differences in item understanding, which may create item heterogeneity across studies, is not specific to the use of reference items but also relevant when using item parcels or factor models.

Alternatively, researchers may select a more general affect item to serve as a salient anchor in the model. Individual items that reflect specific facets of momentary affect can then be contrasted against this reference. For example, the items “positive” and “negative” may be used as general indicators of positive and negative affect, respectively, while more specific items such as “sad”, “angry”, and “stressed” serve as non-reference items (for a similar proposal, see ref. ^73^). In some cases, behavioral (e.g., facial expressions, prosody) or physiological (e.g., cortisol levels, heart rate variability) may also function as reference measures, such as when the primary goal is to compare subjectively reported and objectively measured indicators of affect.

### Relation to other modeling approaches

The proposed modeling strategy is closely related to VAR models. While VAR models focus on cross-lagged associations between different variables across time, the model that we proposed here focuses on contemporaneous within-person associations between different items. That is, the models are suited for different data and substantive research questions. If a researcher wishes to investigate whether variable A predicts levels of variable B at following time points (potentially controlling for further variables), the standard VAR model is the model of choice. Furthermore, if substantial cross-lagged associations are present between all variables (items) across time points, a VAR model may be preferable, as Model D in this paper assumes that the reference item is not predicted by previous levels of the other variables. However, if researchers wish to quantify the degree of differentiation between theoretically distinct feelings and inter-individual differences therein, the proposed model is preferable over the VAR model.

Idiographic dynamic network models can be specified as fully crossed VAR models. However, network models offer an intuitive way to visualize associations between variables at both the temporal level (via lag-1 VAR effects) and the contemporaneous level (via innovations; refs. ^77–79^). Typically, contemporaneous associations among innovations are represented using partial correlations^80^. A key limitation of this approach is that the edges in the network are not invariant to the inclusion or exclusion of items. Our proposed approach (Model D) can be conceptualized as a restricted variant of an idiographic dynamic network model that incorporates reference items. These reference items (nodes) serve as anchors within the network, ensuring that specific edges remain invariant when additional non-reference items (nodes) are added or removed. Moreover, this approach allows for the explicit quantification of item heterogeneity through variance coefficients, which can also be related to external variables. However, network models may be the preferred approach when there is consensus on a fixed and well-justified set of items, and a symmetric model is appropriate due to the absence of reference items - or when centrality parameters such as strength, closeness, and betweenness are of primary interest.

The introduced coefficients of item specificity can be interpreted as indices of non-differentiation between different feelings and are thereby linked to the study of emotion differentiation^35,36^. The individual-specific coefficients are conceptualized as person-level variables, that is, stable trait variables. In this regard, they are related to differentiation indices such as the ICC^36^. However, indices such as the ICC typically aggregate information on differentiation between all similarly-valenced feelings into one single index. The present modeling approach allows for a more fine-grained picture of a person’s affect differentiation, with individual item specificities being specific to the chosen affect items. That is, they are calculated for each non-reference item and capture differentation with respect to the reference item. Thereby, they allow researchers to compare differentiation for different feelings and to examine which specific feelings are better differentiated. This is conceptually similar to the idea of Schmitt et al.^37^, who used Latent Markov Factor Analysis to investigate qualitative emotion differentiation and investigate which specific same-valenced emotions are more or less differentiated.

We would like to stress that we do not criticize the use of composite scores or common factor models in general, but try to raise awareness for the assumptions underlying these approaches. In fact, provided that the items used as indicators for a common factor are homogeneous, a common factor model may even be preferable, as it corrects for measurement error in the observed variables and is more parsimonious. The presented model and its item specificity coefficients may be used in a preliminary modeling step to quantify the degree of heterogeneity in contemporaneous item associations as well as in the item dynamics over time. It may thereby serve to examine the suitability of different modeling approaches, choosing between models with and without indicator-specific factors, or in constructing new affect scales with low item heterogeneity. Our approach can not only be used to quantify, but also to predict and/or explain the observed heterogeneity.

Alternatively, researchers may test whether a common factor model is suitable for their items in a first step. If a common factor model does not suit the data (e.g., in the presence of low variance explained in the items by the factor, indicating high item heterogeneity), or in the presence of large inter-individual differences in factor loadings and measurement error variances, our modeling approach could be applied in the next step. Our approach is preferable if item heterogeneity exists at the within-person level. However, it may fail when item heterogeneity is comparably low for most individuals, or when the model becomes highly complex (i.e., when there are many items and constructs to be measured).

### Limitations

While multilevel latent time series models or DSEM models, which estimate a common latent factor at the within-person level, can account for measurement error in the observed items, this is not the case if the dynamics are modeled directly for the observed items. This is a disadvantage, as measurement error in the observed variables has been shown to negatively affect AR estimates^58,59,65,69,70^ and the predictive power of AR estimates for explaining external outcome variables^58^. Thus, the mixed pattern of associations that we observed between individual AR effects and outcome variables in the present study might be partly driven by low reliability of the AR estimates. Note, however, that using the composite score instead was not advantageous for the items available in the analyses we report here.

Consequently, the use of the proposed model, as well as the use of a fully crossed VAR model for single items, is not recommended for items with low reliability. The problems arising from the low reliability of single items may be circumvented by collecting data on several parallel items per emotion facet or feeling. That is, researchers may, for instance, measure momentary anger with two items that capture the current anger level of a person. The availability of several unidimensional items per emotion facet is the ideal situation for the application of models with within-level latent factors. The proposed modeling approach can easily be extended to a model in which each (i.e., reference as well as non-reference) feeling is measured by several interchangeable indicators.

Furthermore, the modeling approach using reference items is naturally limited in the number of items that may be included in the model, as model complexity increases with the number of items. Note that this is also the case for fully-crossed VAR models. Regarding the number of items that can be included and sample size requirements for accurate parameter estimation, the proposed model is similar to the classic (manifest) VAR model.

It is important to recognize the inherent trade-off between measurement granularity and statistical power. As in related idiographic dynamic network and VAR models, including a large number of heterogeneous affect items increases model complexity and the corresponding data requirements. When researchers expect low item specificity, more parsimonious approaches, such as homogeneous affect scales, common-factor models, or composite scores at the level of activation and valence, may be preferable, provided their assumptions are met. When broader content coverage is desired, we recommend selecting items that capture the far ends of the major affective poles (e.g., depression, anger, stress), using two items for each pole. More generally, our approach may help determine when item-level modeling is justified and when dimension reduction is the more appropriate strategy.

The current manuscript proposes a modeling approach that can contribute to a better comparison of results across EMA studies that used different sets of items. This comparability refers to the psychometric meaning of the latent affect dimension that is measured. Naturally, EMA studies may differ in many additional design and sampling characteristics, such as sampling frequency, study length, or sample populations, which may enhance or compromise the comparability of results across studies.

Furthermore, as a data analysis tool, the proposed approach does not directly address the problems that emerge at the design stage of data collection and concern the validity and reliability of the items employed. Work that addresses the psychometric validation of affective EMA measures and development of standardized measurement instruments is strongly needed. As a start, recent work in this direction suggested ways to explore item performance in EMA studies^81^ and to develop and validate affective EMA measures^73^, or directly developed an EMA item quality assessment tool^82^.

## Conclusion

The present study highlights the importance of considering item heterogeneity in the analysis of affective dynamics across time. We illustrated the effects of item heterogeneity for the estimation of dynamic parameters as well as their associations with outcome variables. Furthermore, we introduced an alternative modeling approach based on reference items, which facilitates comparability across studies with different item pools and allows for a detailed investigation of item specificity or affect differentiation.

## Supplementary information

Transparent Peer Review file

Supplementary Materials

## Data Availability

The data are available upon request from the EMOTE database under data request number 42W09CCNFP.

## Code availability

All code is openly available on OSF at https://osf.io/z2u43. Additionally, to facilitate the implementation of the reference-item modeling approach (Model D) for applied researchers, we provide an exemplary annotated Mplus input on OSF.
