# Supplementary material for: Improving the comparability of momentary affect assessments and accounting for item-specific effects in within-person affect dynamics
Source: Commun Psychol. 2026 Aug 19;4:121. doi: 10.1038/s44271-026-00473-0 (PMC13498562; doi:10.1038/s44271-026-00473-0)
Supplement: Supplementary file 1 [file 44271_2026_473_MOESM1_ESM.pdf]

nature portfolio

Peer Review File

Improving the Comparability of Momentary Affect Assessments and Accounting for Item-Specific Effects in Within-Person Affect Dynamics

Corresponding Author: Professor Tobias Koch

This file contains all editorial decision letters in order by version, followed by all author rebuttals in order by version.

Version 0:

Decision Letter:

** Please ensure you delete the link to your author homepage in this e-mail if you wish to forward it to your coauthors **

Dear Professor Koch,

Thank you for your patience during the peer-review process. I am sincerely sorry for the delay in returning to you with a decision.

Your manuscript titled "Improving the Comparability of Momentary Affect Assessments: The Role of Item-Specific Effects in Within-Person Affect Dynamics" has now been seen by 4 reviewers, and I include their comments at the end of this message. They find your work of interest but raised some important points. We are interested in the possibility of publishing your study in Communications Psychology, but would like to consider your responses to these concerns and assess a revised manuscript before we make a final decision on publication.

We therefore invite you to revise and resubmit your manuscript, along with a point-by-point response to the reviewers. Please highlight all changes in the manuscript text file.

Editorially, we consider it critical that the presentation of the work is considerably improved; this includes making appropriate changes to address the referees' comments. It also includes aligning the work with the journal's precise presentation and reporting requirements. More detail on this is included in the attached checklist. Please shorten the Introduction substantially and do not include results in the Introduction.

Additional guidance is available in our style and formatting guide Communications Psychology formatting guide.

We are committed to providing a fair and constructive peer-review process. Please don't hesitate to contact us if you wish to discuss the revision in more detail.

I am attaching an Editorial Requests Table that details critical reporting requirements for the revised manuscript. Please attend to each item and ensure your manuscript is fully compliant. If your revised manuscript is not aligned with these requests on major issues, such as those concerning statistics, it may be returned to you for further revisions without re-review.

Please submit the following items:

- Revised manuscript
- Point-by-point response to the referees' comments

- Cover letter (as a separate document)
- Nature Research Reporting Summary
- Completed Editorial Request Table (attached).

via this link: Link Redacted .

** This url links to your confidential home page and associated information about manuscripts you may have submitted or be reviewing for us. If you wish to forward this email to co-authors, please delete the link to your homepage first **

We hope to receive your revised paper within 8 weeks; please let us know if you aren't able to submit it within this time so that we can discuss how best to proceed. If we don't hear from you, and the revision process takes significantly longer, we may close your file. In this event, we will still be happy to reconsider your paper at a later date, provided it still presents a significant contribution to the literature at that stage.

We would appreciate it if you could keep us informed about an estimated timescale for resubmission, to facilitate our planning.

We look forward to seeing the revised manuscript and thank you for the opportunity to review your work.

Best regards,

Marike Schiffer

Marike Schiffer, PhD
Chief Editor
Communications Psychology

REVIEWER EXPERTISE:

affect, EMA, SEM (statistics)

REVIEWER REPORTS:

Reviewer #1 (Remarks to the Author):

Manuscript COMMSPSYCHOL-25-0477, titled "Improving the Comparability of Momentary Affect Assessments: The Role of Item-Specific Effects in Within-Person Affect Dynamics" identifies and addresses an important issue in the EMA literature: the non-interchangeability of affect items in EMA questionnaires and its implications for cross-study comparability and generalizability. This is an especially important topic given the field's interest in negative affect. The comments below are offered in the interest of clarifying some of the rationale and implications, as well as making this important contribution to the literature more accessible to a wider audience (particularly research teams relying on composites because they are unfamiliar with dynamic structural equation models or in order to reconcile with an existing literature that primarily is based on composites or single within-person factor scores).

Major points:
1. This manuscript draws attention to the non-interchangeability of items, but the framing seems to ignore the non-interchangeability of people, even at the sample level. This framework assumes that people across regions and continents have a common understanding of what it means to be "sad." It appears that some of these participants received the questionnaires in Dutch and others in English. To address this concern, it would be helpful for the authors to discuss: Who are the people in these studies? What are the authors' thoughts on the (non)interchangeability of items across languages and cultural contexts, and how might this affect present results and future application of this approach?

On a similar note, when choosing reference items for future studies, would it also help to consider the likelihood of shared understanding of the items across different contexts (e.g., is "sad" more universally understood than "stressed" across time and cultures)?

2. While the authors rightly point out the limitations of collecting different subsets of affect items across studies, it is important to keep in mind that although researchers likely selected abbreviated item sets to reduce burden, presumably they also selected these items because they expected that these would be the most relevant for the participants and experiences they

were studying. It's possible that these wouldn't have been the items selected in a different study (because those individuals may not have endorsed them at all, or they may not have varied much in them across observations). It's also possible that the presence or absence of certain items may influence participant responses on other items (e.g., if you are asked about stress and not fear, would you indicate your feelings of fear in the item about stress?). It would be helpful to have the authors comment on the implications of intentional study design for the present study and future applications of this approach.

Given the methods focus of the paper, it may be helpful to consider this from a missingness lens. The demonstration using the Leuven multiwave study (in which the team drew from the full set of affect items assessed [sad, stressed, fearful, angry] and systematically excluded one item in each model and compared the results) is a clever use of the existing data and provides support for their interpretation that the concurrent regressions between the reference item and a non-reference item should remain invariant even if the other items are added/removed from the model. This is not directly comparable, however, to comparing to other studies which included differing items. That is, the items that were not included in a particular study are likely not missing at random – but instead were more likely deliberately omitted (or possibly not considered at all).

3. p. 17: "The first quartile of the individual contemporaneous item specificity coefficients is consistently above .70. This indicates that among more than 75% of individuals, the non-reference items share 30% or less of their variance with the reference item (sad)."

This is a striking observation that could be discussed further. For example, what are the implications of this for the field (i.e., should the authors be making a stronger critique of current approaches)? Could this perhaps serve as a diagnostic test for the acceptability of different analytic approaches (i.e., could a future researcher use a similar preliminary analysis to determine whether it is acceptable to use a composite score in their model)?

4. The paper is quite dense, but there are many examples of clear statements and vignettes that help reorient the reader back to the big picture. It may be helpful to look for opportunities to gather these key insights in order to summarize the paper's central points, for example at the end of the introduction and/or at the start of the discussion. This can help bring the reader back to the core arguments being made. A set of questions that may be helpful in crafting a summary that emphasizes the importance and utility of the current study is below:

1) In the most general sense, what is the overall problem the authors have identified in the literature?
2) What types of incorrect/unsubstantiated assumptions are we making in our treatment of ILD data currently, and what are the implications of each of these assumptions for our results and interpretation?
3) What types of research questions can the approach in the present study allow us to answer? For what questions might it not be appropriate?

Minor points:
1. p. 4 refers to "jingle-jangle fallacies." Can the authors briefly define this for readers who are not familiar?

2. p. 10: It is a bit confusing that results begin to appear prior to the Methods section. Do they need to appear here, and if so, is it possible to provide more framing for the reader as to why these numbers are being reported here?

3. The rationale for the inclusion criteria (100+ participants and 50+ observations) is not entirely clear. Does the modeling approach require it? Or, at least in the case of excluding participants with fewer than 30 observations, is this about excluding those who might be non-representative/didn't follow protocol?

4. Figure 4 appears to be quite important (see major point #3 above), however, it was quite difficult to parse how the patterns shown in the figure translated to the conclusions described in pp. 17-18. Further, the second row in the Figure 4 panels shows the item-specificity coefficients for time-lagged associations between a given affect item and the reference (sad) across studies – but from the scant information in the methods section it is unclear whether the studies shared a consistent sampling frequency such that a lag-1 interval is actually comparable across studies (i.e., were assessments every 2 hours in all studies, or was it the case that lag-1 was an observation 1 hour ago in one study but 4 hours ago in another?).

5. In case the authors wish to say more on this point: This is addressed in part, but an important critique of the composite/single factor (and non-interchangeable items) issue that may matter even more in ILD than in global/retrospective applications (e.g., cross-sectional or longitudinal studies that give participants an instruction to complete the PANAS responding to each adjective "in general" or "over the past two weeks", as opposed to ILD uses that prompt participants to rate affect items "how much you feel this way right now") is that not only are different affect items distinct, but they probably do not occur simultaneously. Given the momentary focus of ILD, feeling angry in the moment (and what precedes it, and how long you remain that way) probably does not happen at the same moment (with the same precursors or sequelae or supports that aid "recovery") as sadness.

Reviewer #2 (Remarks to the Author):

I co-reviewed this manuscript with one of the reviewers who provided the listed reports. This is part of the Communications Psychology initiative to facilitate training in peer review and to provide appropriate recognition for Early Career Researchers who co-review manuscripts.

Reviewer #3 (Remarks to the Author):

This manuscript investigates and proposes a modeling approach to account for item heterogeneity in affect measures in intensive longitudinal studies. The paper addresses an important topic and offers a framework for affect researchers to consider in future analyses. It is well-written and makes a valuable contribution to EMA research on emotions.

That said, I have several concerns and suggestions:
• Major claim about aggregation: The paper's central claim is that negative and positive affect scales should not be aggregated as a composite. This is a significant (though not new) claim and has strong implications on past and future affect research. PANAS and other affect scales have prioritized broad content validity at the expense of homogeneity. Similarly, most heterogeneous scales have behavioral manifestations that occur in different times that could be meaningful to study, but the higher order construct is still something worthwhile studying. Therefore, the conclusion that affect items can never be aggregated to positive and negative seems quite extreme.
People's affective experiences may be different for sadness and anger but they both carry the negative valence. Even if the overlap is modest, it exists and hopefully aligns with the claims made in the original research. Specifically, large item heterogeneity may not negate the effects of "negative affect" as a whole: for instance, one may experience sadness at one point and anger at next but overall, there is still a negative valence lingering throughout and this persistent negative affective experience may be the determinant of ill-health even if sadness and anger do not persist.
• Comparability and granularity: In addition, if each affect terms are to be treated as a different experience, cross-study comparability may become even more difficult. Should we not then draw any definitive conclusions about negative affect in the literature? Also, based on the author's cited resource, "The median number of items used was 8, with a minimum of 1 and a maximum of 52" (https://osf.io/7qtye). Isn't that level of granularity quite extreme? How many items can the model handle without sacrificing power? While granularity may be informative, the aggregation is also helpful particularly if we're not talking about more "basic" emotions (like sadness, anger), but rather general affective tendencies (more mood like items). Is this approach necessary if the affect items are not too heterogeneous? Or if there is also aggregation at the activation level as well as valence?
Also, the problem seems to origin at the research design part where researchers are deciding on items at random without proper psychometric work to validate the affect measure. So, the lack of comparability issue is more to do with the standardization of affect measure. The fix proposed by this paper at the modeling/analytical part may not fully address all the concerns that the authors raised and therefore could warrantee some attentions.

It would be helpful if the authors calibrated their claims with similar considerations.

• Model requirements: Clearer guidelines on the required number of participants, observations, and items would strengthen the paper. Since each affect item is treated as a separate construct, how many items can be included before the model becomes too complex? Perhaps also a guideline for when the model is appropriate vs sticking to aggregation. That may help expand the model's usefulness beyond just affect measures.

• Support for claims: Some of the claims could be further supported by evidence or explained more. Some examples below:
o One of the main demonstrations of item heterogeneity comes from here: "If the correlations between item-specific AR parameters and innovation variances are substantially below one (or below the value that we expect based on the parameters), this indicates that different items capture distinct feelings with unique temporal dynamics and situational sensitivities and thereby provide evidence for item heterogeneity in within-person dynamics over time." This reasoning could be explained more clearly. Specifically, why does this metric operationalize item heterogeneity?
o There are major concerns about measurement error and reliability issues with the suggestion to dilute all the affect items into single items measuring separate constructs. The authors do discuss this issue in their paper, but it would be helpful if they elaborated on them further with some single item reliability research or additional support to back their claims. I also had specific questions about claim below:
"The problems arising from the low reliability of single items may be circumvented by collecting data on several parallel items per emotion facet or feeling. That is, researchers may, for instance, measure momentary anger with two items that capture the current anger level of a person." Again, this to me is indicating that each of the affect items should be treated as a different construct? That makes our affect constructs go from generally 2 (which may be too little) to 50+. Also, the suggestion to add more items does not seem very practical when survey length is such an important consideration in EMA research.

• Figures: I would recommend creating two wordclouds: separate for negative and positive affect items. The figure is less effective representation of how affect is measured when it is not shown separate.
• Clarity of terminology: Some of the terms are not defined when introduced (e.g. random innovation variance) and that may limit the accessibility of the article to a newer audience.
• Code: I expected there to be a code output or tutorial for readers to use in the main paper Appendix. The code is linked in the OSF folder I can see but it would be helpful if the code was explained in the Appendix in the paper.
• This is not necessary to cite but other papers have looked at item performance issues that may also be useful to consider in the paper:
Siepe, B. S., Rieble, C. L., Tutunji, R., Rimpler, A., März, J., Proppert, R. K., & Fried, E. I. (2025). Understanding Ecological-

Momentary-Assessment Data: A Tutorial on Exploring Item Performance in Ecological-Momentary-Assessment Data. Advances in Methods and Practices in Psychological Science, 8(1), 25152459241286877.

Overall, even with these concerns, I like the paper and think it will be a valuable addition to the field.

Reviewer #4 (Remarks to the Author):

The manuscript addresses a highly relevant topic. In intensive longitudinal data (ILD) research, there are indeed many reasons why researchers employ different emotion terms to operationalize their constructs. I am therefore very pleased to see another contribution that directly engages with this issue. The introduction is very clearly written, the discussion of item heterogeneity is well-structured, and the purpose of the study comes across clearly. That said, I have several points of attention that I believe would strengthen the manuscript if it is considered for publication:

Levels of item heterogeneity
The paper introduces three different forms of item heterogeneity, all of which are conceptualized as between-person differences (item specific components for each individual). This framing emphasizes person-specific variance, and the authors highlight inter-individual differences in item parameters that can be captured by the model. However, Brose et al. (2020) also emphasize the role of context, noting that "heterogeneity in content in interaction with situational characteristics prevents common covariation." While the authors briefly mention that multimethod modeling can incorporate time-invariant or time-varying predictors to explain heterogeneity, there is little elaboration on this point. Since the model includes contemporaneous effects, it would be helpful to clarify whether and how contextual variables (e.g., events, stressors) could be specified in the model to account for item-specific variance under different conditions. An example analysis would also be highly appreciated.

Between person model
The authors provide information and a figure on the within person relations. What is the structure of the between person model? Would it be feasible to also add this as a figure to the manuscript for completeness?

Clarification of the jingle–jangle fallacy
The authors mention this concept briefly. It may be useful to explicitly define what is meant here, as not all readers will be familiar with the distinction between "jingle" and "jangle" problems.

Reference items and comparability
The use of reference items is a valuable strategy to create comparability across studies. At the same time, the limitations of this approach in ILD research deserve more discussion. For example, on p.14 the authors state: "As long as the same reference item is used, momentary affect is measured in a comparable manner across studies." Yet comparability in ILD studies is not only constrained by reference items but also by major design differences (e.g., sampling frequency, study length, participant instructions). Is the comparability still given even under different study designs?

Presentation of models and results
The manuscript presents four models: single items, composite score, common factor, and the novel item-specific approach. However, models A and C are already analyzed and results are reported earlier in the text, before the main analysis section. This disrupts the central aim of comparing the four models side by side. It breaks the outline of the manuscript and does not aid the readability. I would recommend restructuring the results section to present all four models systematically in one place, allowing for a more direct and comprehensive comparison.
Additionally, associations with outcomes are not reported for models B and C, which leaves the comparison incomplete. I would recommend that there are comparable analyses for the other models so that there is a clear structure that the reader can follow up on after the expectations they are given by the aim of the study.

Conceptual comparison of models
Models B and C rely on latent variables or composite scores, while models A and D operate at the level of single items. This makes the comparisons somewhat uneven. Since the novel model (D) uses single items as indicators, it seems important to also compare it directly to the single-item alternative that does not account for heterogeneity. Otherwise, the reader may struggle to understand how the new model differs from, or improves upon, existing approaches. Similarly, it remains unclear how the proposed model is conceptually comparable to a composite score or common factor model, and why researchers should prefer it in practice. A more explicit articulation of the applications and implications of each approach would be very helpful.

Implications of item differences for outcomes
The analyses relating the models to depression are an important step. They show that items differ in their associations with depression, but what does this imply? Do we need to use different items for different people, or is there one item that is best across studies? A more explicit discussion of these implications would strengthen the paper considerably.

Depression outcome
A more general point for reflection: the outcome of depression is itself measured through a set of items, each of which may capture distinct facets or contexts of the construct. In other words, the same heterogeneity problem addressed in this manuscript also applies to many widely used psychological scales (Fried, 2017). While this issue may not be fully

resolvable in most research designs, explicitly acknowledging this broader limitation would help situate the current contribution more clearly within the larger measurement and validity literature.
Fried E. I. (2017). The 52 symptoms of major depression: Lack of content overlap among seven common depression scales. Journal of affective disorders, 208, 191–197. https://doi.org/10.1016/j.jad.2016.10.019

Additional comments to Results/Discussion
The discussion section remains quite abstract in terms of what the proposed model adds to the existing toolbox and how it could be implemented in practice. At present, readers may struggle to see the concrete value and applicability of the model beyond the technical demonstration. The results section also leaves room to expand on, for example, what types of research questions it would be most useful for, and how it compares to currently established practices. Adding such depth would considerably strengthen the contribution of the paper.

Overall, this manuscript raises an important and timely issue for the ILD field and provides a valuable contribution. However, in its current form, the structure of the manuscript does not fully meet the expectations set by its stated aims. With more clarity on the model comparisons particularly in terms of presenting and comparing the models, and a deeper discussion of implications and limitations, the paper will provide a comprehensive contribution to the literature.

* TRANSPARENT PEER REVIEW: Communications Psychology uses a transparent peer review system. This means that we publish the editorial decision letters including Reviewers' comments to the authors and the author rebuttal letters online as a supplementary peer review file. However, on author request, confidential information and data can be removed from the published reviewer reports and rebuttal letters prior to publication. If your manuscript has been previously reviewed at another journal, those Reviewers' comments would not form part of the published peer review file.

** Visit Nature Research's author and referees' website at www.nature.com/authors for information about policies, services and author benefits**

Communications Psychology is committed to improving transparency in authorship. As part of our efforts in this direction, we are now requesting that all authors identified as 'corresponding author' create and link their Open Researcher and Contributor Identifier (ORCID) with their account on the Manuscript Tracking System prior to acceptance. ORCID helps the scientific community achieve unambiguous attribution of all scholarly contributions. You can create and link your ORCID from the home page of the Manuscript Tracking System by clicking on 'Modify my Springer Nature account' and following the instructions in the link below. Please also inform all co-authors that they can add their ORCIDs to their accounts and that they must do so prior to acceptance.
https://www.springernature.com/gp/researchers/orcid/orcid-for-nature-research

For more information please visit http://www.springernature.com/orcid

If you experience problems in linking your ORCID, please contact the Platform Support Helpdesk.

Version 1:

Decision Letter:

** Please ensure you delete the link to your author homepage in this e-mail if you wish to forward it to your coauthors **

Dear Professor Koch,

Your manuscript titled "Improving the Comparability of Momentary Affect Assessments: The Role of Item-Specific Effects in Within-Person Affect Dynamics" has now been seen by Reviewers #1,#2,#4, whose comments appear below. In light of their advice I am delighted to say that we are happy, in principle, to publish a suitably revised version in Communications Psychology.

We therefore invite you to revise your paper one last time to address the remaining concerns of our reviewers and a list of editorial requests. At the same time we ask that you edit your manuscript to comply with our format requirements and to maximise the accessibility and therefore the impact of your work.

EDITORIAL REQUESTS:

The manuscript currently contains references to results that are deposited on external repositories. All referenced results from the analyses supporting your interpretation of the data need to be included in the main manuscript or a Supplementary Information file.

The reviewers ask for the inclusion of some additional explanations. As you address these concerns, please keep in mind

that the peer review record will be published alongside your paper, so comprehensive repetitions of your rebutally comments will not be necessary in cases where appropriate changes have been undertaken in the revised paper already.

The Introduction section is excessively long and should be revised to focus on key aspects necessary to understand the motivation behind the work and the background literature. Repetitions can be removed and extensive explanations can be reduced or may be more suitable for the Discussion.

Please review our specific editorial comments and requests regarding your manuscript in the attached "Editorial Requests Table". Please outline your response to each request in the right hand column. Please upload the completed table with your manuscript files as a Related Manuscript file.

If you have any questions or concerns about any of our requests, please do not hesitate to contact me.

SUBMISSION INFORMATION:

In order to accept your paper, we require the files listed here https://www.nature.com/documents/commsj-file-checklist.pdf .

OPEN ACCESS:

Communications Psychology is a fully open access journal. Articles are made freely accessible on publication. For further information about article processing charges, open access funding, and advice and support from Nature Research, please visit https://www.nature.com/commspsychol/open-access

At acceptance, you will be provided with instructions for completing the open access licence agreement on behalf of all authors. This grants us the necessary permissions to publish your paper. Additionally, you will be asked to declare that all required third party permissions have been obtained, and to provide billing information in order to pay the article-processing charge (APC).

* TRANSPARENT PEER REVIEW: Communications Psychology uses a transparent peer review system. On author request, confidential information and data can be removed from the published reviewer reports and rebuttal letters prior to publication. If you are concerned about the release of confidential data, please let us know specifically what information you would like to have removed. Please note that we cannot incorporate redactions for any other reasons.

* CODE AVAILABILITY: All Communications Psychology manuscripts must include a section titled "Code Availability" at the end of the methods section. We require that the custom analysis code supporting your conclusions is made available in a publicly accessible repository at this stage; please choose a repository that generates a digital object identifier (DOI) for the code; the link to the repository and the DOI must be included in the Code Availability statement. Publication as Supplementary Information will not suffice.

* DATA AVAILABILITY:
All Communications Psychology manuscripts must include a section titled "Data Availability" at the end of the Methods section. More information on this policy, is available in the Editorial Requests Table and at http://www.nature.com/authors/policies/data/data-availability-statements-data-citations.pdf.

Please use the following link to submit the above items:
Link Redacted
** This url links to your confidential home page and associated information about manuscripts you may have submitted or be reviewing for us. If you wish to forward this email to co-authors, please delete the link to your homepage first **

We hope to hear from you within two weeks; please let us know if you need more time.

Best regards,

Marike

Marike Schiffer, PhD
Chief Editor
Communications Psychology

REVIEWERS' COMMENTS:

Reviewer #1 (Remarks to the Author):

Thank you again for the opportunity to review Manuscript COMMSPSYCHOL-25-0477, titled "Improving the Comparability of Momentary Affect Assessments: The Role of Item-Specific Effects in Within-Person Affect Dynamics." We consider the presentation of the article and its accessibility to a wider readership to be considerably improved, and we continue to believe that this work will be an important contribution to the methodological literature in this area.

The authors' rebuttals are thoughtful, knowledgeable, and responsive feedback to our concerns. It is not clear, however, that these concerns have been thoroughly addressed in the body of the manuscript itself. Indeed, without specific callouts to pages or line numbers in the revised manuscript (with a few exceptions), we [this reviewer and the reviewer-in-training] spent additional time looking for (and consulting with each other regarding) the relevant information in the manuscript which was satisfactorily addressed in the letter. We have identified some points from their rebuttal that we do not see in the body of the manuscript, but that we determined would be important for all readers. The longer responses from the authors' rebuttal might be worth including as footnotes (if permitted by the journal) or perhaps as a supplement, without these we expect that a naive reader may be as confused or unconvinced on some of these points as the reviewers were on the first submission.

Responses to our Major points:
1. This is a clear and thoughtful explanation. The point that this modeling approach "allows researchers to model heterogeneity across individuals (and items)" is especially important for readers evaluating their "usual" approach and considering following the recommendations. It's not clear that this is explained in the text, however, but it would be a helpful addition.
a. Another important point to consider adding to the main text: "our approach requires that at least one item (i.e., the chosen reference item) is invariant across individuals. This assumption would be violated if individuals interpret this item in fundamentally different ways." Again, this clear statement of the requirements and of the assumptions of this new approach would be very helpful, especially given the authors' critique of the typical approach and disregard for the assumptions which underlie it.
2. Thank you for this explanation. It is not clear that the recommendation for a core/common set of affect items is in the manuscript. If a goal of this manuscript is not simply a demonstration of the issues with composite scales, but instead directing applied researchers to alternatives then clear steps and rationale should be provided and organized in an easily findable manner.
3. Thank you for this explanation, and the text on p. 26 is helpful in communicating this. The directive in response to this question makes the proposed approach much easier to follow.
4. Thank you for your responsiveness, the added framing on pp. 4-5 is especially useful in clarifying the goals of the manuscript. Table 4 is also helpful, and if it is possible to move it earlier in the paper, this may make it even more so (we leave this to the author and editor judgment).
a. That said, why is there no text in the first "Implications when ignored" cell of Table 4? Although the answer may seem obvious, or perhaps irrelevant because researchers using intensive longitudinal data do not often ignore person means in their analyses, but for completeness it is important to articulate.

Responses to our Minor points:
1. Thank you for adding the explanation to the text.
2. We appreciate the restructuring and agree that these sections are much clearer.
3. We appreciate the additions on p. 16 to address this question.
4. We appreciate the added sentence in the Method section.
5. We appreciate the additions on p. 6 and p. 32 to address this question.

We have one other observation from another reviewer's comments:

Under reviewer 3, comment beginning with "Also, based on the author's cited resource…"

The rebuttal notes that "we would recommend using items that assess the far ends of the affective poles (e.g. depression, anger, stress), using two items for each pole." This strikes us as helpful advice, but we do not see it in the body of the manuscript. The authors may consider adding this.

Many of the considerations that we found important would be useful in designing future studies. It may be worth adding a paragraph to the Discussion that includes takeaways, both for researchers analyzing secondary data (likely designed and powered for different research questions) and for researchers designing studies specifically focused on affect-related hypotheses. It is our assessment that the manuscript, if published, would be much more impactful with this inclusion and organization.

Reviewer #2 (Remarks to the Author):

I co-reviewed this manuscript with one of the reviewers who provided the listed reports. This is part of the Communications Psychology initiative to facilitate training in peer review and to provide appropriate recognition for Early Career Researchers

who co-review manuscripts.

Reviewer #4 (Remarks to the Author):

Thank you for the elaborate revision! I believe the authors have addressed the comments well and the manuscript is much more structured and goal directed. I am excited that this topic is getting attention and that solutions are developed. In the future I hope that such a topic could be addressed more straightforward and more applied, so that the burden of taking such analyses up could be reduced for researchers. I hope to see an application or tutorial with more examples for substantive researchers in the future.

Some minor things to be addressed:
p. 5 "In the context of intensive longitudinal data analysis, at least three distinct forms of item heterogeneity can be studied."

As addressed earlier the item heterogeneity dealt with there is only on the between person level. I would therefore consider adding the remark that the three forms discussed are between person level differences.

p. 26 correlations are rounded to 3 digits, while in other places they are rounded to two. It would be recommended to use a common approach?

Table 4 leaves very little margins in the cells. The column names are hard to differentiate.

The authors mention they added annotated code on osf? For intersted readers this should be better indicated - as I was not able to find such annotations?

In the nature of Mplus it is of course unfortunate that the analysis is not easily reproducible and accessible to all readers. A future tutorial would indeed help this barrier.

** Visit Nature Research's author and referees' website at www.nature.com/authors for information about policies, services and author benefits**

Open Access This Peer Review File is licensed under a Creative Commons Attribution 4.0 International License, which permits use, sharing, adaptation, distribution and reproduction in any medium or format, as long as you give appropriate credit to the original author(s) and the source, provide a link to the Creative Commons license, and indicate if changes were made.
In cases where reviewers are anonymous, credit should be given to 'Anonymous Referee' and the source.
The images or other third party material in this Peer Review File are included in the article’s Creative Commons license,

unless indicated otherwise in a credit line to the material. If material is not included in the article's Creative Commons license and your intended use is not permitted by statutory regulation or exceeds the permitted use, you will need to obtain permission directly from the copyright holder.
To view a copy of this license, visit https://creativecommons.org/licenses/by/4.0/

Dear Dr. Schiffer,

Thank you very much for your valuable feedback, detailed comments, and the opportunity to resubmit a revised version of the manuscript. We believe that the manuscript has greatly benefitted from the changes we made based on your and the reviewers' suggestions. In the following, we address your concerns and provide information on which changes we have undertaken in response to these concerns and where to find these changes in the manuscript. Note that we have not marked all changes to avoid clutter, as we revised the entire manuscript extensively. The reviews are printed in black, our responses are printed in blue.

Thank you for your patience during the peer-review process. I am sincerely sorry for the delay in returning to you with a decision.

Your manuscript titled "Improving the Comparability of Momentary Affect Assessments: The Role of Item-Specific Effects in Within-Person Affect Dynamics" has now been seen by 4 reviewers, and I include their comments at the end of this message. They find your work of interest but raised some important points. We are interested in the possibility of publishing your study in Communications Psychology, but would like to consider your responses to these concerns and assess a revised manuscript before we make a final decision on publication.

We therefore invite you to revise and resubmit your manuscript, along with a point-by-point response to the reviewers. Please highlight all changes in the manuscript text file.

Editorially, we consider it critical that the presentation of the work is considerably improved; this includes making appropriate changes to address the referees' comments. It also includes aligning the work with the journal's precise presentation and reporting requirements. More detail on this is included in the attached checklist. Please shorten the Introduction substantially and do not include results in the Introduction.

We have revised the manuscript according to the reviewers' suggestions and comments. We have shortened the introduction and have moved all results to the results section.

We are committed to providing a fair and constructive peer-review process. Please don't hesitate to contact us if you wish to discuss the revision in more detail.

I am attaching an Editorial Requests Table that details critical reporting requirements for the revised manuscript. Please attend to each item and ensure your manuscript is fully compliant. If your revised manuscript is not aligned with these requests on major issues, such as those concerning statistics, it may be returned to you for further revisions without re-review.

We have checked all reporting requirements and adjusted the revised manuscript accordingly.

We look forward to seeing the revised manuscript and thank you for the opportunity to review your work.

Best regards,

Marike Schiffer

Marike Schiffer, PhD
Chief Editor

Communications Psychology

REVIEWER EXPERTISE:

affect, EMA, SEM (statistics)

REVIEWER REPORTS:

Reviewer #1 (Remarks to the Author):

Manuscript COMMSPSYCHOL-25-0477, titled "Improving the Comparability of Momentary Affect Assessments: The Role of Item-Specific Effects in Within-Person Affect Dynamics" identifies and addresses an important issue in the EMA literature: the non-interchangeability of affect items in EMA questionnaires and its implications for cross-study comparability and generalizability. This is an especially important topic given the field's interest in negative affect. The comments below are offered in the interest of clarifying some of the rationale and implications, as well as making this important contribution to the literature more accessible to a wider audience (particularly research teams relying on composites because they are unfamiliar with dynamic structural equation models or in order to reconcile with an existing literature that primarily is based on composites or single within-person factor scores).

Thank you for this positive and constructive feedback.

Major points:
1. This manuscript draws attention to the non-interchangeability of items, but the framing seems to ignore the non-interchangeability of people, even at the sample level. This framework assumes that people across regions and continents have a common understanding of what it means to be "sad." It appears that some of these participants received the questionnaires in Dutch and others in English. To address this concern, it would be helpful for the authors to discuss: Who are the people in these studies? What are the authors' thoughts on the (non)interchangeability of items across languages and cultural contexts, and how might this affect present results and future application of this approach?

Thank you for this valuable remark. To a certain extent, our proposed modeling approach already allows researchers to model heterogeneity across individuals (and items). Specifically, our approach is closely related to idiographic dynamic network models, which enable researchers to study interrelationships among affect items over time while allowing these interrelationships to vary across persons. By including contextual variables (e.g., region, cultural background, native language), researchers can explain between-person differences in the within-person and item-specific networks. However, this requires that researchers aggregate multiple studies (Meta-EMA studies or many labs EMA sudies), where contextual variables have been measured and the set of affects items overlap to some extent. In this case, our framework can be used to detect context-dependent differences in affect networks. Researchers can also apply unsupervised machine-learning algorithms to identify clusters of individuals with homogeneous affect-network structures. Overall, our

approach is less restrictive than traditional dynamic structural equation models, which typically assume a common person-invariant factor structure at the within-person level.

Nonetheless, the reviewer is correct that our approach requires that at least one item (i.e., the chosen reference item) is invariant across individuals. This assumption would be violated if individuals interpret this item in fundamentally different ways (e.g., if "sad" carries different meanings across linguistic or cultural groups). However, this is a general problem of latent variable models when testing measurement invariance. Latent variable models always require researchers to fix the scale of the latent variable. This can be done by either fixing one loading per factor or fixing the variance of the factor. However, one of these restrictions must be implemented, regardless of which modeling approach is chosen. Similarly, also when computing composite scores as averages across items, cross-cultural comparisons face the same difficulties as when looking at single items.

From a substantive perspective, there is a broad literature on the interplay between language and emotion, which we now cite in the revised manuscript:

- https://doi.org/10.3389/fpsyg.2015.00444
- https://doi.org/10.1007/s42761-021-00049-7
- https://doi.org/10.1038/s44271-025-00255-0
- https://doi.org/10.1080/02699931.2025.2549966

Discussing this issue in depth is beyond the scope of our article. Nonetheless, we discuss the reviewer's remark in the revised manuscript (Section: *Selection of Reference Items*).

On a similar note, when choosing reference items for future studies, would it also help to consider the likelihood of shared understanding of the items across different contexts (e.g., is "sad" more universally understood than "stressed" across time and cultures)?

Thank you for this suggestion. We agree. Considering whether potential reference items are understood similarly across linguistic, cultural, and temporal contexts is important. Choosing more universally interpreted items—such as "sad"—can help support the reference-item assumption. We now note this in the revised manuscript.

2. While the authors rightly point out the limitations of collecting different subsets of affect items across studies, it is important to keep in mind that although researchers likely selected abbreviated item sets to reduce burden, presumably they also selected these items because they expected that these would be the most relevant for the participants and experiences they were studying. It's possible that these wouldn't have been the items selected in a different study (because those individuals may not have endorsed them at all, or they may not have varied much in them across observations). It's also possible that the presence or absence of certain items may influence participant responses on other items (e.g., if you are asked about stress and not fear, would you indicate your feelings of fear in the item about stress?). It would be helpful to have the authors comment

on the implications of intentional study design for the present study and future applications of this approach.

Thank you for this thoughtful remark. First, we fully agree that there are valid substantive and theoretical reasons for including particular affect items in a given study, and researchers should justify these choices. Although, it is unrealistic to expect all future studies to use an identical set of affect items, it may be feasible for researchers to include a small number (e.g., 1–2) common reference items. We believe there are several reasons to work toward such agreement.

Second, the replicability of findings depends on selecting items that validly measure affect, and the ability to aggregate or synthesize results (e.g., through meta-analyses) requires some overlap in core affect indicators. If affect items vary entirely from study to study with no shared basis, it becomes unclear whether those studies are measuring the same underlying construct or whether studies measure entirely different constructs. Our approach rejects the implicit assumptions of common affect studies that treat items as if they are interchangeable and suggest an easy way to make results more comparable when item sets only partially overlap.

Third, increasingly efforts are made to define a core set of affect measures that should always be used in affective research. Our modeling approach can be viewed as an appropriate statistical method to analyze such data, where some (but not all) items overlap across studies.

Given the methods focus of the paper, it may be helpful to consider this from a missingness lens. The demonstration using the Leuven multiwave study (in which the team drew from the full set of affect items assessed [sad, stressed, fearful, angry] and systematically excluded one item in each model and compared the results) is a clever use of the existing data and provides support for their interpretation that the concurrent regressions between the reference item and a non-reference item should remain invariant even if the other items are added/removed from the model. This is not directly comparable, however, to comparing to other studies which included differing items. That is, the items that were not included in a particular study are likely not missing at random – but instead were more likely deliberately omitted (or possibly not considered at all).

Indeed, our modeling approach can be viewed from a missing data perspective. For example, reference items are typically used in a planned missing data design, where a cheap method (that does not contain missings) is chosen as a reference method and more expensive methods (containing missings) are analyzed as the non-reference method (see, e.g., Garnier-Villarreal et al., 2014; Graham, 2012; Graham et al., 2006; Lawes et al., 2018).

That said, we do not claim that the regression (or correlation) between the reference item and a non-reference item (that is present in study A, but not in another study B) can be directly compared on a one-to-one basis across these studies. However, the correlation between sad and fearful (both measured in study A and B) can be compared psychometrically, even if another item (e.g., stressed) is missing in A or B. It is assumed that

individuals in study A and B are sampled from the same population and that the items can be treated as structurally different methods. In multimethod research, it is generally unproblematic to vary structurally different methods across studies, as long as the same reference method is used. Moreover, the formal definition of MNAR (missing not at random) implies that missingness depends on the individual's unobserved value on the item in question, not on the *researcher's decision* to include or exclude that item in a given study. So, the key question is whether the presence or absence of item Y1 in study A affects respondents' likelihood of answering item Y2 in study B, had Y1 also been present or absent in study B. We would argue that researchers typically assume non-interference (i.e., no interaction) between responses to different items within and across studies.

Garnier-Villarreal M., Rhemtulla M., Little T. D. (2014). Two-method planned missing designs for longitudinal research. *International Journal of Behavioral Development*, 38, 411-422.

Graham J. W. (2012). *Missing data* (1st ed.). New York, NY: New York, NY: Springer.
Graham J. W., Taylor B. J., Olchowski A. E., Cumsille P. E. (2006). Planned missing data designs in psychological research. *Psychological Methods*, 11, 323-343.

Lawes, M., Schultze, M., & Eid, M. (2018). Making the Most of Your Research Budget: Efficiency of a Three-Method Measurement Design With Planned Missing Data. Assessment, 27(5), 903-920. https://doi.org/10.1177/1073191118798050

3. p. 17: "The first quartile of the individual contemporaneous item specificity coefficients is consistently above .70. This indicates that among more than 75% of individuals, the non-reference items share 30% or less of their variance with the reference item (sad)."

This is a striking observation that could be discussed further. For example, what are the implications of this for the field (i.e., should the authors be making a stronger critique of current approaches)? Could this perhaps serve as a diagnostic test for the acceptability of different analytic approaches (i.e., could a future researcher use a similar preliminary analysis to determine whether it is acceptable to use a composite score in their model)?

Thank you. Indeed, our results suggest that, for most individuals, the degree of item heterogeneity is relatively high. We also agree that using affect items that measure *structurally different* affect states is not ideal and often leads to high item specificity. In such cases, our approach can be used to quantify, predict and/or explain this heterogeneity. We discuss these implications in greater detail in the revised manuscript. However, we aim to take a balanced view and do not seek to overly criticize past or future research. Models that assume common affect factors are more parsimonious and are often desired as they allow for easier interpretation. Computing a mean score across non-interchangeable affect items ignores measurement error, congeneric (potentially person-specific) measurement structures, and indicator-specific method effects and make it difficult to interpret and compare results. Our approach can be used as a preliminary analysis to a) construct new affect scales with low item heterogeneity or b) choose between models with and without

indicator-specific factors. That said, researchers could also set up a factor model for testing whether a common factor model is suitable for their items in a first step. If a common factor model does not suit the data (e.g., in the presence of low variance explained in the items by the factor, indicating high item heterogeneity), our modeling approach could be applied in the next step. Our approach is preferable if item heterogeneity exists at the within-person level. However, it may fail when item heterogeneity is comparably low for most individuals, or when the model becomes highly complex (i.e. when there are many items and constructs to be measured). Please see also our answer to Reviewer 2, who argues that aggregation of different affect items may be defensible.

4. The paper is quite dense, but there are many examples of clear statements and vignettes that help reorient the reader back to the big picture. It may be helpful to look for opportunities to gather these key insights in order to summarize the paper's central points, for example at the end of the introduction and/or at the start of the discussion. This can help bring the reader back to the core arguments being made. A set of questions that may be helpful in crafting a summary that emphasizes the importance and utility of the current study is below:

1) In the most general sense, what is the overall problem the authors have identified in the literature?
2) What types of incorrect/unsubstantiated assumptions are we making in our treatment of ILD data currently, and what are the implications of each of these assumptions for our results and interpretation?
3) What types of research questions can the approach in the present study allow us to answer? For what questions might it not be appropriate?

Thank you for this suggestion. We now include a summary of the main points as well as a Table summarizing the different aspects of item-heterogeneity, modeling choices and recommendations for best practices at the beginning of the discussion section.

Minor points:
1. p. 4 refers to "jingle-jangle fallacies." Can the authors briefly define this for readers who are not familiar?

The Jingle Fallacy refers to the erroneous assumption that two entities are identical because they share the same name. For instance, two studies may claim to assess a latent dimension called "negative affect", but in each study this latent dimension is measured with different items (e.g., sad and lonely in one study, angry and nervous in the other). The Jangle Fallacy refers to the opposite, namely the erroneous assumption that two entities are different because they have different names (e.g., what one researcher labels "sadness" and another labels "loneliness" may in fact be identical constructs).

We have included a short definition of the fallacies in the manuscript.

2. p. 10: It is a bit confusing that results begin to appear prior to the Methods section. Do they need to appear here, and if so, is it possible to provide more framing for the reader as to why these numbers are being

reported here?

We understand the reviewers point. Figures 1 and 2 illustrate common practices in affect research. Researchers often use affect items that only partially overlap between studies (Figure 1), measuring distinct within-person dynamic profiles (Figure 2) that do not perfectly correlate within the person. Figure 1 is merely used to illustrate the problem of differing items across studies and is not really a result of any analysis or model fitting. Nevertheless, we have moved both figures to the results section. To this end, we have entirely re-structured the introduction and the results sections.

3. The rationale for the inclusion criteria (100+ participants and 50+ observations) is not entirely clear. Does the modeling approach require it? Or, at least in the case of excluding participants with fewer than 30 observations, is this about excluding those who might be non-representative/didn't follow protocol?

The inclusion criteria were chosen based on the results of simulation studies that tested the requirements for adequate estimation accuracy in time series models as well as complex DSEMs (see, e.g., Jongerling et al., 2024; Koslowski & Holtmann, 2025; Schultzberg & Muthén, 2018; Schuurman & Hamaker, 2019). While the recommended minimum numbers of persons and (average number of) time points depend on model complexity, N = 100 and T= 50 are a widely described minimum that is still reconcilable with the realities of the observed datasets. That is, when including studies with fewer participants or with a lower average number of time points, we would be less confident regarding the trustworthiness of the results. We now cite some of the above studies to provide a rationale for the decision in the manuscript.
With respect to the exclusion of those participants with fewer than 30 time points, the same rationale holds. On average, participants in the included studies provide data on 45 - 167 observed time points. However, given that we estimate all parameters as random effects, every single participant should have a minimum number of available time points, otherwise model convergence and estimation accuracy will deteriorate. Also see a respective recommendation by Asparouhov & Muthén (2024). Choosing 30 time points as a cut-off for each individual ensures that we have enough data points per person and at the same time only exclude a few persons per dataset. See Table 1 in the manuscript for an overview of the total and the included number of participants per dataset as well as the respective numbers of available time points.

Asparouhov, T. & Muthén, B. (2024). Practical Aspects of Dynamic Structural Equation Models, Version 4. Downloaded from [last accessed January 9th 2025]: https://www.statmodel.com/download/PDSEM.pdf

Jongerling, J, Liu, S & Williams, D R 2024 'Bayesian Multilevel VAR Models with Random Covariance Matrices : Estimation Requirements'. https:// doi.org/10.31234/osf.io/5fk8v

Koslowski, K. & Holtmann, J. (2025). Unique Contributions of Dynamic Affect Indicators – Beyond Static Variability. *Multivariate Behavioral Research*, DOI: 10.1080/00273171.2025.2545367

Schultzberg, M., & Muthén, B. (2018). Number of Subjects and Time Points Needed for Multilevel Time-Series Analysis: A Simulation Study of Dynamic Structural Equation Modeling. Structural Equation Modeling: A Multidisciplinary Journal, 25(4), 495–515. https://doi.org/10.1080/10705511.2017.1392862

Schuurman, N. K., & Hamaker, E. L. (2019). Measurement error and person-specific reliability in multilevel autoregressive modeling. Psychological Methods, 24(1), 70–91. https://doi.org/10.1037/met0000188

4. Figure 4 appears to be quite important (see major point #3 above), however, it was quite difficult to parse how the patterns shown in the figure translated to the conclusions described in pp. 17-18. Further, the second row in the Figure 4 panels shows the item-specificity coefficients for time-lagged associations between a given affect item and the reference (sad) across studies – but from the scant information in the methods section it is unclear whether the studies shared a consistent sampling frequency such that a lag-1 interval is actually comparable across studies (i.e., were assessments every 2 hours in all studies, or was it the case that lag-1 was an observation 1 hour ago in one study but 4 hours ago in another?).

We corrected for unequal time intervals between observations by using the tinterval functionality in Mplus. By using this option, a time grid with a time interval that is to be approximated is imposed on the observed data and missing values are inserted wherever time intervals surpass the desired interval. By using the same approximated time interval in all datasets, the parameters are to be interpreted with respect to the same time interval across all datasets. Thereby, the results from different datasets can directly be compared. We have added a sentence with this information to the methods section.

5. In case the authors wish to say more on this point: This is addressed in part, but an important critique of the composite/single factor (and non-interchangeable items) issue that may matter even more in ILD than in global/retrospective applications (e.g., cross-sectional or longitudinal studies that give participants an instruction to complete the PANAS responding to each adjective "in general" or "over the past two weeks", as opposed to ILD uses that prompt participants to rate affect items "how much you feel this way right now") is that not only are different affect items distinct, but they probably do not occur simultaneously. Given the momentary focus of ILD, feeling angry in the moment (and what precedes it, and how long you remain that way) probably does not happen at the same moment (with the same precursors or sequelae or supports that aid "recovery") as sadness.

Thank you for raising this is very important point. We agree with the reviewer and discuss the implicit assumption of simultaneous occurrence of different affect states in more detail in the revised paper.

We thank the reviewer for his/her valuable remarks, which enhanced the quality of our manuscript.

Reviewer #2 (Remarks to the Author):

I co-reviewed this manuscript with one of the reviewers who provided the listed reports. This is part of the Communications Psychology initiative to facilitate training in peer review and to provide appropriate recognition for Early Career Researchers who co-review manuscripts.

Thank you for your time and effort.

Reviewer #3 (Remarks to the Author):

This manuscript investigates and proposes a modeling approach to account for item heterogeneity in affect measures in intensive longitudinal studies. The paper addresses an important topic and offers a framework for affect researchers to consider in future analyses. It is well-written and makes a valuable contribution to EMA research on emotions.

Thank you for this positive evaluation of our manuscript.

That said, I have several concerns and suggestions:
• Major claim about aggregation: The paper's central claim is that negative and positive affect scales should not be aggregated as a composite. This is a significant (though not new) claim and has strong implications on past and future affect research. PANAS and other affect scales have prioritized broad content validity at the expense of homogeneity. Similarly, most heterogeneous scales have behavioral manifestations that occur in different times that could be meaningful to study, but the higher order construct is still something worthwhile studying. Therefore, the conclusion that affect items can never be aggregated to positive and negative seems quite extreme. People's affective experiences may be different for sadness and anger but they both carry the negative valence. Even if the overlap is modest, it exists and hopefully aligns with the claims made in the original research. Specifically, large item heterogeneity may not negate the effects of "negative affect" as a whole: for instance, one may experience sadness at one point and anger at next but overall, there is still a negative valence lingering throughout and this persistent negative affective experience may be the determinant of ill-health even if sadness and anger do not persist.

We believe there has been a misunderstanding. In our manuscript, we do not claim that affect items can *never* be aggregated. We were unable to find a sentence in the manuscript where this statement or recommendation was made. If the reviewer can point us to a sentence or paragraph where we conclude "that affect items can never be aggregated", we are willing to revise it accordingly.

In our manuscript, we question the psychometric *meaning* and *comparability* of results when heterogeneous (i.e., non-interchangeable) affect items are used and aggregated into a single composite score, especially when the basis of this aggregation differs between studies. We argue that the results become difficult to interpret, compare and synthesize in a *meaningful* way if a varying set of heterogenous affects items are used across different studies. There may be valid reasons for aggregating scores and using them as proxies. We would not disagree with this statement in principle. However, these proxies are not equivalent to a well-defined latent variable. From a psychometric point of view, it is unclear whether two researchers are measuring the *same* latent variable if researcher A averages across anxious, ruminative and sad states, and researcher B averages across angry, stressed and overwhelmed states, even though both are meant to measure (or represent) some negative emotional states. One way to improve comparability would be to enforce standardization and use the same items for aggregation (however, standardization has its own limitations, see our comment below). However, even in this situation, computing composite scores requires the affect items to measure a common factor (i.e., to be one-dimensional). This assumption could be tested. In EMA studies, however, researchers typically do not test this assumption, but simply assume that different affect items measure a common within-person factor. In our manuscript, we provide evidence that this assumption (at least in the studies we investigated) does not hold, and that the meaning of the affect factor differs considerably between individuals.

The main goal of the manuscript "is not to critique conventional modeling approaches per se, but rather to demonstrate how item heterogeneity can be explicitly modeled and how it influences estimates of within-person affect dynamics in ILD data." (see page 11 of the manuscript).

In the revised manuscript, we stress that we do not advice against the use of composite scores in general.

• Comparability and granularity: In addition, if each affect terms are to be treated as a different experience, cross-study comparability may become even more difficult. Should we not then draw any definitive conclusions about negative affect in the literature?

Not necessarily. Some affect scales (e.g. Wilhelm & Schoebi, 2007) have been designed in such a way that a common factor model is likely to hold. Furthermore, even if affect items measure qualitatively different affect states, it remains an empirical question how past findings would differ if researchers had accounted for item heterogeneity. More generally, however, we agree that items in the PANAS (e.g., angry and sad) often differ qualitatively from another. Our study offers a modelling strategy that can be used to investigate whether items differ strongly within individuals, and whether some individuals differentiate less between different affective states than others. The question of how to measure and quantify affect granularity is an increasingly relevant topic. We hope that our modelling approach will aid in this regard.

Also, based on the author's cited resource, "The median number of items used was 8, with a minimum of 1 and a maximum of 52" (https://osf.io/7qtye). Isn't that level of granularity quite extreme? How many items can the model handle without sacrificing

power? While granularity may be informative, the aggregation is also helpful particularly if we're not talking about more "basic" emotions (like sadness, anger), but rather general affective tendencies (more mood like items). Is this approach necessary if the affect items are not too heterogeneous? Or if there is also aggregation at the activation level as well as valence?

Thank you. We agree with the reviewer that there is a trade-off between the level of granularity — i.e. the amount of information and model complexity — and statistical power. Our modelling approach is related to idiographic dynamic network and VAR models, so similar sample size recommendations will apply. We would not recommend including 52 items in any dynamic structural equation model. Instead, researchers could use homogeneous affect scales which are designed in a way that a common within-person factor is likely to hold (e.g. Wilhelm & Schoebi, 2007) or aggregate uni-dimensional items for instance at the activation and valence levels. That is, of course it is possible to aggregate items or use a common-factor model, provided that the assumptions inherent to these approaches are met. We stress this in the revised manuscript.

If researchers are aiming for broad content validity and have good reasons to use heterogenous affect items, we would recommend using items that assess the far ends of the affective poles (e.g. depression, anger, stress), using two items for each pole. Of course, our modeling approach could be used to identify items in a scale's developmental stage with high or low item specificity. If item specificity (heterogeneity) is low in an empirical application, it would be reasonable to use a DSEM with a common within-person factor, that is, a simpler model. For a study with 52 items, it is reasonable to assume that there are several (sets of) items that measure a common factor, i.e., there are items with low item specificity within a set of items, such that dimension reduction by use of factor models or composite scores may be reasonable. That said, collecting 52 items and assuming they all load onto a common affect factor is highly unusual for EMA as well as panel studies.

We now discuss that model D is limited in the number of items that can be modeled simultaneously in the discussion section. Note, however, that EMA studies do usually not collect a large number of items per time point due to time constraints and high participant burden.

Also, the problem seems to origin at the research design part where researchers are deciding on items at random without proper psychometric work to validate the affect measure. So, the lack of comparability issue is more to do with the standardization of affect measure. The fix proposed by this paper at the modeling/analytical part may not fully address all the concerns that the authors raised and therefore could warrantee some attentions.
It would be helpful if the authors calibrated their claims with similar considerations.

We agree with the reviewer. A general and closely related problem is that there is no common standard for how to measure affect and that work on the validation of affective EMA measures is lacking. Especially in ILD studies, researchers rely on different scales and

item sets, which are often adapted without proper psychometric validation. One potential approach is to enhance (or even enforce) standardization in EMA studies and advance a validation process for items used in EMA assessments. Some recent initiatives are already contributing to this line of work (e.g., https://journals.sagepub.com/doi/10.1177/25152459241286877; https://doi.org/10.1037/pas0001200; https://link.springer.com/article/10.3758/s13428-025-02626-1; https://link.springer.com/article/10.1007/s11136-024-03678-0). Although we agree that standardization offers many advantages, such as improved comparability across studies, it also comes with costs. There may be valid reasons to add, replace, or revise items in EMA protocols as our theoretical understanding evolves or as studies address different research questions and contexts. Enforced standardization would limit this necessary flexibility and could hinder theoretical and methodological progress. For these situations, appropriate modeling techniques are needed. Standardization also does not guarantee that the psychometrically most appropriate items are selected. Researchers may choose to use the PANAS as a standardized measure of affect. In that case, the issue of item heterogeneity will not be solved, although comparability across studies will have increased.

We now discuss the possibility and initiatives for using standardized EMA items and to improve validation and explore item performance in affective research in the discussion section.

• Model requirements: Clearer guidelines on the required number of participants, observations, and items would strengthen the paper. Since each affect item is treated as a separate construct, how many items can be included before the model becomes too complex? Perhaps also a guideline for when the model is appropriate vs sticking to aggregation. That may help expand the model’s usefulness beyond just affect measures.

We now cite simulation studies investigating the statistical performance and minimum sample size requirements of DSEM, which also apply to our modelling approach. Bearing in mind that the model that we propose estimates less parameters than an unrestricted VAR model (i.e., a VAR model including all AR and CL effects as well as simultaneous innovation correlations), it should be able to handle as many items as a VAR model. However, note that the main factor influencing the sample size requirements is not the number of items, but whether person-specific parameters (random effects) are required, since this substantially increases the number of estimated parameters.

We also understand the reviewers wish for guidelines when aggregation may be acceptable. However, aggregation versus no-aggregations is not a statistical question, but a psychometrical one. We do not want to advise researchers to use composite scores with an unclear and ambiguous meaning because they sampled enough data points.

To provide a guideline, as also suggested by reviewer 1, we now include a table and provide a list of recommendations for best practice, focusing on item selection and statistical modelling.

• Support for claims: Some of the claims could be further supported by evidence or explained more. Some examples below:

o One of the main demonstrations of item heterogeneity comes from here: “If the correlations between item-specific AR parameters and innovation variances are substantially below one (or below the value that we expect based on the parameters), this indicates that different items capture distinct feelings with unique temporal dynamics and situational sensitivities and thereby provide evidence for item heterogeneity in within-person dynamics over time.” This reasoning could be explained more clearly. Specifically, why does this metric operationalize item heterogeneity?

Our argument is based on the idea that if all items reflect the same underlying affective dimension, they should show the same temporal dynamics. If one instead of multiple constructs are measured at the within level, then the AR parameters and innovation variances of different affect items should follow a similar pattern. Therefore, under item homogeneity, a strong correlation between these parameters is expected.

However, when this correlation is substantially lower than expected, it suggests that items differ in their temporal persistence and sensitivity to moment-to-moment fluctuations. This divergence suggests that the items are not driven by a single underlying process but instead capture distinct emotional experiences with their own temporal signature or unique within-person dynamics (experiencing anger over time is different from experiencing sadness over time). Therefore, we interpret weak associations between AR parameters and innovation variances as evidence of item heterogeneity. Note that low correlations between the AR (and/or innovation variances) is only an indication of item heterogeneity; it is not a statistical test.

Another indicator of item heterogeneity which we discuss in the article is if the factor loadings in DSEM model with a common (one-dimensional) within factor vary considerably across individuals. This suggests that weak measurement invariance is violated and that the psychometric meaning of the affect factor differs across person. The third support for item heterogeneity is given by explicitly modeling it and quantifying it in terms of variance coefficients.

Unfortunately, there are not established fit measures for DSEM models (akin to traditional CFA or SEM models). If this were the case, researchers could directly test whether a single-factor (approximately) holds at the within level.

o There are major concerns about measurement error and reliability issues with the suggestion to dilute all the affect items into single items measuring separate constructs. The authors do discuss this issue in their paper, but it would be helpful if they elaborated on them further with some single item reliability research or additional support to back their claims. I also had specific questions about claim below:
“The problems arising from the low reliability of single items may be circumvented by collecting data on several parallel items per emotion facet or feeling. That is, researchers may, for instance, measure momentary anger with two items that capture the current anger level of a person.” Again, this to me is indicating that each of the affect items should be treated as

a different construct? That makes our affect constructs go from generally 2 (which may be too little) to 50+. Also, the suggestion to add more items does not seem very practical when survey length is such an important consideration in EMA research.

We appreciate the reviewer's concerns regarding measurement error, reliability, and the feasibility of treating affect items as specific emotional facets. We agree that single-item measures can have limited reliability.

At the same time, we believe there may be a misunderstanding. We are not suggesting that EMA studies should measure 50+ distinct emotion constructs or that each affect item represents an entirely separate construct. In practice, most EMA studies include far fewer items (than 50+) to assess momentary affect. Moreover, even if a study were to include a large number of affect items, it is highly unlikely that they fit a common factor model. In such cases, a multidimensional structure would be required to appropriately model the data. Simply aggregating all items into a single score ignores and camouflages the multidimensionality and makes the resulting score difficult to interpret. It is quite interesting that in many areas of psychology, researchers usually prefer multidimensional measurement models (e.g., bifactor and related models) and reject the single-factor models because of its strict assumptions, whereas the opposite is true for affect measurements.

In the EMOTE database we analyzed, researchers typically selected few (3-6) items to measure different facets of positive or negative affect. Some of these items already form coherent clusters (e.g., *sad* and *depressed*), and in such situations it is feasible—and psychometrically advisable—to include a small number of parallel items so that other facets (e.g., anger, anxiety) are assessed with comparable reliability. This does not imply creating dozens of new constructs. Instead, it means strengthening the measurement of the specific facets that researchers already intend to study.

Our modeling approach is compatible with this level of measurement refinement and can accommodate small sets of items per emotional facet without placing undue burden on participants or compromising EMA feasibility.

Alternatively, researchers can use validated and highly reliable multidimensional affect scales, such as the MDBF by Steyer et al. (1997) or its short version by Wilhelm and Schoebi (2007). These instruments were explicitly developed to adhere to a clear psychometric measurement model and provide reliable assessment across multiple affective dimensions. Using such scales offers a practical way to ensure psychometric quality in EMA studies without substantially increasing participant burden.

• Figures: I would recommend creating two wordclouds: separate for negative and positive affect items. The figure is less effective representation of how affect is measured when it is not shown separate.
• Clarity of terminology: Some of the terms are not defined when introduced (e.g. random innovation variance) and that may limit the accessibility of the article to a newer audience.
• Code: I expected there to be a code output or tutorial for readers to use in the main paper Appendix. The code is linked in the OSF folder I can see

but it would be helpful if the code was explained in the Appendix in the paper.
• This is not necessary to cite but other papers have looked at item performance issues that may also be useful to consider in the paper: Siepe, B. S., Rieble, C. L., Tutunji, R., Rimpler, A., März, J., Proppert, R. K., & Fried, E. I. (2025). Understanding Ecological-Momentary-Assessment Data: A Tutorial on Exploring Item Performance in Ecological-Momentary-Assessment Data. Advances in Methods and Practices in Psychological Science, 8(1), 25152459241286877.

Thank you for these suggestions. We created separate word clouds for positive and negative affect, clarified terminology when first introduced, and expanded the code annotations on OSF. We also appreciate the recommended reference and cite it in the revised manuscript.

Overall, even with these concerns, I like the paper and think it will be a valuable addition to the field.

Thank you for your feedback and valuable suggestions to improve the manuscript.

Reviewer #4 (Remarks to the Author):

The manuscript addresses a highly relevant topic. In intensive longitudinal data (ILD) research, there are indeed many reasons why researchers employ different emotion terms to operationalize their constructs. I am therefore very pleased to see another contribution that directly engages with this issue. The introduction is very clearly written, the discussion of item heterogeneity is well-structured, and the purpose of the study comes across clearly. That said, I have several points of attention that I believe would strengthen the manuscript if it is considered for publication:

Thank you for your overall positive evaluation and constructive feedback.

Levels of item heterogeneity
The paper introduces three different forms of item heterogeneity, all of which are conceptualized as between-person differences (item specific components for each individual). This framing emphasizes person-specific variance, and the authors highlight inter-individual differences in item parameters that can be captured by the model. However, Brose et al. (2020) also emphasize the role of context, noting that “heterogeneity in content in interaction with situational characteristics prevents common covariation.” While the authors briefly mention that multimethod modeling can incorporate time-invariant or time-varying predictors to explain heterogeneity, there is little elaboration on this point. Since the model includes contemporaneous effects, it would be helpful to clarify whether and how contextual variables (e.g., events, stressors) could be specified in the model to account for item-specific variance under different conditions. An example analysis would also be highly appreciated.

Indeed, this would be a fascinating extension of our model, as it focuses on explaining heterogeneity using time-varying contextual variables. However, we believe it is beyond the scope of the current manuscript – first of all, because introducing a new and complex topic requires sufficient space for discussion; second, because the implementation of such a model is not (yet) possible in Mplus.

There are multiple ways to incorporate contextual variables or model contextual effects in this context:

One approach is to incorporate time-varying contextual variables, such as time-specific events or stressors, and model the interaction effects between these variables and contemporaneous effects, dynamic processes, or innovation variances. A recent article shows how to include such within-level interaction effects in multilevel time series models in Stan (see Koslowski & Holtmann, 2025). Similar to R2 in multilevel models, researchers could compute the relative reduction in the contemporaneous, dynamic, and innovation item-heterogeneity coefficients before and after the inclusion of the interaction terms. The approach suggested by Koslowski & Holtmann (2025) has recently been integrated into the R-package mlts (see Koslowski et al., 2025). However, the model they propose and the approach implemented in mlts is a traditional DSEM, not a Residual DSEM. The package does not cover RDSEM or random loading parameters. As mentioned above, Mplus does not allow for modeling within-level interaction effects.

Another possibility is to include a contrast matrix at the within-person level, similar to repeated ANOVA designs. In this case, it would be advantageous to predict each (affect) item on the contrast matrix and model auto- and cross-lagged effects based on the residuals of the items (after controlling for the contrasts). This way, researchers are able to investigate whether contextual variables have differential effects on affective states (represented by each item) and how the affective states are interrelated after controlling for contextual variables. Also this approach represents a complex model extension.

Note that explaining reference and non-reference item-specific effects requires special treatment and care. Overall, explaining item-specific effects at the within-person level is not a trivial extension and beyond the scope of the present study.

Holtmann, J., Koslowski, K. Modeling within-level latent interaction effects in multilevel vector-autoregressive models. *Behav Res* **57**, 277 (2025). https://doi.org/10.3758/s13428-025-02694-3

Between person model
The authors provide information and a figure on the within person relations. What is the structure of the between person model? Would it be feasible to also add this as a figure to the manuscript for completeness?

The between-person model corresponds to an unrestricted variance-covariance matrix of all random effects (i.e., random item intercepts, individual-specific regression and AR effects, innovation variances) as well as potential between-level covariates. As a Figure might take up quite some space but convey little additive information, we have decided to refine the description of the between-person model in the text instead (see revised manuscript).

Clarification of the jingle–jangle fallacy
The authors mention this concept briefly. It may be useful to explicitly define what is meant here, as not all readers will be familiar with the distinction between "jingle" and "jangle" problems.

Thank you. We have added an explanation of the fallacies in the revised manuscript. We would also like to refer the reviewer to our response to an earlier comment by reviewer 1, who had the same question.

Reference items and comparability
The use of reference items is a valuable strategy to create comparability across studies. At the same time, the limitations of this approach in ILD research deserve more discussion. For example, on p.14 the authors state: "As long as the same reference item is used, momentary affect is measured in a comparable manner across studies." Yet comparability in ILD studies is not only constrained by reference items but also by major design differences (e.g., sampling frequency, study length, participant instructions). Is the comparability still given even under different study designs?

We fully agree with the reviewer that there are many aspects that may enhance or compromise comparability in ILD study. We focus on *psychometric* comparability (i.e., the psychometric meaning of the latent affect factor). By choosing reference items, we ensure that the affect factor is measured in the same way across studies. In the revised version, we clarify that other aspects (such as sampling frequency, study length, participant instructions) are also important to facilitate comparability and refer to psychometric comparability in the revised version. Thank you for pointing this out.

Also see our answer to reviewer 1 above, with respect to the correction for different time intervals between measurement across studies (resulting from, e.g., different sampling frequencies).

Presentation of models and results
The manuscript presents four models: single items, composite score, common factor, and the novel item-specific approach. However, models A and C are already analyzed and results are reported earlier in the text, before the main analysis section. This disrupts the central aim of comparing the four models side by side. It breaks the outline of the manuscript and does not aid the readability. I would recommend restructuring the results section to present all four models systematically in one place, allowing for a more direct and comprehensive comparison.

Thank you. The main goal is to introduce a modeling approach that enables researchers to explicitly examine item heterogeneity at the within-person level and guarantee psychometric comparability when item sets partially overlap across studies. Hence, the article's main focus is on *how* to model item heterogeneity (i.e., Model D).

Models A and C, which use single items and a common factor with random loadings, respectively, are used to provide evidence of item heterogeneity. The goal is not primarily to compare the results of all models with Model D; in fact, comparing all four models is difficult as they capture different aspects and illustrate the presence of different types of item heterogeneity. We do now clarify these points in more detail. In the methods section, we now explain which model is used for which purpose. Furthermore, we have followed your (and reviewer 1's) advice to re-structure the introduction and results section. We now also present the results of models A and C in the results section.

Additionally, associations with outcomes are not reported for models B and C, which leaves the comparison incomplete. I would recommend that there are comparable analyses for the other models so that there is a clear structure that the reader can follow up on after the expectations they are given by the aim of the study.

Associations for the composite score model (Model B) are presented in Figure 5 along with those of Model A. We have now also added the respective associations with the outcomes for the factor model (model C) in Figure 5. We believe that the results and information presented in the manuscript is already quite dense and that adding more figures and results would render the manuscript less readable. Therefore, we have not added an analogous figure to Figure 5 for Model D. In essence, the results in Figure 5 should illustrate that assuming a common AR and innovation variance across affect items would mask that different items' dynamics show different associations with outcomes.

Conceptual comparison of models
Models B and C rely on latent variables or composite scores, while models A and D operate at the level of single items. This makes the comparisons somewhat uneven. Since the novel model (D) uses single items as indicators, it seems important to also compare it directly to the single-item alternative that does not account for heterogeneity. Otherwise, the reader may struggle to understand how the new model differs from, or improves upon, existing approaches. Similarly, it remains unclear how the proposed model is conceptually comparable to a composite score or common factor model, and why researchers should prefer it in practice. A more explicit articulation of the applications and implications of each approach would be very helpful.

Thank you. Again, we do not recommend fitting models B or C if item heterogeneity exists. We recommend fitting only Model D, which assumes a multidimensional factor structure. This relaxes the assumption that the affect items measure the same (or one common) underlying dynamic process. Model C is a one-dimensional model. In case of person-invariant loadings, the model assumes that the same underlying dynamic process is measured by all affect items. It is also assumed that adding or removing items will not distort the measurement of the underlying within-person dynamic process (i.e., the items are treated as interchangeable). Model B would be psychometrically appropriate if Model C holds and certain restrictions can be placed on the loadings and error variances (i.e., tau-parallel measurement structure). Model A is similar to Model D but does not rely on reference items. It is a simplified version of a VAR model. In the discussion section, we discuss differences

between model D and a fully-crossed VAR model and give recommendations on when to prefer which of these models.

Implications of item differences for outcomes
The analyses relating the models to depression are an important step. They show that items differ in their associations with depression, but what does this imply? Do we need to use different items for different people, or is there one item that is best across studies? A more explicit discussion of these implications would strengthen the paper considerably.

One implication of our study is that researchers using heterogeneous and non-interchangeable affect items may observe differential effects on outcome variables that could go undetected if they use composite scores or model a common within-level factor. This is directly relevant for studies that investigate the predictive value of individual affect dynamics for psychopathology or well-being outcomes (see, e.g., https://www.nature.com/articles/s41562-019-0555-0).
Models that rely on composite scores or that model a common within factor are not well-suited for multidimensional (or multifaceted) constructs. One option is to use homogeneous affect scales. Alternatively, researchers may embrace item heterogeneity and use our (or alternative) modeling approach to explicitly model item heterogeneity. We would not like to overinterpret our findings by implying that researchers must use different items for different people or that there is "gold" standard method for measuring affect (based on our findings). Our method enables researchers to examine whether people react (respond) differently to the items they have administered and make findings psychometrically comparable.

Depression outcome
A more general point for reflection: the outcome of depression is itself measured through a set of items, each of which may capture distinct facets or contexts of the construct. In other words, the same heterogeneity problem addressed in this manuscript also applies to many widely used psychological scales (Fried, 2017). While this issue may not be fully resolvable in most research designs, explicitly acknowledging this broader limitation would help situate the current contribution more clearly within the larger measurement and validity literature.
Fried E. I. (2017). The 52 symptoms of major depression: Lack of content overlap among seven common depression scales. Journal of affective disorders, 208, 191–197. https://doi.org/10.1016/j.jad.2016.10.019

We agree with the reviewer. If the outcome is a multidimensional construct assessed by multiple items that load onto different subdimensions, then a multidimensional model or a more granular measurement may be necessary. For baseline outcomes, researchers can easily test the assumption of a unidimensional measurement structure. However, researchers typically do not test this assumption with regard to affect items in ESM designs. Furthermore, when measuring affect using different affect states (e.g., anger, depression, stress, or anxiety), we implicitly assume that all of these states are potentially present in a person simultaneously. This assumption is not made for global/retrospective measures. For example, people may report insomnia and reduced appetite over two weeks without implying that these occur at the same time. Additionally, for a baseline or follow-up

depression measure, heterogeneity in the dynamics across time are not relevant, while these are the main focus of the current study.

We do now discuss the point you raised in the manuscript and point readers to the respective literature.

Additional comments to Results/Discussion
The discussion section remains quite abstract in terms of what the proposed model adds to the existing toolbox and how it could be implemented in practice. At present, readers may struggle to see the concrete value and applicability of the model beyond the technical demonstration. The results section also leaves room to expand on, for example, what types of research questions it would be most useful for, and how it compares to currently established practices. Adding such depth would considerably strengthen the contribution of the paper.

Thank you. We revised and extended the discussion accordingly.

Overall, this manuscript raises an important and timely issue for the ILD field and provides a valuable contribution. However, in its current form, the structure of the manuscript does not fully meet the expectations set by its stated aims. With more clarity on the model comparisons particularly in terms of presenting and comparing the models, and a deeper discussion of implications and limitations, the paper will provide a comprehensive contribution to the literature.

Thank you for your valuable feedback. We now discuss the implications and limitations of the proposed model in greater detail.

Dear Dr. Schiffer,

Thank you very much for your feedback. In the following, we address the reviewers concerns and provide information on which changes we have undertaken in response to these concerns and where to find these changes in the manuscript. The reviews are printed in black, our responses are printed in blue.

EDITORIAL REQUESTS:

The manuscript currently contains references to results that are deposited on external repositories. All referenced results from the analyses supporting your interpretation of the data need to be included in the main manuscript or a Supplementary Information file.

The reviewers ask for the inclusion of some additional explanations. As you address these concerns, please keep in mind that the peer review record will be published alongside your paper, so comprehensive repetitions of your rebutally comments will not be necessary in cases where appropriate changes have been undertaken in the revised paper already.

The Introduction section is excessively long and should be revised to focus on key aspects necessary to understand the motivation behind the work and the background literature. Repetitions can be removed and extensive explanations can be reduced or may be more suitable for the Discussion.

Please review our specific editorial comments and requests regarding your manuscript in the attached "Editorial Requests Table". Please outline your response to each request in the right hand column. Please upload the completed table with your manuscript files as a Related Manuscript file.

If you have any questions or concerns about any of our requests, please do not hesitate to contact me.

SUBMISSION INFORMATION:

In order to accept your paper, we require the files listed here https://www.nature.com/documents/commsj-file-checklist.pdf .

OPEN ACCESS:

Communications Psychology is a fully open access journal. Articles are made freely accessible on publication. For further information about article processing charges, open access funding, and advice and support from Nature Research, please visit https://www.nature.com/commspsychol/open-access

At acceptance, you will be provided with instructions for completing the open access licence agreement on behalf of all authors. This grants us the necessary permissions to publish your paper. Additionally, you will be asked to declare that all required third party permissions have been obtained, and to provide billing information in order to pay the article-processing charge (APC).

* TRANSPARENT PEER REVIEW: Communications Psychology uses a transparent peer review system. On author request, confidential information and data can be removed from the published reviewer reports and rebuttal letters prior to publication. If you are concerned about the release of confidential data, please let us know specifically what information you would like to have removed. Please note that we cannot incorporate redactions for any other reasons.

* CODE AVAILABILITY: All Communications Psychology manuscripts must include a section titled "Code Availability" at the end of the methods section. We require that the custom analysis code supporting your conclusions is made available in a publicly accessible repository at this stage; please choose a repository that generates a digital object identifier (DOI) for the code; the link to the repository and the DOI must be included in the Code Availability statement. Publication as Supplementary Information will not suffice.

* DATA AVAILABILITY:
All Communications Psychology manuscripts must include a section titled "Data Availability" at the end of the Methods section. More information on this policy, is available in the Editorial Requests Table and at http://www.nature.com/authors/policies/data/data-availability-statements-data-citations.pdf.

Please use the following link to submit the above items:
https://mts-commspsychol.nature.com/cgi-bin/main.plex?el=A7DU3lW1B2Fhh1I7A9ftde7OxKRNcItd10Obl5Rm6gZ
** This url links to your confidential home page and associated information about manuscripts you may have submitted or be reviewing for us. If you wish to forward this email to co-authors, please delete the link to your homepage first **

We hope to hear from you within two weeks; please let us know if you need more time.

Best regards,

Marike

Marike Schiffer, PhD
Chief Editor
Communications Psychology

REVIEWERS' COMMENTS:

Reviewer #1 (Remarks to the Author):

Thank you again for the opportunity to review Manuscript COMMSPSYCHOL-25-0477, titled "Improving the Comparability of Momentary Affect Assessments: The Role of Item-Specific Effects in Within-Person Affect Dynamics." We consider the presentation of the article and its accessibility to a wider readership to be considerably improved, and we continue to believe that this work will be an important contribution to the methodological literature in this area.

Thank you for the overall positive evaluation of our manuscript.

The authors' rebuttals are thoughtful, knowledgeable, and responsive feedback to our concerns. It is not clear, however, that these concerns have been thoroughly addressed in the body of the manuscript itself. Indeed, without specific callouts to pages or line numbers in the revised manuscript (with a few exceptions), we [this reviewer and the reviewer-in-training] spent additional time looking for (and consulting with each other regarding) the relevant information in the manuscript which was satisfactorily addressed in the letter. We have identified some points from their rebuttal that we do not see in the body of the manuscript, but that we determined would be important for all readers.

Thank you for this helpful comment. We now indicate explicitly in our response letter where the relevant information can be found in the revised manuscript.

The longer responses from the authors' rebuttal might be worth including as footnotes (if permitted by the journal) or perhaps as a supplement, without these we expect that a naive reader may be as confused or unconvinced on some of these points as the reviewers were on the first submission.

Thank you for this suggestion. Unfortunately, Communications Psychology does not permit footnotes. We also do not think it is necessary to move these longer responses into a supplement, because the journal uses a transparent peer review system. Readers will therefore be able to access our full responses to the reviewers' earlier comments directly through the peer review record.

Responses to our Major points:

1. This is a clear and thoughtful explanation. The point that this modeling approach "allows researchers to model heterogeneity across individuals (and items)" is especially important for readers evaluating their "usual" approach and considering following the recommendations. It's not clear that this is explained in the text, however, but it would be a helpful addition.

We added the following sentence on page 4 in the paragraph starting with "The objectives of this study are threefold."…

"Second, we introduce a modeling approach that explicitly accounts for item heterogeneity at both the within-person and between-person levels within a Dynamic Structural Equation Modeling (DSEM) framework. **Because the item-specific parameters are modeled as random (i.e., person-specific) effects, our approach allows researchers to model heterogeneity both across items and across individuals**."

a. Another important point to consider adding to the main text: "our approach requires that at least one item (i.e., the chosen reference item) is invariant across individuals. This assumption would be violated if individuals interpret this item in fundamentally different ways." Again, this clear statement of the requirements and of the assumptions of this new approach would be very helpful, especially given the authors' critique of the typical approach and disregard for the assumptions which underlie it.

2. Thank you for this explanation. It is not clear that the recommendation for a core/common set of affect items is in the manuscript. If a goal of this manuscript is not simply a demonstration of the issues with composite scales, but instead directing applied researchers to alternatives then clear steps and rationale should be provided and organized in an easily findable manner.

We now explicitly state the sentence in **Section 4.6 Comparability of Results (page 22):**

"In contrast, the reference-item modeling approach improves the comparability of findings as long as the same reference item is used. For example, the concurrent regressions between the reference item (e.g., sad) and a non-reference item (e.g., stressed) should remain invariant, even if other items (e.g., anxious, angry) are added to or removed from the model. **Note that our approach requires at least one item, namely, the chosen reference item, to be invariant across individuals. This assumption would be violated if individuals interpret that item in fundamentally different ways. This assumption is not unique to our approach but rather reflects a broader issue in latent variable modeling when evaluating measurement invariance, because latent variable models always require researchers to fix the scale of the latent variable."**

3. Thank you for this explanation, and the text on p. 26 is helpful in communicating this. The directive in response to this question makes the proposed approach much easier to follow.

Ok, thank you.

4. Thank you for your responsiveness, the added framing on pp. 4-5 is especially useful in clarifying the goals of the manuscript. Table 4 is also helpful, and if it is possible to move it earlier in the paper, this may make it even more so (we leave this to the author and editor judgment).

Thank you for this helpful suggestion. We considered moving Table 4 earlier in the manuscript. However, because Table 4 is intended as a synthesis of the preceding discussion of the different forms of item heterogeneity, their implications, and recommendations for modeling practice, we felt that its current placement best preserves the conceptual flow of the paper. Moving it substantially earlier would require readers to interpret the summary before the relevant distinctions have been fully introduced. We therefore retained its current position.

a. That said, why is there no text in the first “Implications when ignored” cell of Table 4? Although the answer may seem obvious, or perhaps irrelevant because researchers using intensive longitudinal data do not often ignore person means in their analyses, but for completeness it is important to articulate.

Thank you. We now added the consequences of not modeling indicator-specific traits.

“The model will be too restrictive and likely not fit the data. If a common trait and measurement errors are estimated at level 2, only i-1 measurement errors should be modeled.”

Responses to our Minor points:
1. Thank you for adding the explanation to the text.
2. We appreciate the restructuring and agree that these sections are much clearer.
3. We appreciate the additions on p. 16 to address this question.
4. We appreciate the added sentence in the Method section.
5. We appreciate the additions on p. 6 and p. 32 to address this question.

We have one other observation from another reviewer’s comments:

Under reviewer 3, comment beginning with “Also, based on the author’s cited resource…”

The rebuttal notes that “we would recommend using items that assess the far ends of the affective poles (e.g. depression, anger, stress), using two items for each pole.” This strikes us as helpful advice, but we do not see it in the body of the manuscript. The authors may consider adding this.

Thank you. We now include the following paragraph in the Discussion:

“It is important to recognize the inherent trade-off between measurement granularity and statistical power. As in related idiographic dynamic network and VAR models, including a large number of heterogeneous affect items increases model complexity and the corresponding data requirements. When researchers expect low item specificity, more parsimonious approaches, such as homogeneous affect scales, common-factor models, or composite scores at the level of activation and valence, may be preferable, provided their assumptions are met. When broader content coverage is desired, we recommend selecting items that capture the far ends of the

major affective poles (e.g., depression, anger, stress), using two items for each pole. More generally, our approach may help determine when item-level modeling is justified and when dimension reduction is the more appropriate strategy."

Many of the considerations that we found important would be useful in designing future studies. It may be worth adding a paragraph to the Discussion that includes takeaways, both for researchers analyzing secondary data (likely designed and powered for different research questions) and for researchers designing studies specifically focused on affect-related hypotheses. It is our assessment that the manuscript, if published, would be much more impactful with this inclusion and organization.

Thank you for this suggestion. We agree that such guidance could be valuable. However, it is not entirely clear which specific considerations the reviewer would like us to add to the Discussion. Our previous response addressed a broad range of points, and it would not be feasible to incorporate all of them into the manuscript within the journal's page limits. A more extensive treatment of these issues would likely be better suited to a dedicated tutorial or applied companion paper rather than the present methods-focused article (see also comment by Reviewer #4).

Reviewer #2 (Remarks to the Author):

I co-reviewed this manuscript with one of the reviewers who provided the listed reports. This is part of the Communications Psychology initiative to facilitate training in peer review and to provide appropriate recognition for Early Career Researchers who co-review manuscripts.

Reviewer #4 (Remarks to the Author):

Thank you for the elaborate revision! I believe the authors have addressed the comments well and the manuscript is much more structured and goal directed. I am excited that this topic is getting attention and that solutions are developed. In the future I hope that such a topic could be addressed more straightforward and more applied, so that the burden of taking such analyses up could be reduced for researchers. I hope to see an application or tutorial with more examples for substantive researchers in the future.
Thank you for your positive and helpful comments.

Some minor things to be addressed:
p. 5 "In the context of intensive longitudinal data analysis, at least three distinct forms of item heterogeneity can be studied."

As addressed earlier the item heterogeneity dealt with there is only on the between person level. I would therefore consider adding the remark that the three forms discussed are between person level differences.

Thank you for this helpful suggestion. We now clarify in the manuscript that the three forms of item heterogeneity discussed in this section refer to between-person-level differences. We added the following sentence and hope readers won't be too confused by this.

"In the context of intensive longitudinal data analysis, at least three distinct forms of item heterogeneity can be studied. These refer to between-person differences in (1) stable person means, (2) contemporaneous within-person relationships among items, and (3) within-person dynamics over time. **Our proposed modeling approach enables researchers to**

investigate these different types of item heterogeneity by means of between-person differences."

p. 26 correlations are rounded to 3 digits, while in other places they are rounded to two. It would be recommended to use a common approach?

Thank you for noting this inconsistency. We have now harmonized the rounding of correlations throughout the manuscript.

Table 4 leaves very little margins in the cells. The column names are hard to differentiate.

Thank you. We have split Table 4 into two tables to improve readability.

The authors mention they added annotated code on osf? For intersted readers this should be better indicated - as I was not able to find such annotations?

Thank you for pointing this out. We have revised the manuscript to indicate the annotated code on OSF more clearly, so that interested readers can locate it more easily.

In the nature of Mplus it is of course unfortunate that the analysis is not easily reproducible and accessible to all readers. A future tutorial would indeed help this barrier.

We agree that accessibility is an important issue and that a future tutorial could help lower this barrier for readers. We also plan to implement these features in mlts, our R package for estimating multilevel latent time series models. The package already estimates only $i - 1$ measurement error terms in models with common between-level factors, such that indicator specificity with respect to stable person means is handled automatically. Random factor loadings and the residual approach is being implemented in the future, so that the model can also be estimated in R.
