# Supplementary material for: Improving the comparability of momentary affect assessments and accounting for item-specific effects in within-person affect dynamics
Source: Commun Psychol. 2026 Aug 19;4:121. doi: 10.1038/s44271-026-00473-0 (PMC13498562; doi:10.1038/s44271-026-00473-0)
Supplement: Supplementary file 2 [file 44271_2026_473_MOESM2_ESM.pdf]

Supplemental Material to "Improving the Comparability of Momentary Affect Assessments: The Role of Item-Specific Effects in Within-Person Affect Dynamics"

Tobias Koch[1*], Miriam F. Jähne[1], Kenneth Koslowski[2], Peter Koval[3], Tanja Lischetzke[4], Daniel McNeish[5], Jana Holtmann[2]

*Corresponding author(s). E-mail(s): tobias.koch@uni-jena.de;
Contributing authors: miriam.francesca.jaehne@uni-jena.de; kenneth.koslowski@uni-leipzig.de; p.koval@unimelb.edu.au; tanja.lischetzke@rptu.de; dmcneish@asu.edu; jana.holtmann@uni-leipzig.de;

1 Model D and Item specificity coefficients

The basic between-within-level decomposition of the observed variables $y_{k,it}$ in Model D is given in Equation 1 in the main article. The within-level model of Model D is a residual dynamic structural equation model. First, the contemporaneous within-person item associations are modeled via simultaneous regressions. Without loss of generality, one within-person variable is selected as the reference (or anchor) item, e.g., $y^w_{1,it} = \eta^w_{1,it}$. The remaining items $y^w_{k,it}$ for $k \neq 1$ function as non-reference items, which are regressed on the reference item at the same time point t. The simultaneous regression is given by

$$\begin{bmatrix} y^w_{1,it} \\ y^w_{2,it} \\ y^w_{3,it} \\ \vdots \\ y^w_{k,it} \end{bmatrix} = \begin{bmatrix} \beta_{1,i} & 0 & 0 & 0 \\ \beta_{2,i} & 1 & 0 & 0 \\ \beta_{3,i} & 0 & 1 & 0 \\ \vdots & \vdots & \vdots & \vdots \\ \beta_{k,i} & 0 & 0 & 1 \end{bmatrix} \begin{bmatrix} \eta^w_{1,it} \\ \tau^w_{2,it} \\ \tau^w_{3,it} \\ \vdots \\ \tau^w_{k,it} \end{bmatrix} + \begin{bmatrix} \varepsilon_{1,it} \\ \varepsilon_{2,it} \\ \varepsilon_{3,it} \\ \vdots \\ \varepsilon_{k,it} \end{bmatrix}, \tag{1}$$

where $\beta_{1,i}$ for the reference item is fixed to 1. Note that all remaining coefficients $\beta_{k,i}$ for $k \neq 1$ can be freely estimated and are allowed to vary across persons i. The latent variables $\tau^w_{k,it}$ represent residual components, capturing the portion of a non-reference item that is not shared with the reference item. For identification, all measurement errors $\varepsilon_{k,it}$ must be fixed to 0 in Equation 1. When multiple items assess the same affect facet (e.g., sad, unhappy, downhearted), latent factors can be modeled, allowing measurement errors $\varepsilon_{k,it}$ to be freely estimated. This approach is similar to the multirater modeling approach proposed by [**?**].

Next, the dynamics in the reference item variable $\eta^w_{1,it}$, as well as in the item-specific regression residuals of the non-reference items, $\tau^w_{k,it}$, are modeled across time using an AR(1) process:

$$\begin{bmatrix} \eta^w_{1,it} \\ \tau^w_{2,it} \\ \tau^w_{3,it} \\ \vdots \\ \tau^w_{k,it} \end{bmatrix} = \begin{bmatrix} \phi_{1,i} & 0 & 0 & 0 & 0 \\ 0 & \phi_{2,i} & 0 & 0 & 0 \\ 0 & 0 & \phi_{3,i} & 0 & 0 \\ \vdots & \vdots & \vdots & \ddots & \vdots \\ 0 & 0 & 0 & 0 & \phi_{k,i} \end{bmatrix} \begin{bmatrix} \eta^w_{1,i(t-1)} \\ \tau^w_{2,i(t-1)} \\ \tau^w_{3,i(t-1)} \\ \vdots \\ \tau^w_{k,i(t-1)} \end{bmatrix} + \begin{bmatrix} \zeta_{1,it} \\ \zeta_{2,it} \\ \zeta_{3,it} \\ \vdots \\ \zeta_{k,it} \end{bmatrix}, \tag{2}$$

with $\phi_{k,i}$ denoting the AR effects and $\zeta_{k,it}$ the dynamic residuals (i.e., innovations). While it is possible to include cross-lagged effects among the non-reference item-specific effects $\tau^w_{k,it}$, doing so may lead to results that vary depending on which non-reference items are included or excluded across studies.

At the between-person level, all random effects are decomposed into a fixed effect $\kappa_{0,\bullet}$ and a person-specific deviation $\nu_{\bullet,i}$, where the vector of $\nu_{\bullet,i}$ follows a multivariate normal distribution, $\text{MVN}(0, \boldsymbol{\Omega})$, and $\boldsymbol{\Omega}$ is the variance-covariance matrix of the random effects.

$$
\begin{bmatrix} \mu_{1,i} \\ \mu_{2,i} \\ \mu_{3,i} \\ \mu_{k,i} \\ \beta_{1,i} \\ \beta_{2,i} \\ \beta_{3,i} \\ \vdots \\ \beta_{k,i} \\ \phi_{1,i} \\ \phi_{2,i} \\ \phi_{3,i} \\ \vdots \\ \phi_{k,i} \\ \ln(\sigma^2_{\zeta_{1,i}}) \\ \ln(\sigma^2_{\zeta_{2,i}}) \\ \ln(\sigma^2_{\zeta_{3,i}}) \\ \vdots \\ \ln(\sigma^2_{\zeta_{k,i}}) \end{bmatrix} = \begin{bmatrix} \kappa_{0,\mu_1} \\ \kappa_{0,\mu_2} \\ \kappa_{0,\mu_3} \\ \kappa_{0,\mu_k} \\ \kappa_{0,\beta_1} \\ \kappa_{0,\beta_2} \\ \kappa_{0,\beta_3} \\ \vdots \\ \kappa_{0,\beta_k} \\ \kappa_{0,\phi_1} \\ \kappa_{0,\phi_2} \\ \kappa_{0,\phi_3} \\ \vdots \\ \kappa_{0,\phi_k} \\ \kappa_{0,\ln(\sigma^2_{\zeta_1})} \\ \kappa_{0,\ln(\sigma^2_{\zeta_2})} \\ \kappa_{0,\ln(\sigma^2_{\zeta_3})} \\ \vdots \\ \kappa_{0,\ln(\sigma^2_{\zeta_k})} \end{bmatrix} + \begin{bmatrix} \nu_{\mu_{1,i}} \\ \nu_{\mu_{2,i}} \\ \nu_{\mu_{3,i}} \\ \nu_{\mu_{k,i}} \\ \nu_{\beta_{1,i}} \\ \nu_{\beta_{2,i}} \\ \nu_{\beta_{3,i}} \\ \vdots \\ \nu_{\beta_{k,i}} \\ \nu_{\phi_{1,i}} \\ \nu_{\phi_{2,i}} \\ \nu_{\phi_{3,i}} \\ \vdots \\ \nu_{\phi_{k,i}} \\ \nu_{\ln(\sigma^2_{\zeta_1}),i} \\ \nu_{\ln(\sigma^2_{\zeta_2}),i} \\ \nu_{\ln(\sigma^2_{\zeta_3}),i} \\ \vdots \\ \nu_{\ln(\sigma^2_{\zeta_k}),i} \end{bmatrix}, \tag{3}
$$

The person-specific item-specificity coefficients can be computed based on the contemporaneous effects $\beta_{k,i}$ (i.e., the association between the reference and a non-reference item at a given time point t), the lagged effects (i.e., the association between the reference and a non-reference item across time), and the innovation components (i.e., the association between the reference and a non-reference item at time t, after accounting for lagged effects). In the following, we present the formulas for computing the item-specificity coefficients when using within-person standardized results (abbreviated *std.* in the following), which are readily available in Mplus [**?**].

Contemporaneous item specificity is calculated by

$$
ISCon(std.y^w_{jk,i}) = 1 - (std.\beta_{k,i})^2 \tag{4}
$$

Time-lagged item specificity is calculated by

$$
ISLag(y^w_{jk,i}) = 1 - \frac{(std.\beta_{k,i})^2 \cdot (std.\phi_{1,i})^2}{(std.\beta_{k,i})^2 \cdot (std.\phi_{1,i})^2 + (std.\phi_{k,i})^2} \tag{5}
$$

Item specificity for the innovation component is calculated by

$$ISInn(std.y^{w}_{jk,i}) = 1 - \frac{(std.\beta_{k,i})^2 \cdot std.\sigma^2_{\zeta_{1,i}}}{(std.\beta_{k,i})^2 \cdot std.\sigma^2_{\zeta_{1,i}} + std.\sigma^2_{\zeta_{k,i}}} \tag{6}$$

In addition, the within-time point correlations between the innovations of non-reference items can be interpreted as indicators of partial item homogeneity. These correlations reflect the extent to which two non-reference items converge in their assessment of momentary fluctuations in affect, after controlling for the reference item. In Figure 3, these correlations are denoted by $\rho_{kk',i}$. Correspondingly, partial item heterogeneity can be quantified as $1 - (\rho_{kk',i})^2$.

2 Supplementary Tables and Figures

Table S1 Momentary Affect Items Ordered by Number of Studies

Affect Type	Item	Number of Studies
NA	Angry	23
PA	Happy	21
NA	Sad	20
PA	Relaxed	18
NA	Anxious/Afraid	17
NA	Stressed	13
NA	Depressed	9
NA	Guilty	8
NA	Ashamed	8
NA	Nervous	5
PA	Contented	5
NA	Fearful	5
PA	Proud	4
NA	Irritated	4
PA	Confident	4
PA	Cheerful	3
NA	Lonely	3
PA	Calm	3
PA	Excited	3
PA	Satisfied	3
PA	Enthusiastic	2
NA	Disappointed	2
NA	Embarrassed	2
NA	Upset	1
PA	Positive	1
PA	Joyful	1
NA	Scornful	1
PA	Relieved	1
PA	Euphoric	1
PA	Elated	1
PA	Trusting	1
PA	Connected	1
PA	Active	1
NA	Disgusted	1
NA	Annoyed	1
NA	Negative	1
NA	Dull	1
PA	Self-Conscious	1
NA	Sleepy/Tired	1
NA	Sluggish	1
PA	Alert	1
NA	Bored	1

Note: The number of studies indicates how many ESM/EMA studies included the item. Key studies include: Depressive symptoms diary, Negative events diary, FEEL Study 1, Leuven 3-wave longitudinal study, Leuven emotions in daily life 2011, Objectification in women's daily lives (Studies 1-3), Emotional events in daily life, Exam results study, Leuven clinical study, Everyday emotion regulation, Leuven BPD study, Leuven emotion dynamics 2017, Leuven couples study 2014/2016, Leuven emotions in daily life 2008/2012, Secrecy diary, William & Mary emotion diary, ACU emotions in daily life, Trier social stress study, Motivated emotion regulation diary, and Assessing, Understanding, and Regulating Affect (AURA).

Table S2 Between-person correlations between individual-specific parameter estimatess: FEEL study I

Correlation between				95% CI	
Var 1	Var 2	estimate	posterior SD	lower bound	upper bound
sad	AR sad	0.111	0.091	-0.071	0.285
sad	AR stressed	-0.123	0.093	-0.299	0.063
sad	AR angry	0.203	0.091	0.017	0.372
sad	logvar sad	0.015	0.087	-0.156	0.184
sad	logvar stressed	-0.479	0.068	-0.602	-0.335
sad	logvar angry	0.164	0.085	-0.006	0.323
stressed	AR sad	0.134	0.091	-0.049	0.310
stressed	AR stressed	-0.015	0.095	-0.196	0.172
stressed	AR angry	0.083	0.094	-0.104	0.263
stressed	logvar sad	0.098	0.087	-0.074	0.263
stressed	logvar stressed	-0.444	0.071	-0.570	-0.292
stressed	logvar angry	0.136	0.086	-0.035	0.302
angry	AR sad	-0.037	0.092	-0.216	0.145
angry	AR stressed	-0.158	0.093	-0.332	0.026
angry	AR angry	0.237	0.089	0.054	0.404
angry	logvar sad	-0.011	0.087	-0.182	0.158
angry	logvar stressed	-0.417	0.073	-0.551	-0.264
angry	logvar angry	0.166	0.084	-0.003	0.327
AR sad	AR stressed	0.607	0.069	0.457	0.726
AR sad	AR angry	0.578	0.073	0.421	0.705
AR sad	logvar sad	0.041	0.092	-0.143	0.220
AR sad	logvar stressed	-0.073	0.093	-0.250	0.112
AR sad	logvar angry	-0.012	0.092	-0.195	0.169
AR stressed	AR angry	0.405	0.087	0.224	0.563
AR stressed	logvar sad	-0.045	0.094	-0.234	0.138
AR stressed	logvar stressed	-0.078	0.095	-0.258	0.110
AR stressed	logvar angry	-0.052	0.094	-0.239	0.130
AR angry	logvar sad	0.218	0.091	0.035	0.389
AR angry	logvar stressed	0.036	0.095	-0.149	0.220
AR angry	logvar angry	0.206	0.091	0.021	0.376
logvar sad	logvar stressed	0.566	0.060	0.437	0.672
logvar sad	logvar angry	0.786	0.035	0.708	0.845
logvar stressed	logvar angry	0.473	0.068	0.327	0.596
sad	stressed	0.705	0.045	0.604	0.781
sad	angry	0.883	0.020	0.837	0.916
stressed	angry	0.613	0.055	0.496	0.710

Note: Between-person correlations of individual-specific parameter estimates, for the FEEL study I dataset. Displayed are correlations between the random intercepts, autoregressive effects, and innovation variances, as estimated from Model A for the FEEL dataset. AR: autoregressive effect; LOGV: log innovation variance.

Table S3 Between-person correlations between individual-specific parameter estimatess: Emotional Events

paramHeader	param	est	posterior_sd	lower_2.5ci	upper_2.5ci
sad	AR sad	0.260	0.141	-0.035	0.517
sad	AR stressed	-0.007	0.151	-0.298	0.286
sad	AR angry	0.204	0.145	-0.100	0.474
sad	logvar sad	0.279	0.122	0.022	0.497
sad	logvar stressed	-0.313	0.120	-0.528	-0.059
sad	logvar angry	0.267	0.125	0.005	0.495
stressed	AR sad	0.280	0.139	-0.015	0.529
stressed	AR stressed	0.102	0.149	-0.197	0.387
stressed	AR angry	0.243	0.143	-0.058	0.501
stressed	logvar sad	0.315	0.119	0.061	0.528
stressed	logvar stressed	-0.151	0.129	-0.395	0.104
stressed	logvar angry	0.305	0.121	0.048	0.521
angry	AR sad	0.225	0.143	-0.068	0.492
angry	AR stressed	-0.072	0.151	-0.358	0.227
angry	AR angry	0.283	0.140	-0.011	0.538
angry	logvar sad	0.154	0.128	-0.104	0.393
angry	logvar stressed	-0.322	0.120	-0.534	-0.069
angry	logvar angry	0.336	0.119	0.082	0.545
AR sad	AR stressed	0.429	0.146	0.104	0.676
AR sad	AR angry	0.683	0.108	0.427	0.845
AR sad	logvar sad	0.072	0.147	-0.219	0.355
AR sad	logvar stressed	-0.271	0.141	-0.529	0.019
AR sad	logvar angry	-0.143	0.146	-0.418	0.157
AR stressed	AR angry	0.387	0.149	0.058	0.643
AR stressed	logvar sad	0.040	0.153	-0.265	0.335
AR stressed	logvar stressed	-0.168	0.150	-0.441	0.136
AR stressed	logvar angry	-0.112	0.148	-0.390	0.184
AR angry	logvar sad	0.060	0.145	-0.231	0.335
AR angry	logvar stressed	-0.153	0.145	-0.427	0.142
AR angry	logvar angry	-0.058	0.150	-0.345	0.236
logvar sad	logvar stressed	0.544	0.096	0.336	0.708
logvar sad	logvar angry	0.716	0.067	0.565	0.825
logvar stressed	logvar angry	0.454	0.107	0.223	0.638
sad	stressed	0.680	0.074	0.510	0.798
sad	angry	0.888	0.032	0.810	0.936
stressed	angry	0.654	0.079	0.470	0.781

Note: Between-person correlations of individual-specific parameter estimates, for the Emotional Events dataset. Displayed are correlations between the random intercepts, autoregressive effects, and innovation variances, as estimated from Model A for the EmoEvents dataset. AR: autoregressive effect; LOGV: log innovation variance.

Table S4 Between-person correlations between individual-specific parameter estimatess: AURA

Correlation between				95% CI	
Var 1	Var 2	estimate	posterior SD	lower bound	upper bound
sad	AR sad	0.326	0.092	0.135	0.496
sad	AR anxious	-0.040	0.111	-0.249	0.182
sad	AR dull	-0.088	0.109	-0.302	0.125
sad	AR irritated	0.098	0.114	-0.130	0.317
sad	logvar sad	0.233	0.084	0.063	0.391
sad	logvar anxious	-0.338	0.080	-0.487	-0.173
sad	logvar dull	-0.047	0.089	-0.220	0.130
sad	logvar irritated	-0.142	0.089	-0.314	0.034
anxious	AR sad	0.242	0.093	0.047	0.416
anxious	AR anxious	0.091	0.112	-0.137	0.309
anxious	AR dull	-0.147	0.107	-0.348	0.068
anxious	AR irritated	0.090	0.110	-0.125	0.306
anxious	logvar sad	0.268	0.082	0.096	0.420
anxious	logvar anxious	-0.184	0.085	-0.347	-0.013
anxious	logvar dull	0.046	0.089	-0.131	0.219
anxious	logvar irritated	-0.053	0.089	-0.229	0.121
dull	AR sad	0.432	0.084	0.256	0.584
dull	AR anxious	-0.067	0.112	-0.284	0.155
dull	AR dull	0.204	0.106	-0.012	0.403
dull	AR irritated	0.284	0.105	0.070	0.477
dull	logvar sad	0.203	0.085	0.029	0.361
dull	logvar anxious	-0.159	0.087	-0.325	0.010
dull	logvar dull	0.034	0.089	-0.143	0.205
dull	logvar irritated	-0.144	0.088	-0.311	0.032
irritated	AR sad	0.285	0.092	0.096	0.454
irritated	AR anxious	-0.020	0.111	-0.239	0.197
irritated	AR dull	-0.040	0.111	-0.256	0.180
irritated	AR irritated	0.246	0.111	0.017	0.454
irritated	logvar sad	0.262	0.083	0.092	0.418
irritated	logvar anxious	-0.236	0.084	-0.394	-0.065
irritated	logvar dull	-0.033	0.089	-0.205	0.142
irritated	logvar irritated	0.027	0.090	-0.152	0.200
AR sad	AR anxious	0.393	0.115	0.151	0.599
AR sad	AR dull	0.386	0.107	0.156	0.577
AR sad	AR irritated	0.634	0.090	0.438	0.787
AR sad	logvar sad	0.353	0.092	0.160	0.516
AR sad	logvar anxious	-0.090	0.097	-0.276	0.103
AR sad	logvar dull	0.119	0.098	-0.078	0.306
AR sad	logvar irritated	0.050	0.103	-0.153	0.250
AR anxious	AR dull	0.261	0.130	0.001	0.504
AR anxious	AR irritated	0.488	0.118	0.239	0.692
AR anxious	logvar sad	-0.069	0.111	-0.286	0.154
AR anxious	logvar anxious	-0.111	0.119	-0.345	0.121
AR anxious	logvar dull	0.039	0.114	-0.187	0.264
AR anxious	logvar irritated	-0.011	0.114	-0.239	0.210
AR dull	AR irritated	0.480	0.111	0.238	0.679
AR dull	logvar sad	-0.029	0.110	-0.247	0.187
AR dull	logvar anxious	0.002	0.112	-0.221	0.222
AR dull	logvar dull	0.086	0.110	-0.140	0.293
AR dull	logvar irritated	0.089	0.109	-0.132	0.300
AR irritated	logvar sad	0.112	0.111	-0.111	0.325
AR irritated	logvar anxious	0.015	0.114	-0.206	0.239
AR irritated	logvar dull	-0.185	0.110	-0.393	0.034
AR irritated	logvar irritated	0.134	0.112	-0.095	0.347
logvar sad	logvar anxious	0.340	0.079	0.173	0.484
logvar sad	logvar dull	0.490	0.068	0.343	0.612
logvar sad	logvar irritated	0.394	0.077	0.235	0.532
logvar anxious	logvar dull	0.338	0.080	0.175	0.484
logvar anxious	logvar irritated	0.528	0.066	0.386	0.644
logvar dull	logvar irritated	0.368	0.079	0.203	0.511
sad	anxious	0.767	0.040	0.679	0.834
sad	dull	0.650	0.054	0.531	0.745
sad	irritated	0.762	0.040	0.673	0.828
anxious	dull	0.500	0.069	0.352	0.623
anxious	irritated	0.773	0.039	0.684	0.840
dull	irritated	0.597	0.060	0.467	0.700

Note: Between-person correlations of individual-specific parameter estimates, for the AURA dataset. Displayed are correlations between the random intercepts, autoregressive effects, and innovation variances, as estimated from Model A for the AURA dataset. AR: autoregressive effect; Logvar: log innovation variance; item irritated.

Table S5 Between-person correlations between individual-specific parameter estimatess: Leuven 3-wave data

Correlation between				95% CI	
Var 1	Var 2	estimate	posterior SD	lower bound	upper bound
sad	AR sad	0.099	0.086	-0.075	0.264
sad	AR stressed	-0.034	0.090	-0.211	0.140
sad	AR angry	0.129	0.088	-0.050	0.298
sad	AR fearful	0.211	0.084	0.042	0.370
sad	logvar sad	0.310	0.076	0.155	0.452
sad	logvar stressed	-0.265	0.078	-0.412	-0.107
sad	logvar angry	0.252	0.079	0.090	0.399
sad	logvar fearful	0.420	0.069	0.277	0.546
stressed	AR sad	0.069	0.088	-0.103	0.239
stressed	AR stressed	0.082	0.090	-0.098	0.253
stressed	AR angry	0.098	0.089	-0.080	0.268
stressed	AR fearful	0.122	0.086	-0.049	0.289
stressed	logvar sad	0.327	0.075	0.177	0.467
stressed	logvar stressed	0.032	0.084	-0.134	0.192
stressed	logvar angry	0.313	0.076	0.157	0.454
stressed	logvar fearful	0.456	0.066	0.317	0.576
angry	AR sad	0.016	0.087	-0.158	0.185
angry	AR stressed	-0.089	0.089	-0.263	0.083
angry	AR angry	0.092	0.088	-0.085	0.261
angry	AR fearful	0.181	0.084	0.014	0.343
angry	logvar sad	0.228	0.079	0.069	0.379
angry	logvar stressed	-0.286	0.077	-0.432	-0.130
angry	logvar angry	0.272	0.078	0.112	0.418
angry	logvar fearful	0.411	0.070	0.265	0.539
fearful	AR sad	0.000	0.087	-0.172	0.170
fearful	AR stressed	-0.086	0.090	-0.262	0.088
fearful	AR angry	0.059	0.088	-0.119	0.230
fearful	AR fearful	0.170	0.085	0.000	0.332
fearful	logvar sad	0.192	0.080	0.031	0.345
fearful	logvar stressed	-0.304	0.076	-0.447	-0.148
fearful	logvar angry	0.205	0.080	0.041	0.354
fearful	logvar fearful	0.410	0.070	0.264	0.538
AR sad	AR stressed	0.535	0.073	0.377	0.664
AR sad	AR angry	0.694	0.055	0.573	0.788
AR sad	AR fearful	0.619	0.062	0.487	0.725
AR sad	logvar sad	0.146	0.087	-0.027	0.312
AR sad	logvar stressed	0.015	0.088	-0.156	0.188
AR sad	logvar angry	0.030	0.089	-0.147	0.204
AR sad	logvar fearful	-0.019	0.088	-0.192	0.151
AR stressed	AR angry	0.545	0.075	0.380	0.676
AR stressed	AR fearful	0.584	0.069	0.435	0.703
AR stressed	logvar sad	0.040	0.092	-0.142	0.217
AR stressed	logvar stressed	-0.016	0.093	-0.198	0.164
AR stressed	logvar angry	-0.062	0.092	-0.242	0.117
AR stressed	logvar fearful	-0.018	0.092	-0.200	0.162
AR angry	AR fearful	0.691	0.056	0.569	0.788
AR angry	logvar sad	0.117	0.089	-0.057	0.288
AR angry	logvar stressed	-0.012	0.090	-0.188	0.165
AR angry	logvar angry	-0.064	0.090	-0.240	0.117
AR angry	logvar fearful	-0.043	0.088	-0.213	0.131
AR fearful	logvar sad	0.121	0.086	-0.053	0.287
AR fearful	logvar stressed	-0.135	0.087	-0.300	0.040
AR fearful	logvar angry	-0.010	0.087	-0.178	0.162
AR fearful	logvar fearful	0.035	0.087	-0.133	0.201
logvar sad	logvar stressed	0.598	0.055	0.480	0.696
logvar sad	logvar angry	0.807	0.031	0.737	0.859
logvar sad	logvar fearful	0.738	0.039	0.650	0.805
logvar stressed	logvar angry	0.570	0.058	0.448	0.675
logvar stressed	logvar fearful	0.450	0.068	0.306	0.570
logvar angry	logvar fearful	0.769	0.035	0.691	0.829
sad	stressed	0.771	0.035	0.694	0.830
sad	angry	0.976	0.005	0.963	0.984
sad	fearful	0.974	0.006	0.960	0.983
stressed	angry	0.772	0.035	0.694	0.832
stressed	fearful	0.769	0.035	0.691	0.829
angry	fearful	0.989	0.003	0.982	0.993

Note: Between-person correlations of individual-specific parameter estimates, for the Leuven 3-wave dataset. Displayed are correlations between the random intercepts, autoregressive effects, and innovation variances, as estimated from Model A for the Leuven dataset. AR: autoregressive effect; Logvar: log innovation variance; item irritated.

Table S6 Between-person correlations between individual-specific parameter estimatess: Emotion Regulation Effort data

Correlation between				95% CI	
Var 1	Var 2	estimate	posterior SD	lower bound	upper bound
sad	AR sad	0.153	0.112	-0.075	0.359
sad	AR stressed	-0.160	0.116	-0.377	0.080
sad	AR angry	0.187	0.114	-0.046	0.400
sad	AR anxious	-0.185	0.113	-0.396	0.041
sad	logvar sad	0.331	0.089	0.146	0.494
sad	logvar stressed	-0.230	0.095	-0.407	-0.034
sad	logvar angry	0.410	0.084	0.236	0.564
sad	logvar anxious	-0.180	0.098	-0.361	0.018
stressed	AR sad	0.176	0.110	-0.049	0.382
stressed	AR stressed	-0.160	0.116	-0.377	0.072
stressed	AR angry	0.050	0.116	-0.176	0.278
stressed	AR anxious	-0.096	0.117	-0.325	0.135
stressed	logvar sad	0.309	0.090	0.121	0.474
stressed	logvar stressed	-0.234	0.102	-0.426	-0.032
stressed	logvar angry	0.409	0.086	0.225	0.562
stressed	logvar anxious	-0.055	0.102	-0.256	0.146
angry	AR sad	-0.016	0.115	-0.244	0.206
angry	AR stressed	-0.309	0.112	-0.510	-0.070
angry	AR angry	0.031	0.123	-0.209	0.268
angry	AR anxious	-0.319	0.110	-0.516	-0.091
angry	logvar sad	0.206	0.095	0.015	0.386
angry	logvar stressed	-0.205	0.096	-0.389	-0.010
angry	logvar angry	0.465	0.078	0.299	0.604
angry	logvar anxious	-0.130	0.100	-0.322	0.068
anxious	AR sad	0.175	0.108	-0.048	0.377
anxious	AR stressed	-0.154	0.115	-0.369	0.074
anxious	AR angry	0.139	0.113	-0.088	0.352
anxious	AR anxious	-0.102	0.114	-0.319	0.126
anxious	logvar sad	0.388	0.084	0.214	0.543
anxious	logvar stressed	-0.162	0.097	-0.347	0.029
anxious	logvar angry	0.427	0.082	0.256	0.574
anxious	logvar anxious	-0.074	0.100	-0.264	0.127
AR sad	AR stressed	0.343	0.121	0.087	0.558
AR sad	AR angry	0.333	0.119	0.085	0.548
AR sad	AR anxious	0.387	0.117	0.133	0.588
AR sad	logvar sad	0.085	0.113	-0.139	0.298
AR sad	logvar stressed	-0.120	0.116	-0.339	0.115
AR sad	logvar angry	-0.001	0.114	-0.227	0.222
AR sad	logvar anxious	-0.023	0.114	-0.239	0.203
AR stressed	AR angry	0.418	0.115	0.168	0.619
AR stressed	AR anxious	0.764	0.075	0.587	0.879
AR stressed	logvar sad	0.091	0.115	-0.137	0.309
AR stressed	logvar stressed	0.003	0.137	-0.269	0.262
AR stressed	logvar angry	-0.079	0.116	-0.302	0.153
AR stressed	logvar anxious	0.063	0.120	-0.177	0.294
AR angry	AR anxious	0.361	0.117	0.112	0.569
AR angry	logvar sad	0.200	0.110	-0.025	0.407
AR angry	logvar stressed	0.006	0.115	-0.219	0.230
AR angry	logvar angry	0.263	0.110	0.033	0.465
AR angry	logvar anxious	-0.183	0.113	-0.393	0.050
AR anxious	logvar sad	0.074	0.112	-0.145	0.289
AR anxious	logvar stressed	0.179	0.127	-0.095	0.397
AR anxious	logvar angry	-0.078	0.116	-0.301	0.157
AR anxious	logvar anxious	0.047	0.118	-0.183	0.274
logvar sad	logvar stressed	0.380	0.086	0.200	0.534
logvar sad	logvar angry	0.570	0.068	0.425	0.689
logvar sad	logvar anxious	0.474	0.080	0.302	0.618
logvar stressed	logvar angry	0.346	0.088	0.158	0.504
logvar stressed	logvar anxious	0.770	0.046	0.667	0.846
logvar angry	logvar anxious	0.395	0.086	0.212	0.550
sad	stressed	0.713	0.051	0.598	0.798
sad	angry	0.817	0.039	0.730	0.880
sad	anxious	0.756	0.046	0.653	0.831
stressed	angry	0.593	0.067	0.446	0.706
stressed	anxious	0.925	0.018	0.882	0.953
angry	anxious	0.582	0.069	0.429	0.701

Note: Between-person correlations of individual-specific parameter estimates, for the Emotion Regulation Effort dataset. Displayed are correlations between the random intercepts, autoregressive effects, and innovation variances, as estimated from Model A for the Emotion Regulation Effort dataset. AR: autoregressive effect; Logvar: log innovation variance; item irritated.

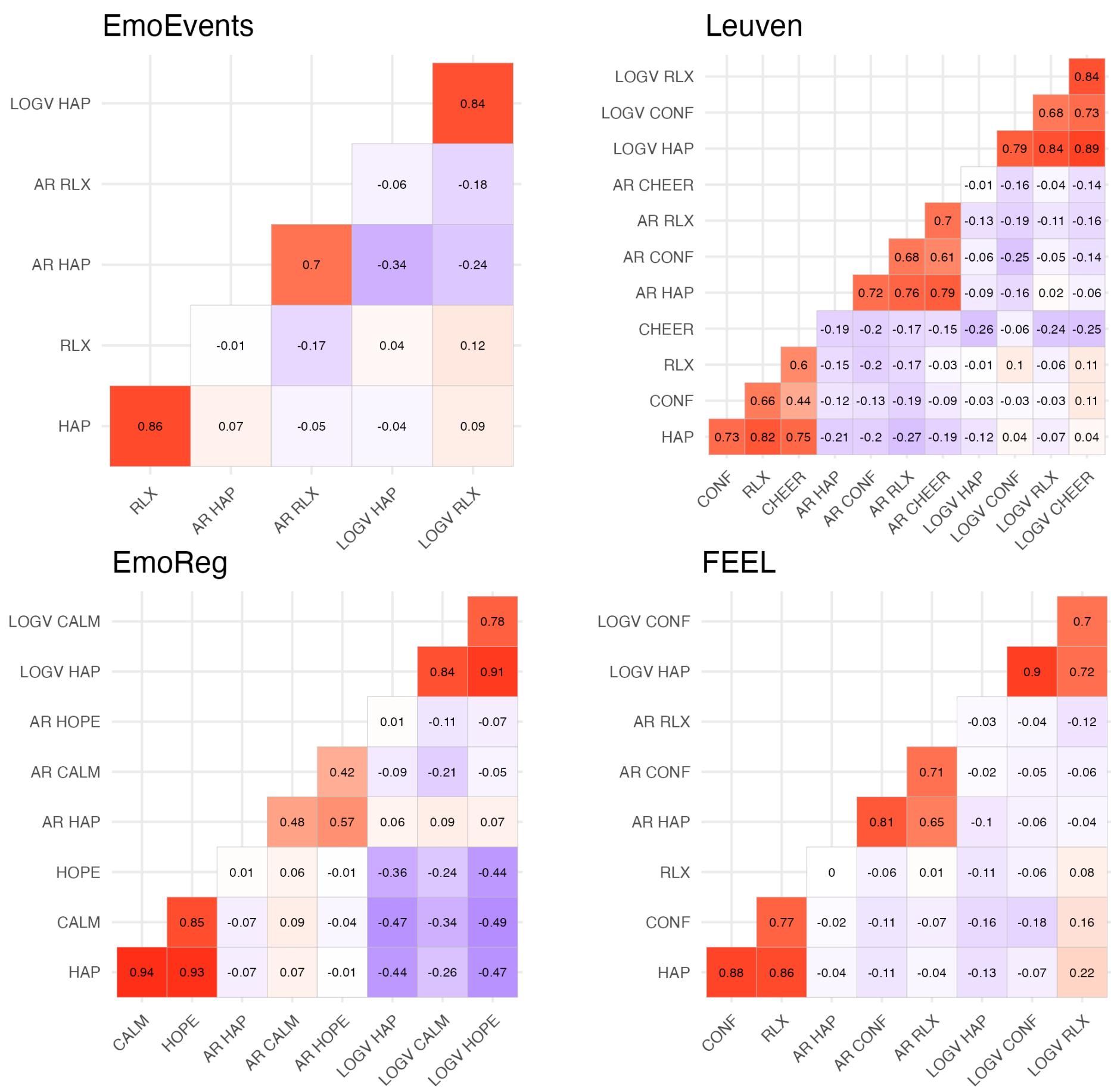

Fig. S1 Results Model A, positive affect. Heatmap of between-person correlations between individual-specific parameter estimates, grouped by dataset. Displayed are correlations between the random intercepts, autoregressive effects, and innovation variances. AR: autoregressive effect; LOGV: log innovation variance; HAP: item happy; RLX: item relaxed; CHEER: item cheerful; CONF: item confident; CALM: item calm; HOPE: item hopeful.

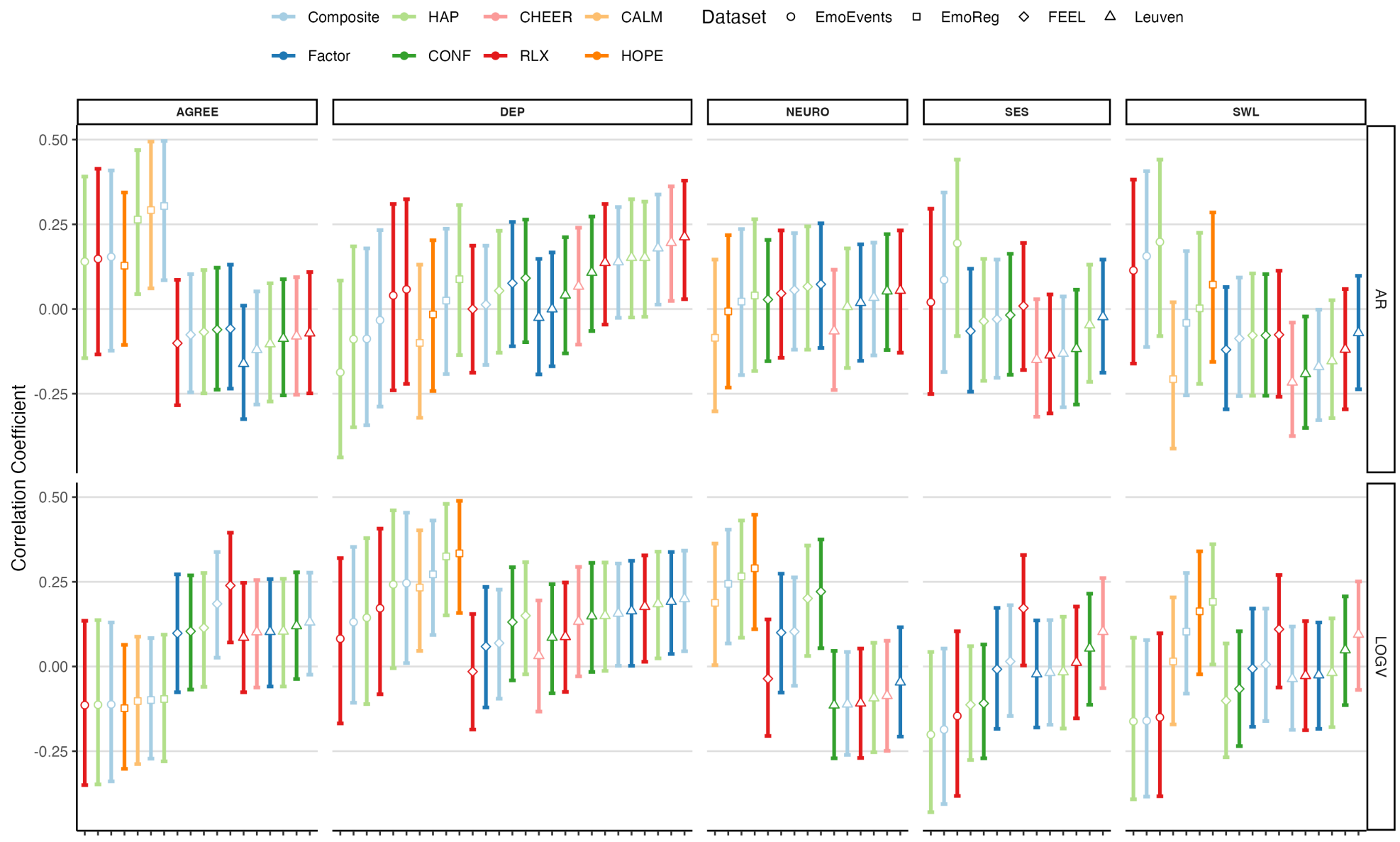

Fig. S2 Results Model A, B, and C, positive affect. Posterior medians and 95% credibility intervals of correlations between multiple outcome variables and random item-specific autoregressive (AR) effects (upper Panels) and the respective log-transformed innovation variances (LOGV, lower Panels). Items / factor / composite score are colour-coded, datasets are represented by symbols, and columns represent different outcome variables. Sample sizes used for the analysis were: AURA n = 173, EmoEvents n = 102, EmoReg n = 146, FEEL n = 179, and Leuven n = 202. Composite: results based on the composite score calculated by averaging across the negative affect items of a respective dataset in Model B; Factor: results based on a common latent within-level factor model (Model C); HAP: happy; CHEER: cheerful; CONF: confident; RLX: relaxed; HOPE: hopeful; AGREE: agreeableness; DEP: depression; NEURO: neuroticism; SES: self-esteem; SWL: satisfaction with life.

Table S7 Associations between contemporaneous item specificity coefficients across negative affect items

Items	dataset	correlation	95% CI
angry - stressed	Leuven	.691	[.611; .757]
	FEEL	.483	[.362; .588]
	EmoEvents	.668	[.544; .763]
	EmoReg	.548	[.423; .652]
fearful - stressed	Leuven	.675	[.592; .744]
anxious - stressed	EmoReg	.908	[.875; .933]
fearful - angry	Leuven	.611	[.516; .691]
anxious - angry	EmoReg	.582	[.463; .680]
anxious - dull	AURA	.377	[.242; .498]
anxious - irritated	AURA	.506	[.386; .609]
dull - irritated	AURA	.656	[.562; .733]

Notes. Item specificity coefficients were calculated based on the parameter estimates from Model D, using 'sad' as the reference item, and correlation coefficients along with their confidence intervals were subsequently estimated in R. Est.: Estimate; CI: Confidence interval

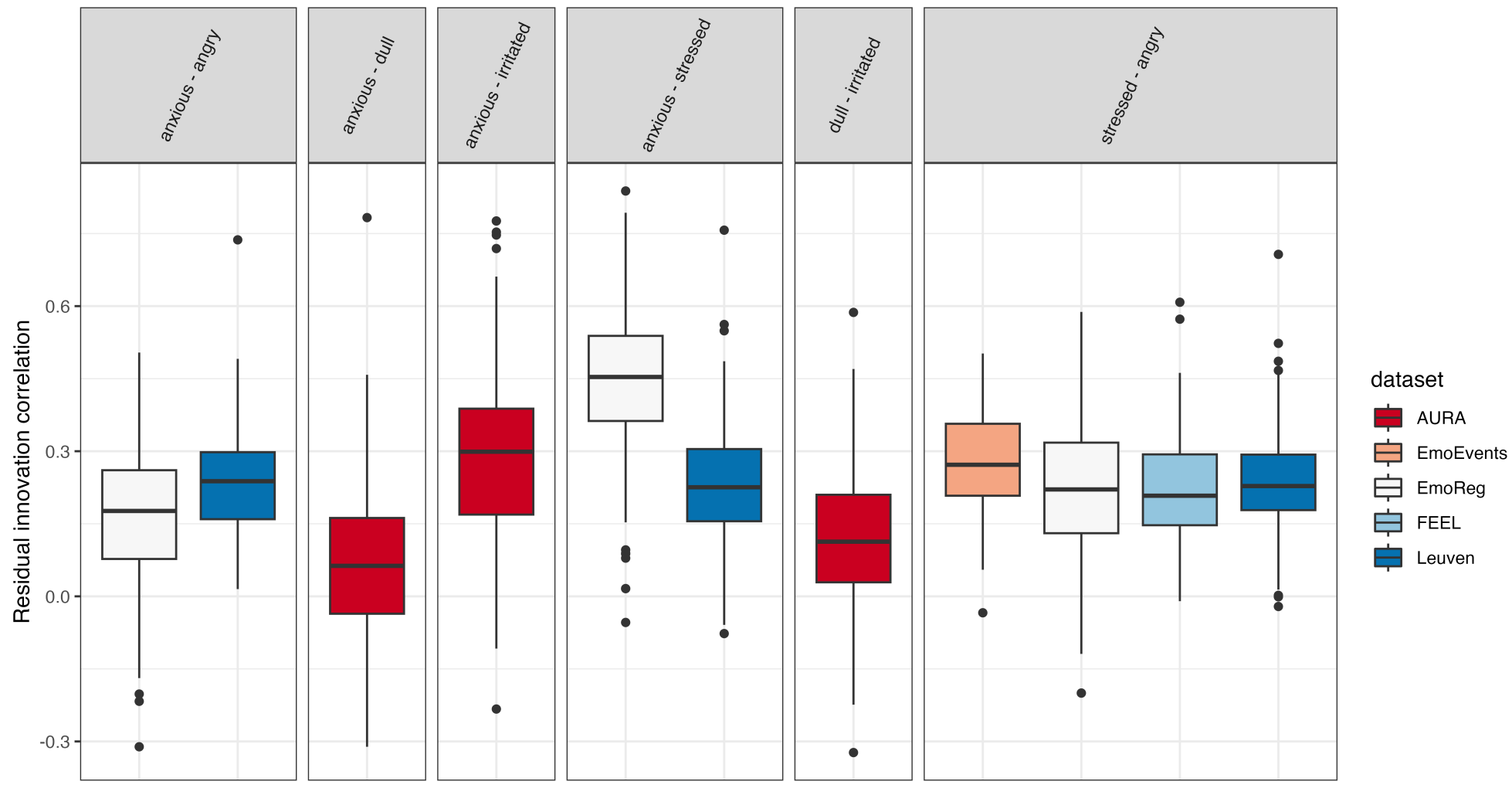

Fig. S3 Results Model D, negative affect. Boxplots of individual-specific contemporaneous correlations between non-reference items' residual innovations. Columns represent different combinations of affect items, datasets are colour-coded. Boxplots are based on the following sample sizes: AURA n = 173, EmoEvents n = 102, EmoReg n = 146, FEEL n = 179, and Leuven n = 202.

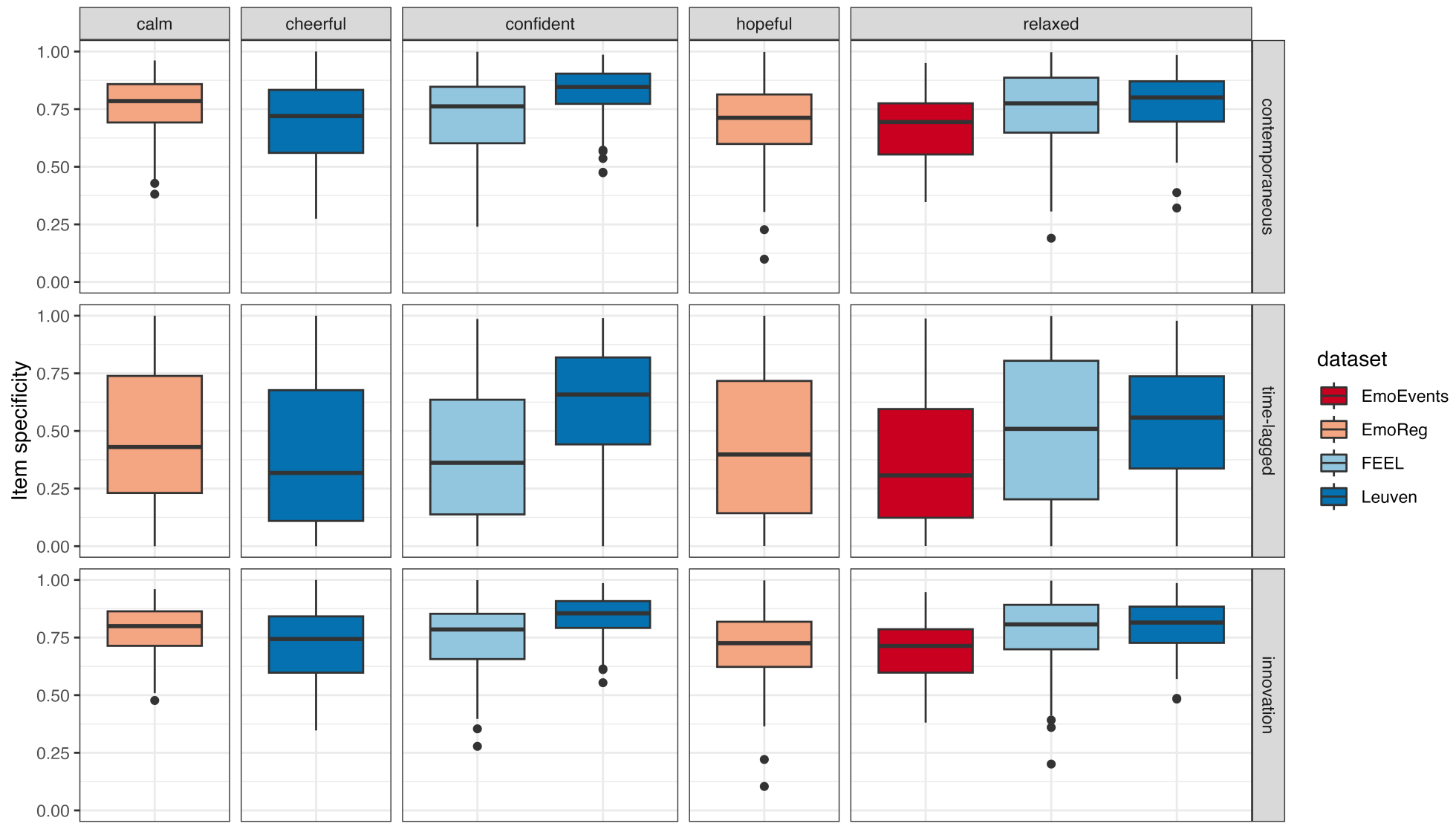

Fig. S4 Results Model D, positive affect. Boxplots of individual-specific item-specificity coefficients. Item specificity is calculated with respect to the reference item "happy". Columns present the different non-reference items, datasets are colour-coded. Item specificity is calculated for the contemporaneous relations between items, the time-lagged associations, and the innovation components. Boxplots are based on the following sample sizes: AURA n = 173, EmoEvents n = 102, EmoReg n = 146, FEEL n = 179, and Leuven n = 202.

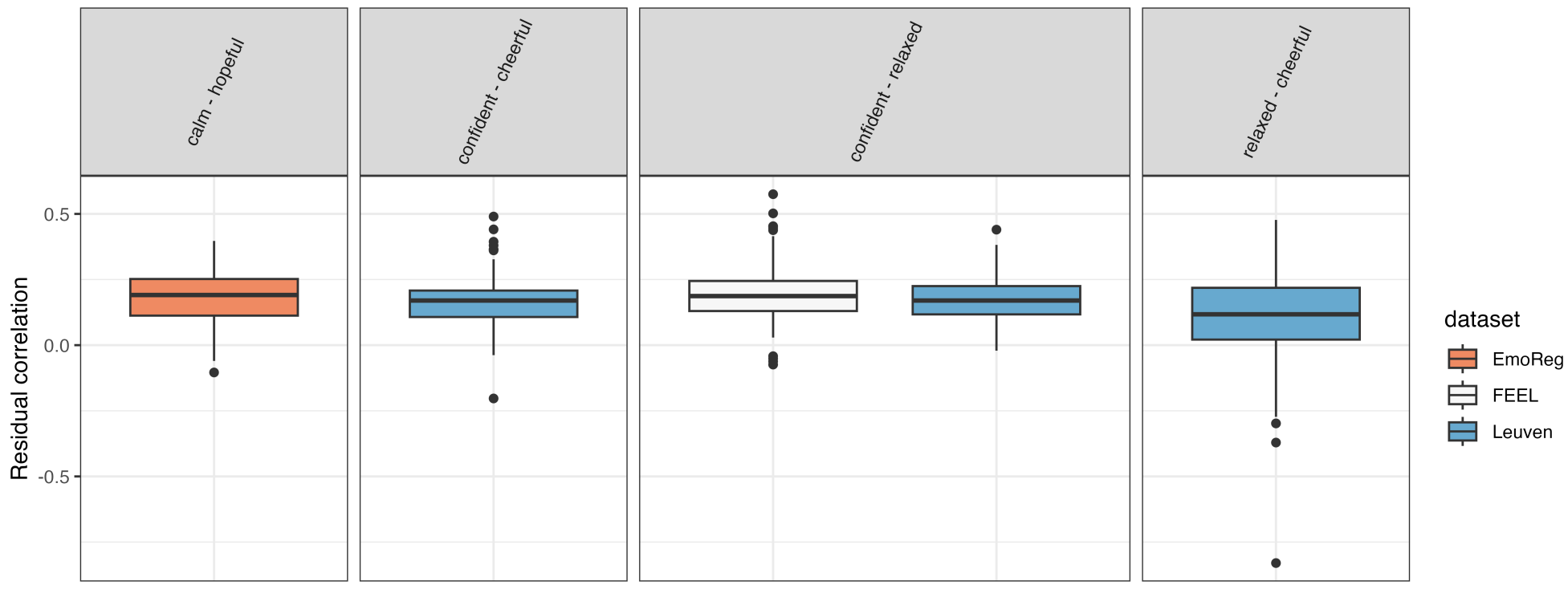

Fig. S5 Results Model D, positive affect. Boxplots of individual-specific contemporaneous correlations between non-reference items' residual innovations. Columns represent different combinations of affect items, datasets are colour-coded. Boxplots are based on the following sample sizes: AURA n = 173, EmoEvents n = 102, EmoReg n = 146, FEEL n = 179, and Leuven n = 202.
